# Zebrafish Model for Functional Screening of Flow-Responsive Genes

**DOI:** 10.1161/ATVBAHA.116.308502

**Published:** 2016-12-21

**Authors:** Jovana Serbanovic-Canic, Amalia de Luca, Christina Warboys, Pedro F. Ferreira, Le A. Luong, Sarah Hsiao, Ismael Gauci, Marwa Mahmoud, Shuang Feng, Celine Souilhol, Neil Bowden, John-Paul Ashton, Henning Walczak, David Firmin, Rob Krams, Justin C. Mason, Dorian O. Haskard, Spencer Sherwin, Victoria Ridger, Timothy J.A. Chico, Paul C. Evans

**Affiliations:** From the Department of Infection, Immunity and Cardiovascular Disease (J.S.-C., L.A.L., S.H., I.G., M.M., S.F., C.S., N.B., J.-P.A., V.R., T.J.A.C., P.C.E.), INSIGNEO Institute for In Silico Medicine (J.S.-C., V.R., T.J.A.C., P.C.E.), and the Bateson Centre (J.S.-C., J.-P.A., T.J.A.C., P.C.E.), University of Sheffield, United Kingdom; and Departments of Cardiovascular Science (A.d.L., C.W., J.C.M., D.O.H.), Imaging (P.F.F., D.F.), Bioengineering (R.K.), and Aeronautics (S.S.) Imperial College London, United Kingdom; and Cancer Institute, Faculty of Medical Sciences (H.W.), University College London, United Kingdom.

**Keywords:** apoptosis, atherosclerosis, endothelial cells, shear stress, Zebrafish

## Abstract

**Objective—:**

Atherosclerosis is initiated at branches and bends of arteries exposed to disturbed blood flow that generates low shear stress. This mechanical environment promotes lesions by inducing endothelial cell (EC) apoptosis and dysfunction via mechanisms that are incompletely understood. Although transcriptome-based studies have identified multiple shear-responsive genes, most of them have an unknown function. To address this, we investigated whether zebrafish embryos can be used for functional screening of mechanosensitive genes that regulate EC apoptosis in mammalian arteries.

**Approach and Results—:**

First, we demonstrated that flow regulates EC apoptosis in developing zebrafish vasculature. Specifically, suppression of blood flow in zebrafish embryos (by targeting cardiac troponin) enhanced that rate of EC apoptosis (≈10%) compared with controls exposed to flow (≈1%). A panel of candidate regulators of apoptosis were identified by transcriptome profiling of ECs from high and low shear stress regions of the porcine aorta. Genes that displayed the greatest differential expression and possessed 1 to 2 zebrafish orthologues were screened for the regulation of apoptosis in zebrafish vasculature exposed to flow or no-flow conditions using a knockdown approach. A phenotypic change was observed in 4 genes; p53-related protein (*PERP*) and programmed cell death 2–like protein functioned as positive regulators of apoptosis, whereas angiopoietin-like 4 and cadherin 13 were negative regulators. The regulation of *perp*, *cdh13*, *angptl4*, and *pdcd2l* by shear stress and the effects of *perp* and *cdh13* on EC apoptosis were confirmed by studies of cultured EC exposed to flow.

**Conclusions—:**

We conclude that a zebrafish model of flow manipulation coupled to gene knockdown can be used for functional screening of mechanosensitive genes in vascular ECs, thus providing potential therapeutic targets to prevent or treat endothelial injury at atheroprone sites.

## Abstract 2

Supplemental Digital Content is available in the text.

Endothelial cell (EC) responses to wall shear stress (WSS), a force exerted on the endothelium by flowing blood, play a crucial role in vascular homeostasis and also contribute to arterial disease. Plaque formation occurs at branches and bends exposed to disturbed blood flow that generates sites of hemodynamic stasis and WSS with low magnitude and variations in direction (oscillations).^1,2^ These hemodynamic conditions promote atherosclerosis by inducing EC apoptosis and dysfunction.^3,4^ This is highly relevant to atherosclerosis pathophysiology because EC apoptosis initiates lesion development at sites of low WSS^5–13^ and promotes plaque erosion^1,14^ via mechanisms that are only partially understood.^9–13,15^ By contrast, regions of arteries exposed to uniform blood flow are protected because high WSS at these sites maintains EC in a quiescent state. Previous studies of the EC transcriptome revealed that flow alters the expression of hundreds of genes, but the function of the majority of them is unknown.^16–19^ Therefore, new strategies to identify the function of flow-modulated genes and their role in vascular physiology are urgently required. Screening gene function represents a powerful and unbiased approach used widely to study cellular responses to biochemical signals. However, to our knowledge, functional screening of cells exposed to mechanical force has not been reported.

**See cover image**

The zebrafish is a unique vertebrate model that combines advantages characteristic of invertebrate models (small size, powerful genetic tractability, high fecundity, ease of maintenance, and relatively low cost) with a high degree of evolutionary conservation with mammals. Thus zebrafish are invaluable not only for studying vertebrate development and physiology but also for modeling human diseases. Although zebrafish embryos have been used extensively to study the effects of flow on angiogenesis and other developmental processes,^20^ they have been used less frequently to study adult vasculature. Here, we tested the hypothesis that flow regulates EC physiology through mechanisms that are, at least in part, conserved between in adult mammalian arteries and developing zebrafish vasculature. If correct, then zebrafish embryos could provide a valuable model for studying the function of genes that are coexpressed in adult mammalian arteries. We tested this by examining whether zebrafish embryos can be used to identify genes that regulate apoptosis in adult mammalian arteries under different hemodynamic conditions. Our studies revealed that flow is a potent regulator of EC apoptosis in developing zebrafish vasculature. A panel of flow-sensitive input genes expressed in mammalian arteries was assembled by transcriptome profiling of ECs from high and low WSS regions of the porcine aorta. Gene silencing of this panel in zebrafish embryos led to the identification of 4 genes that regulated apoptosis: *perp*(p53-related protein) and *pdcd2l*(programmed cell death 2–like protein), which functioned as positive regulators of apoptosis under conditions of flow cessation, whereas *cdh13* (cadherin 13) and *angptl4* (angiopoietin-like 4) exerted antiapoptotic function. The regulation of PERP, PDCD2L, CDH13, and ANGPTL4 by shear stress was confirmed by en face staining of the murine endothelium and by using EC exposed to flow. The ability of PERP and CDH13 to regulate EC apoptosis was confirmed by gene silencing studies in cultured human EC. We conclude that mechanosensitive pathways that control EC apoptosis are partially conserved between zebrafish embryos and mammalian systems. Thus, zebrafish embryos may provide a useful model for functional screening of mechanosensitive pathways.

## Materials and Methods

Materials and Methods are available in the online-only Data Supplement.

## Results

### Zebrafish Model to Study Endothelial Apoptosis Regulation by Hemodynamic Forces

We wished to know whether flow regulates EC apoptosis in the vasculature of zebrafish embryos. This was addressed by manipulating flow that normally commences with cardiac contraction at ≈24 hours post fertilization (hpf). To study hemodynamic responses in embryos, blood flow was blocked either by using *silent heart* (*sih*) antisense morpholino oligonucleotide (MO), which targets cardiac troponin T2 leading to a nonbeating heart^21^ or by treating embryos with the anesthetic tricaine to stop cardiac contraction. It should be noted that embryos lacking blood flow remain viable for up to 5 days because a sufficient supply of oxygen and nutrients is provided by diffusion.^22^ Consistent with this, we did not observe a hypoxic response in *sih* embryos during development using a hypoxia reporter line *phd3:GFP*^23^ (Figure I in the online-only Data Supplement) and zebrafish embryos lacking blood flow did not upregulate hypoxia responsive genes.^24^

**Figure 1.**
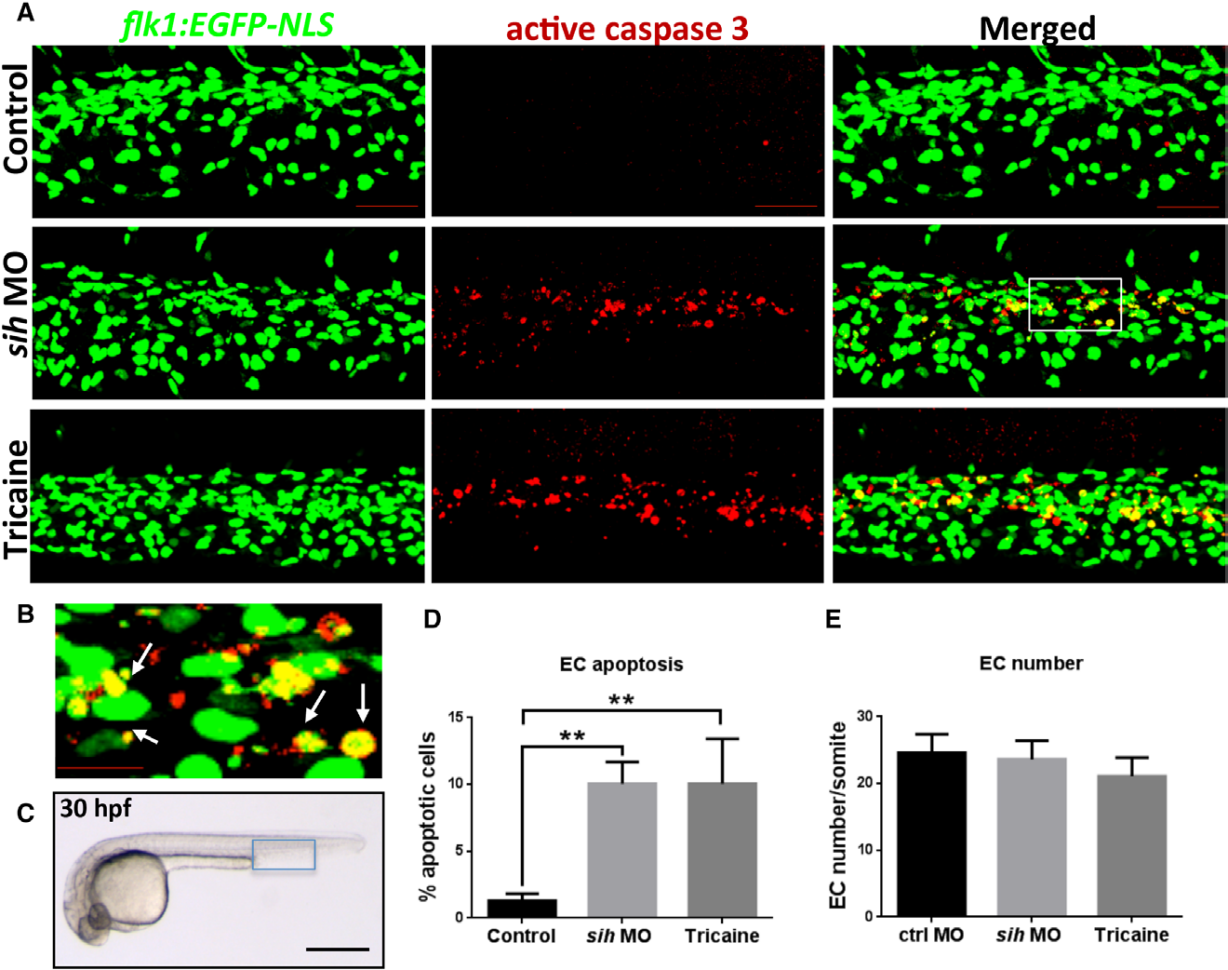
Flow cessation induces endothelial cell (EC) apoptosis in zebrafish embryos. **A**, Whole-mount active caspase-3 (red) staining of 30 hours post fertilization (hpf) *flk1:EGFP-NLS* zebrafish embryos (green EC nuclei) in the presence (control) or absence of flow (*sih* morpholino oligonucleotide [MO], tricaine). The region outlined with the white box is shown in higher magnification in **B**; white arrows indicate apoptotic ECs (yellow). **C**, Zebrafish embryo at 30 hpf. The region outlined with blue box represents the region that is studied in **A**. The percentage of EC apoptosis (**D**) and EC numbers (**E**) in *sih* MO-injected and tricaine-treated embryos compared with controls was quantified, and mean values are shown with SD; n≥15 from 3 independent experiments, **P<0.01 using 1-way ANOVA. **A**–**C**, Lateral view, anterior to the left, dorsal up. Scale bars, 50 μm (**A**), 15 μm (**B**), and 500 μm (**C**).

EC apoptosis was assessed in transgenic *flk1:EGFP-NLS* embryos^25^ (green fluorescent protein^+^ EC nuclei) by active caspase-3 immunohistochemistry (Figure 1A through 1C) or by TUNEL (terminal deoxynucleotidyl transferase dUTP nick-end labeling) assay (Figure II in the online-only Data Supplement). Increased EC apoptosis was observed at 30 to 32 hpf in the aorta and caudal vein plexus of embryos lacking blood flow (Figure 1A through 1D; Figure II in the online-only Data Supplement), whereas total EC numbers were comparable to controls (Figure 1E). At 48 hpf, apoptosis was almost completely resolved in embryos lacking flow, but EC numbers were decreased and caudal vein geometry was less complex than controls at this time point (Figure III in the online-only Data Supplement). We conclude that the suppression of flow triggers a transient wave of EC apoptosis accompanied by EC loss and altered vascular remodeling. On the contrary, blood flow drives EC survival during zebrafish development.

### Identification of Putative Shear-Responsive Regulators of Apoptosis by Transcriptional Profiling of the Porcine Aorta

**Figure 2.**
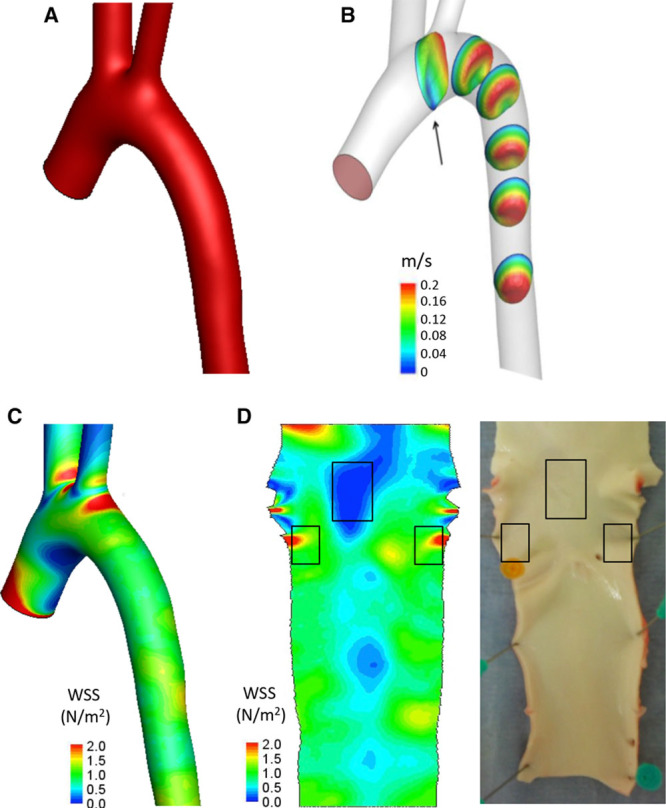
Steady state fluid dynamics in the porcine aorta. Steady state fluid dynamics in the porcine aorta were studied using magnetic resonance (MR) imaging and computational fluid dynamics. Five animals were studied, and representative data are shown from a single animal. **A**, The surfaces of the aortic arch were reconstructed from MR images, smoothed with a low-pass filter, and extended with cylindrical flow extensions at the outlets. **B**, Three-dimensional velocity contours are shown over 6 representative planes. **C**, A time-averaged wall shear stress (WSS) map was calculated and mapped onto the aortic geometry. High WSS is represented in red and low WSS in blue. **D**, The time-averaged WSS map was unwrapped via a computational incision over the outer aortic wall, and the 2-dimensional WSS map was visualized with the endothelial layer facing upward (**left**). The aorta was cut along the outer curvature to expose the lumen, and the low and high WSS regions were identified by reference to the WSS map (indicated with boxes).

Given that enhanced EC apoptosis was observed in embryonic zebrafish vasculature exposed to static conditions (Figure 1) and in adult mammalian arteries exposed to low WSS,^26^ we hypothesized that zebrafish embryos may be used for screening of mechanosensitive genes that regulate apoptosis in adult arteries. To test this, we generated a panel of candidate regulators of apoptosis by transcriptome profiling of ECs from low and high WSS regions of the porcine aorta. Healthy pigs aged 6 months were used to allow identification of genes that predispose low WSS sites to disease. Although pigs have been used extensively to study focal atherogenesis, WSS in the porcine aorta has not been defined previously. Therefore, we used magnetic resonance imaging and computational fluid dynamics modeling to characterize flow and WSS in the porcine aortic arch (Figure 2; Figures IV and V in the online-only Data Supplement). Steady state simulations revealed velocity profiles skewed toward the outer wall and rotated toward the anterior wall leading to higher WSS on the outer wall compared with the inner wall (Figure 2; Figure IV in the online-only Data Supplement). Unsteady state simulations were run over multiple cardiac cycles using a single geometry and periodicity was reached at the fourth cycle (Figure V in the online-only Data Supplement). Velocity profiles were computed at multiple locations of the aortic arch at four representative time points of the cardiac cycle (Figure V in the online-only Data Supplement). The curvature of the arch introduced a rotation of the velocity profile clearly visible in the deceleration and diastolic phase. At peak systole, the presence of the branches caused a reflection of the incoming high-velocity flow that was directed toward the inner wall of the arch. It is also important to note the presence of retrograde flow (Figure VB in the online-only Data Supplement, arrow) starting at the late systole and reaching the maximum at peak diastole, as previously observed in human studies.^27,28^ Time-averaged WSS was similar to steady simulations with the inner curvature of the arch being exposed to WSS with low-magnitude and high oscillatory shear index (Figure V in the online-only Data Supplement).

**Figure 3.**
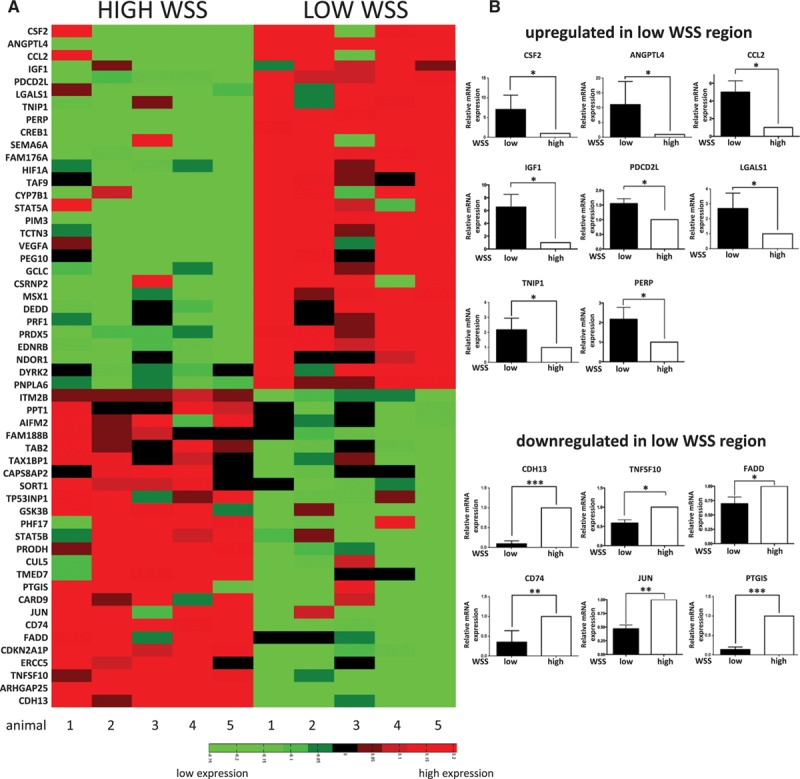
Transcriptome profiling of the porcine aorta. The endothelial cell (EC) transcriptome was studied at the low and high wall shear stress (WSS) regions of the porcine aorta using microarrays. Five pigs were studied. Aortae were cut along the outer curvature to expose the lumen, and ECs were isolated from the low and high WSS regions. **A**, Genes with a known or putative role in apoptosis are presented as a heat map representing expression patterns at the low and high WSS sites. Red indicates enrichment in gene expression, whereas green indicates suppression. **B**, Validation of microarray data by quantitative reverse transcriptase-PCR (qRT-PCR). The 20 most differentially regulated apoptotic genes were selected for validation in an independent cohort of pigs. Transcript levels were quantified by qRT-PCR using gene-specific primers. Mean values are shown with SD; n=5, *P<0.05, **P<0.01, ***P<0.001 using an unpaired 2-tailed test.

Based on our computational fluid dynamics model, ECs were isolated from high and low time-averaged WSS regions of the porcine aorta (Figure 2D) using collagenase before extraction of RNA. The integrity of RNA samples was confirmed (Figure VI in the online-only Data Supplement) and quantitative reverse transcriptase-PCR (qRT-PCR) revealed high expression of CD31 (EC marker) and negligible quantities of smooth muscle cell or macrophage (CD14) markers (Figure VI in the online-only Data Supplement). RNA samples were labeled and hybridized against GeneChip Porcine Genome Arrays (Affymetrix), which revealed 867 genes to be shear responsive (Table I in the online-only Data Supplement). Functional annotation found that 494 genes have a known or putative function, and molecules with an inferred or known role in the regulation of apoptosis showed maximal enrichment (Table II in the online-only Data Supplement). The expression of putative apoptosis regulators at high and low WSS sites was visualized using a heat map (Figure 3A), and we selected the 20 genes with greatest differential expression for further analysis. Out of these, we validated differential expression of 14 genes by qRT-PCR in an independent cohort of pigs (Figure 3B), whereas differential expression was inconsistent for 6 genes (data not shown). Thus, 14 genes with validated differential expression were selected as candidate regulators of apoptosis for functional screening in zebrafish.

### Functional Screening of Apoptotic Regulators in Zebrafish

We further selected genes for functional screening based on the existence of 1 to 2 orthologues in zebrafish. From the 14 candidate apoptotic regulators, 2 genes (*CCL2* and *CSF2*) were excluded because they have no orthologues in zebrafish. By contrast, *LGALS1* has 3 zebrafish orthologues (Table III in the online-only Data Supplement) and was excluded from further analysis because of possible redundancy between the 3 paralogues, which would make functional analysis difficult. One of the candidate genes, *CD74*, had 2 zebrafish orthologues, *cd74a* and *cd74b*, and both of these were included in the functional screening study. Therefore, a total of 12 zebrafish genes were selected for functional screening (Table III in the online-only Data Supplement). To test whether the candidate genes are expressed in the zebrafish endothelium, we isolated ECs from 26 hpf *flk1:EGFP-NLS* embryos using fluorescence-activated cell sorting (Figure VII in the online-only Data Supplement). The vascular identity of purified green fluorescent protein^+^ cells was confirmed by enriched expression of the EC marker *cdh5* (vascular endothelial cadherin), whereas sorted green fluorescent protein^−^ cells were used as a control (Figure VIIB in the online-only Data Supplement, upper panels). We detected endothelial expression of all 12 candidate genes, with some genes being particularly abundant (ie, *angptl4*, *perp*, *tnip1*; Figure VIIB in the online-only Data Supplement, center and lower panels).

**Figure 4.**
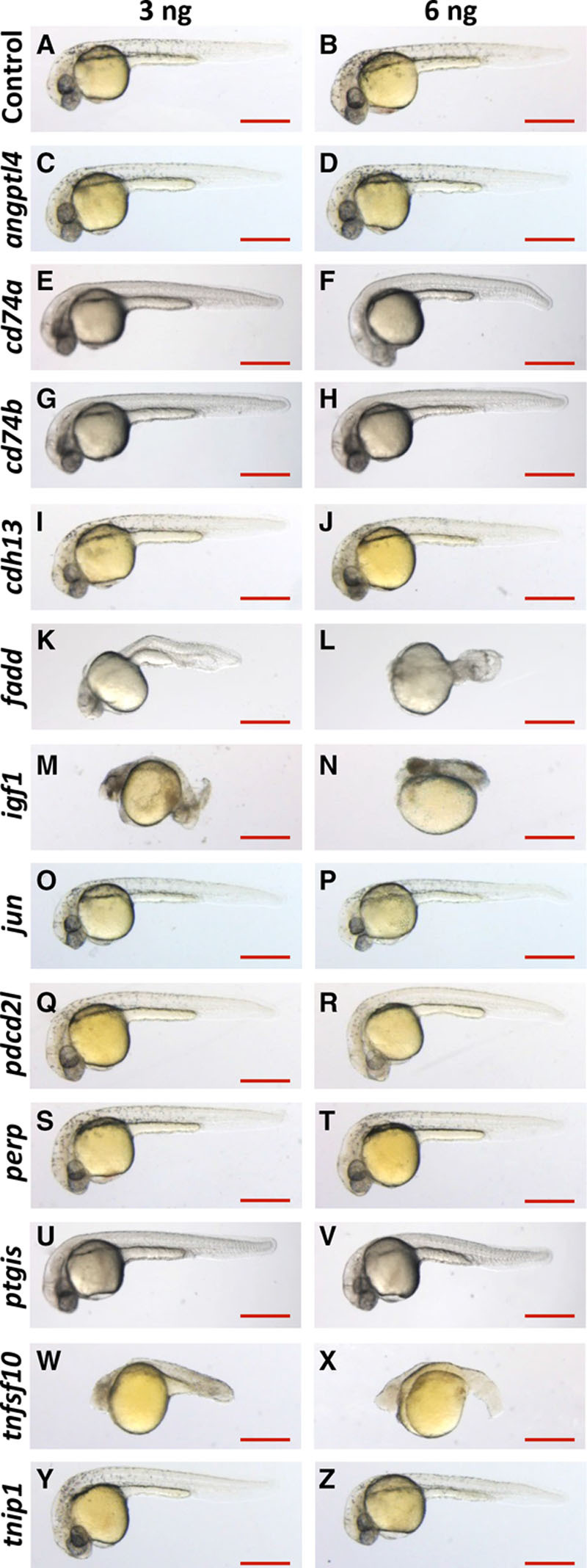
Morphology of morpholino oligonucleotide (MO)–injected embryos. **A**–**Z**, Zebrafish embryos were injected with 3 ng (**left**) or 6 ng (**right**) of gene-specific or nontargeting control MO (indicated on the **left**). Embryo morphology was observed during development and is shown here at 30 hours post fertilization. Lateral view, anterior to the left, dorsal up. Scale bar: 500 μm.

Antisense MOs were used to transiently knock down the expression of candidate genes to assess their function in zebrafish embryos. We initially performed dose–response experiments to determine an optimal dose of each MO for gene knockdown and to assess gross effects on embryogenesis. Knockdown of 3 genes (*fadd*, *igf1*, and *tnfsf10*) resulted in embryonic abnormalities even at a relatively low MO dose (Figure 4K, 4M, and 4W). Because of difficulties in distinguishing between direct and indirect effects of gene knockdown in these embryos, *fadd*, *igf1*, and *tnfsf10* were excluded from further analysis. For the remaining 9 genes, knockdown embryos showed normal morphology comparable to control embryos (Figure 4). The efficiency of splice-blocking MOs was determined by RT-PCR and qRT-PCR (Figure VIII in the online-only Data Supplement). Alternatively spliced transcripts in MO-injected samples were observed as a band shift after gel electrophoresis of RT-PCR products (Figure VIII in the online-only Data Supplement), and these transcripts were confirmed by sequencing to contain a frameshift leading to a premature stop codon (data not shown). For *angptl4*, *fadd*, *perp*, and *tnip1*, a reduced level of the wild-type transcript was observed in the MO-injected samples, and qRT-PCR was consequently used to assess the efficiency of the knockdown (Figure VIII in the online-only Data Supplement).

**Figure 5.**
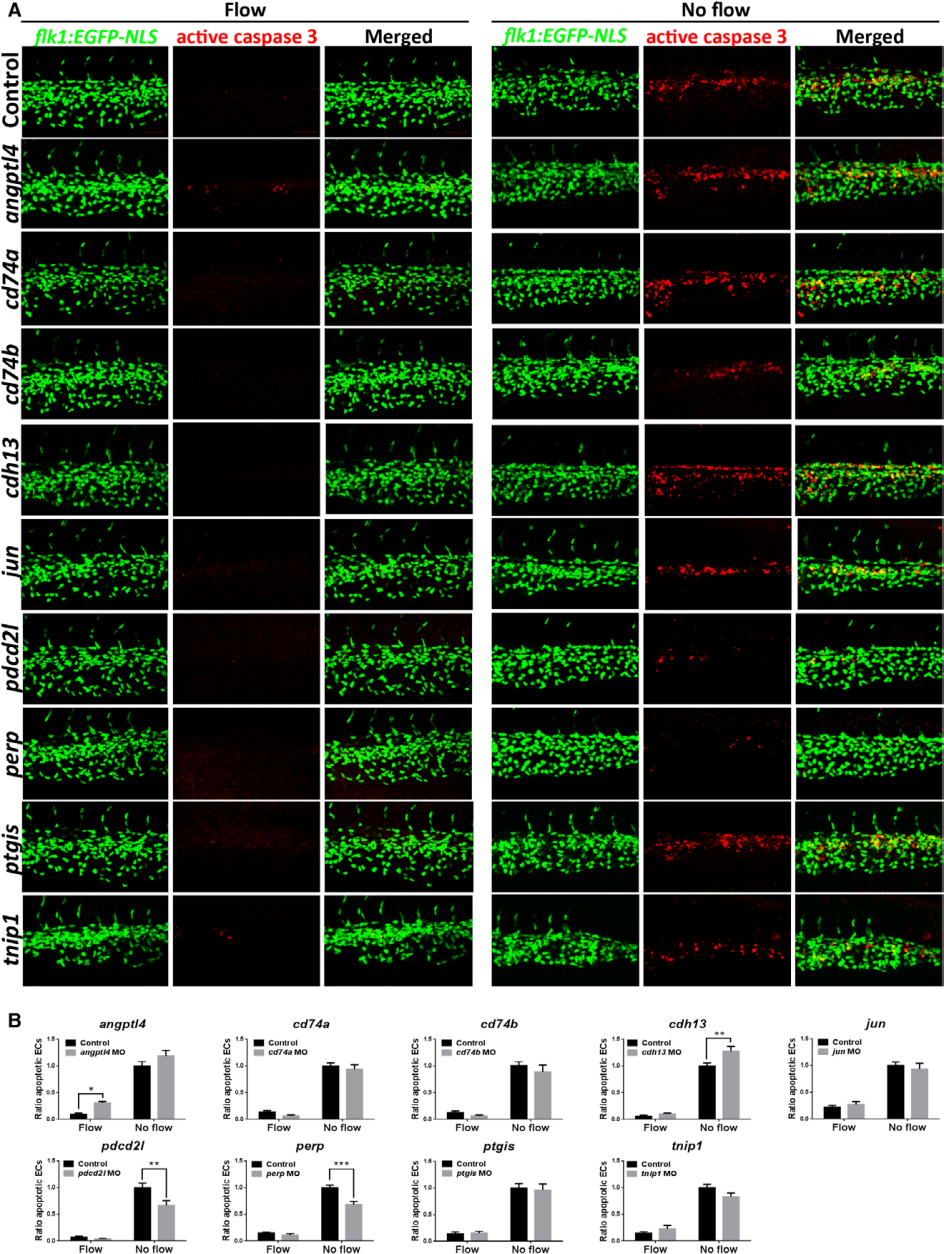
Zebrafish functional screening of putative apoptotic regulators. **A**, Zebrafish embryos (*flk1:EGFP-NLS* embryos; green endothelial cell [EC] nuclei) were injected with morpholinos oligonucleotides (MOs) targeting candidate genes or a nontargeting control MO (indicated on the **left** of each row). EC apoptosis was studied in the presence (control MO) or in the absence (*sih* MO) of flow by whole-mount active caspase-3 staining (red). Apoptotic ECs (yellow) were monitored at 30 hours post fertilization (hpf). Lateral view, anterior to the left, dorsal up. Scale bar: 50 μm. **B**, The proportion of apoptotic ECs (number of apoptotic ECs divided by the total number of ECs) normalized to *sih* MO-injected embryos was calculated, and mean values are shown with SEM. n≥15 from 3 independent experiments. *P<0.05, **P<0.01, ***P<0.001 using 1-way ANOVA.

After knockdown of specific genes using MOs, EC apoptosis was assessed in embryos by staining of active caspase-3 in the presence (control MO) or in the absence of flow (Figure 5). Knockdown of 5 genes (*cd74a*, *cd74b*, *jun*, *ptgis*, and *tnip1*) did not modify EC apoptosis (Figure 5) or EC numbers (Figure IXB, IXC, IXE, IXH, and IXI in the online-only Data Supplement) either in the presence or in the absence of blood flow.

Knockdown of *cdh13* resulted in an increase in EC apoptosis in the absence of flow (Figure 5; Figure IXD in the online-only Data Supplement), whereas depletion of *angptl4* increased apoptosis in the presence of flow (Figure 5; Figure IXA in the online-only Data Supplement). These data suggest that although *cdh13* and *angptl4* exert antiapoptotic effects, they function under different mechanical conditions. By contrast, depletion of *perp* or *pdcd2l* led to a profound decrease (>30%) in EC apoptosis in the absence of flow (Figure 5; Figure IXF and IXG in the online-only Data Supplement) suggesting that these genes are proapoptotic.

**Figure 6.**
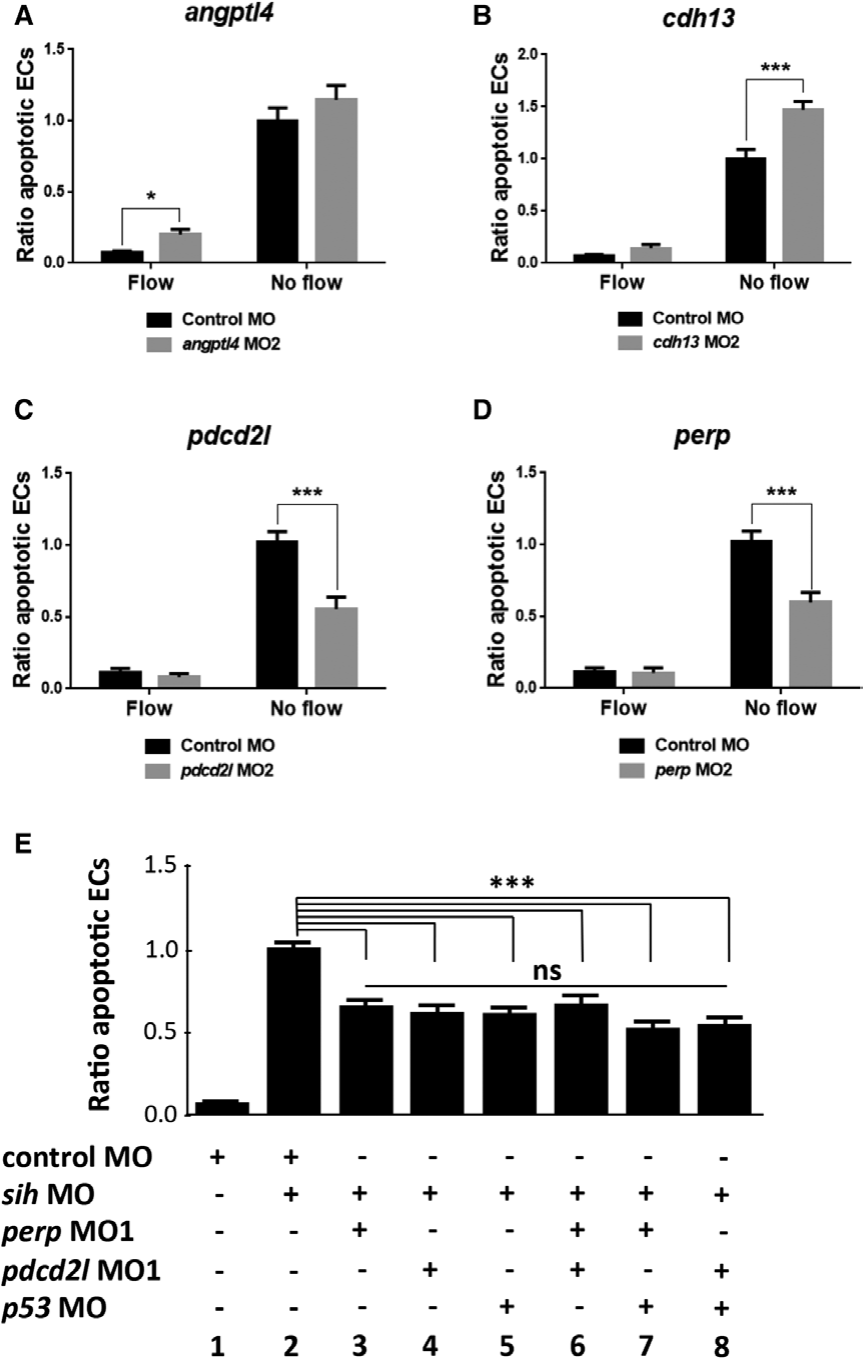
Validation of knockdown phenotypes and mechanistic studies of proapoptotic regulators. *Angptl4* (**A**), *cdh13* (**B**), *pdcd2l* (**C**), and *perp* (**D**) knockdown phenotypes were validated by injecting zebrafish embryos (*flk1:EGFP-NLS*) using a second nonoverlapping morpholino oligonucleotide (termed MO2) or a nontargeting control MO. **E**, The potential cross talk between *perp*, *pdcd2l*, and *p53* was studied by injecting zebrafish embryos (*flk1:EGFP-NLS* embryos) using MOs targeting *perp*, *pdcd2l*, and *p53* either singly (bars 3–5) or as double knockdowns (bars 6–8) or with a nontargeting control MO. **A**–**E**, EC apoptosis was studied in the presence (control MO) or in the absence (*sih* MO) of flow by whole-mount active caspase-3 staining at 30 hpf. The proportion of apoptotic ECs (number of apoptotic ECs divided by the total number of ECs) normalized to *sih* MO-injected embryos was calculated, and mean values are shown with SEM. n≥15 from 3 independent experiments. ns indicates nonsignificant. *P<0.05, ***P<0.001 using 1-way ANOVA.

To confirm the observed phenotypes, an alternative nonoverlapping MO was used for each gene (called MO2; Figure XA in the online-only Data Supplement). The efficiency of the MO2–mediated knockdown was confirmed by RT-PCR (*angptl4*, *cdh13*, and *pdcd2l*; Figure XB in the online-only Data Supplement), qRT-PCR (*angptl4*; Figure XC in the online-only Data Supplement) or by phenotypic analysis (*perp*; Figure XD in the online-only Data Supplement). Injection of the second, nonoverlapping *angptl4* MO2 resulted in 2.8-fold increase in EC apoptosis in the presence of flow (Figure 6A), whereas injection of *cdh13* MO2 led to ≈50% increase in EC apoptosis in the absence of flow (Figure 6B). On the contrary, injection of *pdcd2l* MO2 or *perp* MO2 resulted in an ≈40% decrease in EC apoptosis in the absence of flow (Figure 6C and 6D). Thus, the second MO recapitulated the effects of the initial MO for all 4 genes studied. Taken together, these results indicate that *cdh13* and *angptl4* play a protective role in the endothelium, whereas *perp* and *pdcd2l* promote EC apoptosis in response to static conditions.

### In Vivo Mechanistic Studies

To elucidate mechanisms that drive EC apoptosis in response to hemodynamic forces, we focused on 2 positive regulators of this process identified in our screening, *perp* and *pdcd2l.* To establish the mechanism by which they promote EC apoptosis, we returned to the porcine model and analyzed interrelations between apoptotic regulators identified by EC transcriptome profiling using Ingenuity Pathway Analysis. This assessment revealed p53 as a potential central regulator (Figure XI in the online-only Data Supplement). Therefore, we tested whether the p53 pathway is involved in flow-regulated EC apoptosis in zebrafish embryos. Knockdown of *p53* resulted in a decrease in EC apoptosis in the absence of flow that was comparable to *perp* or *pdcd2l* knockdown (Figure 6E; compare 3, 4, 5). To dissect the potential cross talk between *p53, perp*, and *pdcd2l*, we performed combined knockdowns. Coknockdowns of *perp* and *pdcd2l*, *p53*, and *perp* or *p53* and *pdcd2l* gave similar rates of apoptosis to single knockdown of each gene (Figure 6E; compare 6–8 with 3–5), suggesting that these molecules belong to a shared signaling pathway.

### Expression and Functional Studies in Mammalian ECs

Because screening models can generate false-positives, we attempted to confirm the expression and function of CDH13, PERP, ANGPTL4, PDCD2L in cultured EC exposed to flow and (where suitable antibodies were available) by en face staining of the aorta using mice.

#### Cadherin 13

**Figure 7.**
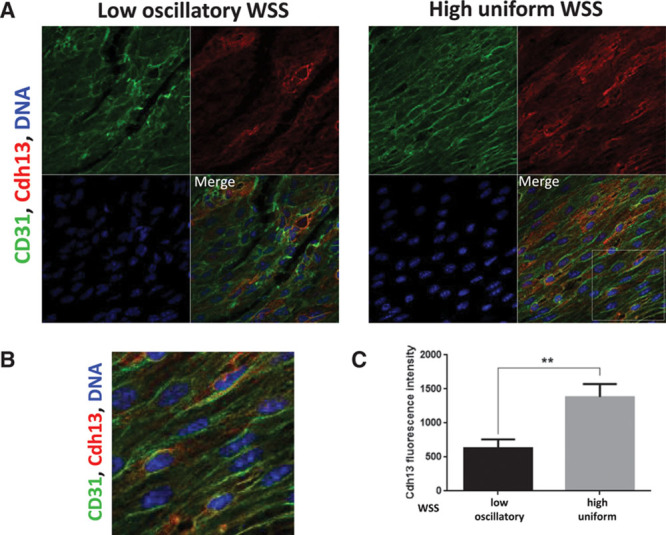
Cdh13 expression was enriched at a high shear stress region of the murine aorta. **A**, Expression levels of Cdh13 in endothelial cells (ECs) were assessed by en face staining of low oscillatory wall shear stress (WSS; inner curvature) or high WSS (outer curvature) of the aortic arch of C57BL/6 mice (red). ECs were identified by costaining with anti-CD31 antibodies conjugated to Alexa Fluor 488 (green). Cell nuclei were identified using To-Pro-3 (DNA, blue). The region outlined with the white box is shown in higher magnification in **B**. **C**, Graph showing quantitation of Cdh13 expression (mean fluorescence intensity with SEM). Data were pooled from 5 independent experiments. **P<0.01 using an unpaired 2-tailed *t* test.

En face staining of the murine aorta using anti-Cdh13 antibodies revealed that Cdh13 was expressed at higher levels in the high shear (outer curvature) compared with the low shear (inner curvature) region (Figure 7). A major portion of the Cdh13 pool localized to the plasma membrane (Figure 7B) suggesting that it is expressed in an active form in EC exposed to high shear. By contrast, control IgG did not produce a signal from murine arteries (Figure XII in the online-only Data Supplement). In addition, we compared gene expression in porcine aortic ECs exposed to flow patterns that model the in vivo situation (high uniform and low oscillatory WSS) using 2 complementary flow systems, an orbital shaker and an ibidi pump system. In the orbital shaking platform, the cells were exposed to flow using 6 well-tissue culture plates that were orbited to generate high unidirectional shear stress in the periphery of the well, and low shear with greater variation in direction at the center.^29^ On the contrary, the ibidi pump system involved seeding cells onto specialized μ-slides and the generation of WSS using a computer-controlled syringe pump to generate flow of desired magnitude and frequency. *CDH13* expression was enhanced in EC exposed to the high shear using the ibidi system but not in cells exposed to orbital flow (Figure XIII in the online-only Data Supplement) possibly reflecting differences in the mechanical conditions generated by these 2 systems.

**Figure 8.**
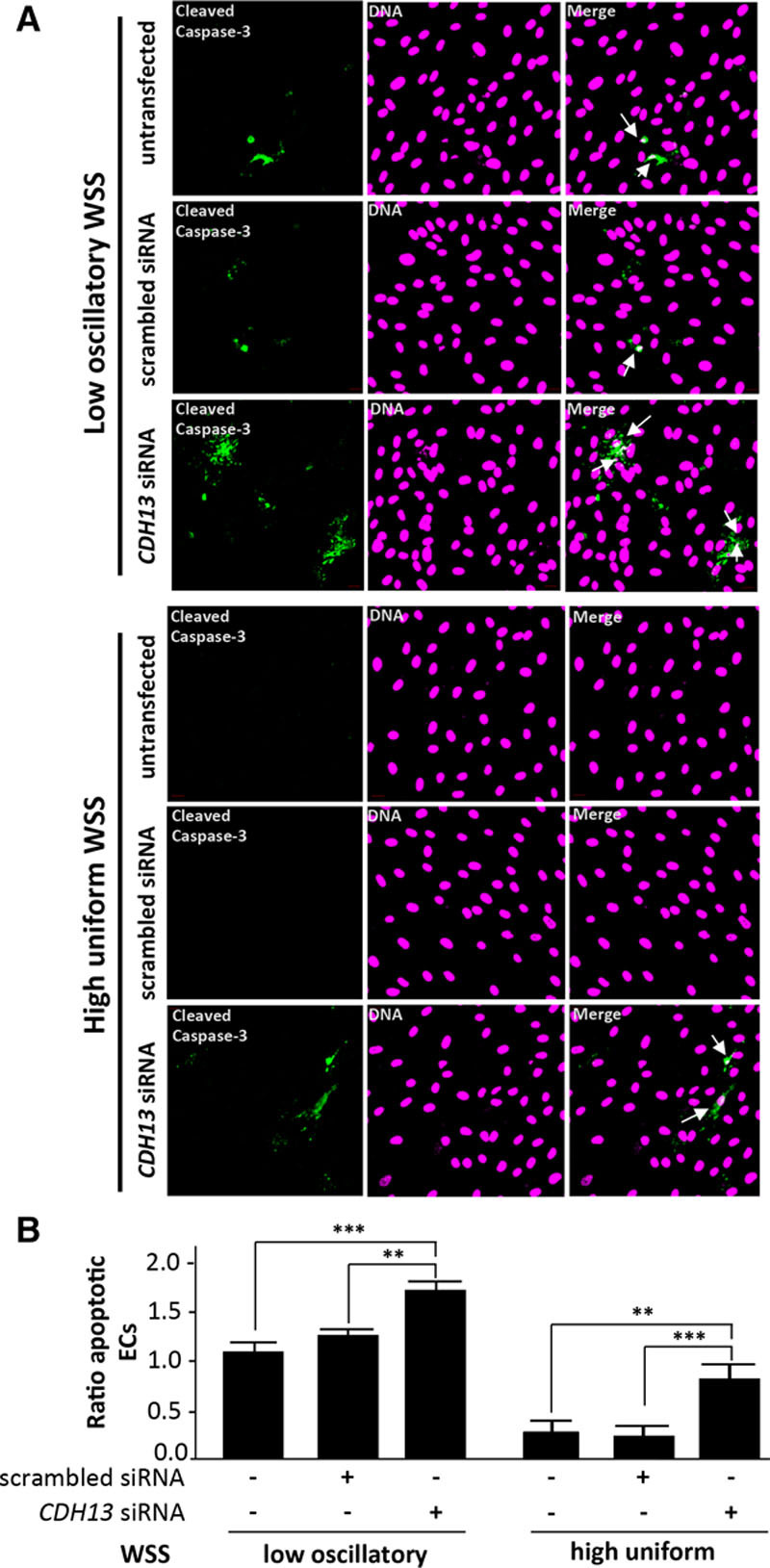
*CDH13* protected cultured endothelial cells (ECs) exposed to flow from apoptosis. **A** and **B**, Human umbilical vein endothelial cells (HUVECs) were transfected with scrambled sequences or *CDH13* siRNA or remained untransfected and incubated for 24 h. Cells were exposed for 72 h to low oscillatory (center) or high uniform (periphery) wall shear stress (WSS) using the orbital system. Apoptotic cells were measured by immunofluorescent staining using antibodies that detect cleaved caspase-3 (green) and counterstaining nuclei using To-Pro-3 (purple; DNA). Apoptotic ECs are indicated with white arrows. Data from at least 3 independent experiments were pooled, and the proportion of apoptotic cells are shown with SEM (**B**). ** *P*<0.01, ***P<0.001 by 2-way ANOVA.

We next wished to know whether CDH13 influences apoptosis in ECs cultured under flow. ECs exposed to low, oscillatory shear stress in vitro exhibited increased levels of EC apoptosis, as determined by active caspase-3 staining (Figure 8) and TUNEL assay (Figure XIV in the online-only Data Supplement). Notably, gene silencing in cultured human umbilical vein endothelial cells using small interfering RNA revealed that knockdown of *CDH13* (validated by qRT-PCR; Figure XV in the online-only Data Supplement) resulted in significantly increased rates of apoptosis in EC exposed to the high or low shear stress (Figure 8). Interestingly, although *CDH13* knockdown in zebrafish enhanced apoptosis under conditions of flow cessation, it did not influence EC exposed to flow, possibly because of compensatory mechanisms that protect sheared EC in developing fish. Nevertheless, the in vitro data broadly confirm those obtained by studying zebrafish embryos by indicating that CDH13 exerts antiapoptotic effects in EC.

#### p53-Related Protein

**Figure 9.**
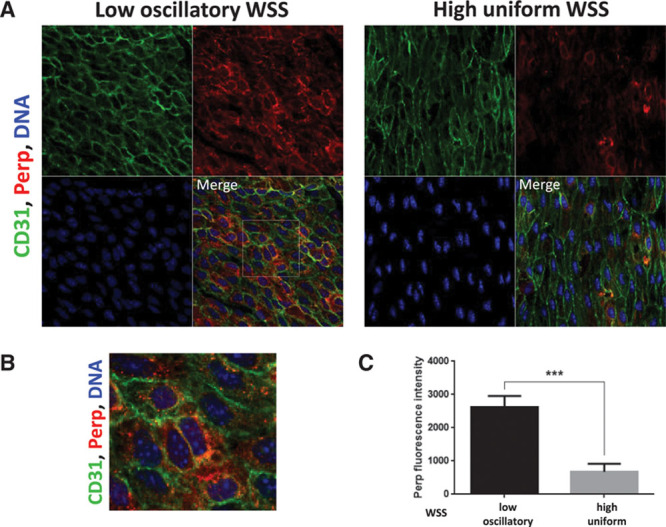
Perp expression was enriched at a low shear stress region of the murine aorta. **A**, Expression levels of Perp in endothelial cells (ECs) were assessed by en face staining of low oscillatory wall shear stress (WSS; inner curvature) or high WSS (outer curvature) of the aortic arch of C57BL/6 mice (red). ECs were identified by costaining with anti-CD31 antibodies conjugated to Alexa Fluor 488 (green). Cell nuclei were identified using To-Pro-3 (DNA, blue). The region outlined with the white box is shown in higher magnification in **B**. **C**, Graph showing quantitation of Perp expression (mean fluorescence intensity with SEM). Data were pooled from 5 independent experiments. ***P<0.01 using an unpaired 2-tailed *t* test.

**Figure 10.**
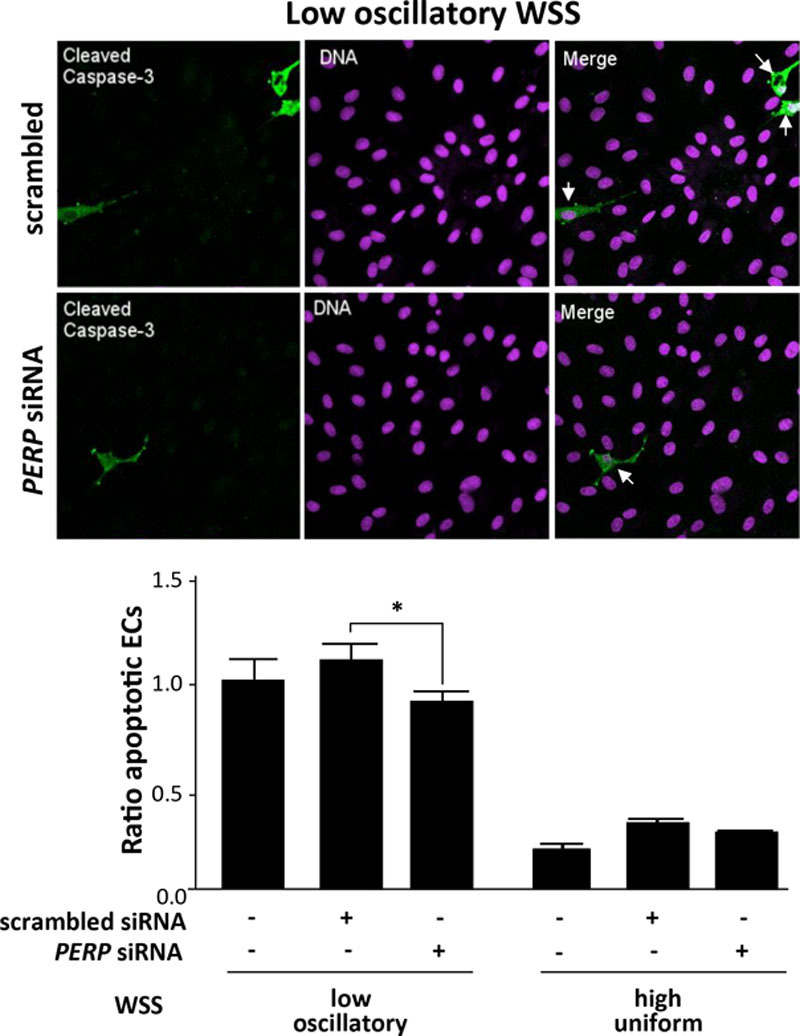
*PERP* promoted apoptosis of cultured endothelial cell (EC) exposed to low wall shear stress (WSS). Human umbilical vein endothelial cells (HUVECs) were transfected with scrambled sequences or *PERP* siRNA or remained untransfected and incubated for 24 h. Cells were exposed for 72 h to low oscillatory (center) or high uniform (periphery) WSS using the orbital system. Apoptotic cells were measured by immunofluorescent staining using antibodies that detect cleaved caspase-3 (green) and counterstaining nuclei using To-Pro-3 (purple; DNA). Apoptotic ECs are indicated with white arrows. Data from at least 3 independent experiments were pooled, and the proportion of apoptotic cells are shown with SEM. *P<0.05, by 2-way ANOVA.

En face staining of the murine aorta revealed that Perp was expressed at higher levels in the low shear (inner curvature) compared with the high shear (outer curvature) region (Figure 9). Perp localized predominantly to the plasma membrane that is consistent with its localization in other tissues (Figure 9B).^30^ Similarly, the expression of PERP was elevated in cultured porcine aortic ECs exposed to the low shear compared with cells exposed to the high shear using either the ibidi parallel-plate system or an orbital platform (Figure XIII in the online-only Data Supplement). Knockdown of *PERP* (validated by qRT-PCR; Figure XV in the online-only Data Supplement) resulted in significant reduction in EC apoptosis (Figure 10). These data confirm those generated by screening genes in zebrafish embryos and indicate that PERP is expressed under the low shear conditions where it promotes EC apoptosis in both in vitro and in vivo systems.

#### Angiopoietin-Like 4 and Programmed Cell Death 2–Like Protein

Studies of cultured porcine aortic ECs revealed that *ANGPTL4* and *PDCD2L* were expressed at higher levels in cells exposed to the low compared with high shear stress using the orbital system (Figure XIII in the online-only Data Supplement), which is consistent with their enrichment at the low shear region of arteries (Figure 3). However, *ANGPTL4* was not enriched under the low shear stress using the ibidi platform possibly reflecting differences in the mechanical environments generated by the orbital and parallel-plate systems. Silencing of *ANGPTL4* or *PDCD2L* using small interfering RNA (validated by qRT-PCR; Figure XV in the online-only Data Supplement) did not influence apoptosis in cultured EC exposed to the high or low WSS (Figure XVI in the online-only Data Supplement). Thus, although both of these molecules regulated apoptosis in zebrafish embryonic EC exposed to flow, they did not influence the viability of cultured mammalian EC, possibly reflecting differences in the developmental stage, mechanical environment, or species studied.

Taken together, our studies of murine arteries and cultured EC exposed to different shear stresses confirm observations made using zebrafish embryos that PERP and CDH13 function as shear-responsive regulators of endothelial apoptosis. By contrast, the effects of ANGPTL4 and PDCD2L on EC apoptosis observed in zebrafish embryos were not recapitulated in vitro. The study demonstrates the utility of our zebrafish platform for functional screening of shear-responsive genes and also emphasizes the potential divergent functions of some genes in in vivo and in vitro systems.

## Discussion

The molecular mechanisms underlying the effects of WSS on EC physiology and atherosclerosis are not fully understood but are known to involve transcriptional changes. Although transcriptome analysis of EC exposed to different flow parameters under in vitro or in vivo conditions^16–19,31,32^ has identified multiple mechanoresponsive genes, the majority of them have not been studied at a functional level. To address this challenge, we established a zebrafish platform to allow functional screening of flow-responsive genes. To our knowledge, this is the first in vivo system developed for screening of mechanosensitive genes.

The zebrafish embryo model that we used diverges from vascular response to flow in mammalian arteries in several important respects including species, scale, and hemodynamics. Nevertheless, there are many parallels between endothelium exposed to static conditions in zebrafish, on the one hand, and endothelium exposed to disturbed flow and low, oscillatory WSS in mammals, on the other. For example, the atheroprotective transcription factor *KLF2*/*klf2a* was suppressed by static conditions in embryos and by low WSS conditions in adult vertebrates.^33^ In addition, EC cilia are present mainly in areas of low WSS in mammalian arteries and in static conditions in zebrafish embryos and were shown to disassemble on exposure to laminar shear stress in both systems.^34–36^ Finally, a recent study found considerable overlap in the transcriptome of EC exposed to low shear or static conditions.^37^ Because of these considerations, we hypothesized that manipulation of genes in developing zebrafish vessels may provide information on the pathways that regulate mammalian arterial physiology. Consistent with this notion, our study revealed that flow regulates EC apoptosis via mechanisms that are, at least in part, conserved between embryonic and adult arteries.

We assessed the ability of the zebrafish model to identify mechanosensitive genes that control EC function by focusing on apoptosis regulators that are controlled by flow. To generate a panel of putative flow-sensitive apoptosis genes, we performed microarray transcriptome analysis of the high and low shear stress regions of the porcine aorta. Functional annotation revealed apoptosis regulators as the most highly enriched group, which is consistent with the hypothesis that multiple genes regulate EC apoptosis at regions of disturbed flow. Our data build on pioneering studies from Peter Davies’ group revealing enrichment of proinflammatory and stress response molecules at the inner curvature of the porcine aorta.^10,16,38^ Notably, our study revealed multiple WSS-related genes that were not identified as differentially expressed previously. This discrepancy may be related to technological differences because the commercial porcine gene arrays used in the current study contained >10 000 genes more than the custom arrays used previously,^10,16^ and analysis of our data benefitted from sequencing and annotation of the porcine genome (http://www.ensembl.org/Sus_scrofa/Info/Index). More fundamentally, there is only a partial overlap between the anatomic sites studies by the Davies group and those studied currently. For example, whereas we used outer curvature of the aortic arch as the source of ECs exposed to atheroprotective flow, previous studies focused on the descending thoracic aorta,^16^ carotid artery, and renal artery.^10,16^ Of note, although only a proportion of genes were found to be consistently differentially expressed in the current and previous studies of porcine arteries, the majority of them showed a similar pattern of expression in the low and high shear areas (Table IV in the online-only Data Supplement). It is also interesting to compare the current study with elegant experiments that revealed a causal relationship between WSS and the EC transcriptome in the murine carotid artery.^17^ Here, WSS was modified in the left carotid artery by partial ligation before assessment of EC gene expression. This approach revealed that WSS regulates multiple genes in murine ECs including those involved in inflammation and immunity. Interestingly, although our current study of the porcine aorta identified several genes that were also regulated by WSS in the murine carotid artery,^17^ the relationship with WSS was not conserved between models (Table IV in the online-only Data Supplement). Several important biological differences can potentially explain this disparity including differences in vascular bed, variation in fluid dynamics, species, and the time that EC were exposed to disturbed flow. We also compared our microarray data to the list of >1600 shear-responsive genes obtained by meta-analysis of published microarray studies performed in vitro on human umbilical vein endothelial cells (Table V in the online-only Data Supplement).^39^ We found that <50% of genes shown to be mechanically regulated in both data sets exhibit the same relationship with WSS. This observation further emphasizes the sensitivity of EC to physiological and mechanical stimuli that vary between in vitro and in vivo systems.

After our EC transcriptome analysis, we selected the putative apoptosis regulators that displayed the greatest differential expression for functional screening in zebrafish. Comparison between human and zebrafish reference genomes shows that ≈70% of human genes have at least 1 obvious zebrafish orthologue.^40^ Therefore, not all human genes can be studied in zebrafish, which was the case for *CCL2* and *CSF2*. In addition, because of a whole-genome duplication that occurred early during the evolution of ray-finned fishes, many human genes have >1 orthologue in the zebrafish genome. Because functional redundancy between zebrafish paralogues can present a challenge when studying zebrafish physiological processes, we excluded from our study genes with >2 orthologues. Gene function in zebrafish has been studied using transient knockdown via MOs or by targeted gene disruption and generation of mutants. For functional screening of a large number of genes, generation of mutants is costly, labor intensive, and time consuming (zebrafish and mice have similar generation times). Therefore, our approach was to use MOs for the functional screening. One drawback of using MOs is the lack of spatiotemporal control because they are injected into embryos at the 1-cell stage and therefore reduce expression in all embryonic cells. Because of this, genes important for early embryonic development cannot be studied using this model. This was the case for 3 genes in our study, *fadd*, *igf1*, and *tnfsf10*, where knockdown caused gross morphological defects. Nevertheless, we were able to study vascular responses to flow after knockdown of 75% genes where embryonic morphology was normal. Overall, of 12 genes studied, we detected 2 antiapoptotic (*angptl4* and *cdh13*) and 2 proapoptotic molecules (*pdcd2l* and *perp*) that were enriched at mechanically distinct regions of the aorta. To confirm the specificity of the MO-mediated knockdown phenotype, we used 2 separate, nonoverlapping MOs for each gene of interest.

To confirm our observations from functional screening in zebrafish embryos, we performed a series of experiments using cultured EC exposed to flow. First, to understand their mechanism of differential expression, we examined whether *CDH13*, *PERP*, *PDCD2L*, and *ANGPTL4* respond to WSS in cultured ECs. We used 2 complementary platforms, an orbital shaker and an ibidi pump system. In the orbital shaker system, the cells in the center of the well are exposed to WSS with a constant low mean magnitude and rapid changes in direction, whereas in the periphery of the well the WSS magnitude is relatively high and with relatively uniform flow direction.^29^ This contrasts with the ibidi parallel-plate system that uses a syringe pump to generate high uniform and low oscillatory (1 Hz bidirectional) WSS. Two genes, *PERP* and *PDCD2L*, were expressed at higher levels in EC exposed to low WSS conditions in both in vitro systems, suggesting that their focal expression in vivo is maintained by local hemodynamics. On the other hand, *CDH13* expression was enriched by high WSS only in the ibidi system, whereas *ANGPTL4* upregulation under low WSS was only observed in the orbital system. These observations may reflect differences in the mechanical environment of the orbital and ibidi systems, for example, the presence of secondary flows that are generated exclusively by the orbital platform,^29^ and they emphasize the importance of studying mechanosensitive gene function using multiple experimental models. Second, we assessed the effects of silencing our genes of interest in cultured EC exposed to flow. This study confirmed that *PERP* is a positive regulator of EC apoptosis under low WSS conditions and that *CDH13* has protective effects (shown schematically in Figure XVII in the online-only Data Supplement). Interestingly, although *CDH13* reduced apoptosis in cultured EC exposed to low or high WSS, its protective effects in zebrafish embryos were only observed in EC exposed to static conditions. This discrepancy may arise from compensatory mechanisms present in embryonic but not in adult vessels exposed to flow. Further studies including genetic deletion of Cdh13 in mice are therefore required to determine whether CDH13 exerts protective effects under low and high WSS conditions in adult mammalian arteries. Our study also indicated that although *angptl4* and *pdcd2l* regulate apoptosis in zebrafish endothelium, they did not influence EC viability in cultured human cells exposed to low or high WSS. Therefore, these molecules may regulate apoptosis specifically in developing EC, or their function may require anatomic or physiological features that are found in vivo but not in cultured cells, or there may be species-based differences. Further work is required to distinguish between these possibilities. Although we provide the first demonstration that *perp, cdh13*, *angptl4*, and *pdcd2l* are involved in hemodynamic control of apoptosis, they have been linked previously with apoptosis in other systems. PERP was previously identified as a p53 target gene by subtractive cloning of mouse embryonic fibroblasts and was shown to be expressed exclusively in apoptotic cells.^30^ Interestingly, PERP can feed back to p53 to promote its activity^41^ which is consistent with our observation that PERP and p53 cooperate to promote apoptosis in ECs. Our findings also resonate with previous studies that revealed post-translational modifications of p53 playing a role in disturbed flow-mediated EC apoptosis and contributing to atherosclerotic plaque formation.^13,15^ CDH13 has been shown to protect ECs from oxidative stress–induced apoptosis,^42^ and ANGPTL4 acts as a survival factor in the endothelium.^43^ Finally, although little is known about the function of PDCD2L, its paralogue PDCD2 promotes apoptosis in many human and mammalian cell lines and tissues.^44^

Although zebrafish embryos recapitulate some of the pathways that are regulated by flow in adult arteries, there are also limitations. One notable example is the hypoxia-inducible factor 1α pathway that was enriched at the inner curvature of the porcine aorta (Figure 3) but was not activated in zebrafish embryos under static conditions (Figure I in the online-only Data Supplement). This discrepancy may be attributable to differences in oxygen transport and inflammation between these 2 systems. In the porcine aorta, oxygen delivery to luminal EC relies on transport of red blood cells through the vessel lumen. Computational modeling and empirical measurements suggest that this process can be influenced by secondary flows, which convect oxygen away from the vessel wall to generate regions with reduced oxygen levels.^45,46^ By contrast, oxygenation of zebrafish embryos (up to 72 hpf) does not rely on circulation because diffusion is sufficient because of their small size.^22–24^ Thus, although sites of disturbed flow in the aorta may have lower oxygen tensions leading to enhanced hypoxia-inducible factor 1α activation, this pathway is not activated in zebrafish in response to flow cessation because diffusion prevents the development of a hypoxic environment. Moreover, hypoxia-inducible factor 1α can also be induced by inflammatory signaling pathways^47^ that are known to be activated constitutively at the inner curvature of the aortic arch^16,47^ but have an uncertain role in zebrafish embryo responses to flow. Thus, we conclude that although zebrafish embryos provide a useful in vivo model to assess vascular responses to WSS, other systems are required to study the effects of altered oxygen transport on the vasculature.

In summary, we have established a zebrafish-based model for functional screening of flow-sensitive genes and have used this system to identify novel regulators of EC apoptosis in response to disturbed flow. The study provides an additional mechanism to explain the focal distribution of EC injury and dysfunction in arteries and suggests that this involves p53-PERP–mediated EC apoptosis at atheroprone sites and CDH13-mediated EC survival at atheroprotected sites.

## Sources of Funding

This study was supported by the British Heart Foundation (Programme Grant RG/13/1/30042).

## Disclosures

None.

## Supplementary Material

## Highlights

- Atherosclerosis develops at branches and bends of arteries exposed to disturbed blood flow.
- We investigated whether functional screening of flow-sensitive genes can be achieved using zebrafish, a vertebrate model that can be genetically modified with ease.
- Manipulation of flow altered endothelial cell apoptosis in developing zebrafish vessels, and this process was modified by the knockdown of a panel of flow-sensitive genes.
- We conclude that a zebrafish model of blood flow manipulation coupled to gene knockdown can be used for functional screening of mechanosensitive genes.
