# Supplementary material for: Zebrafish Model for Functional Screening of Flow-Responsive Genes
Source: Arterioscler Thromb Vasc Biol. 2016 Dec 21;37(1):130–43. doi: 10.1161/ATVBAHA.116.308502 (PMC5172514; doi:10.1161/ATVBAHA.116.308502)
Supplement: Supplementary file 1 [file atv-37-130-s001.pdf]

SUPPLEMENTAL MATERIAL

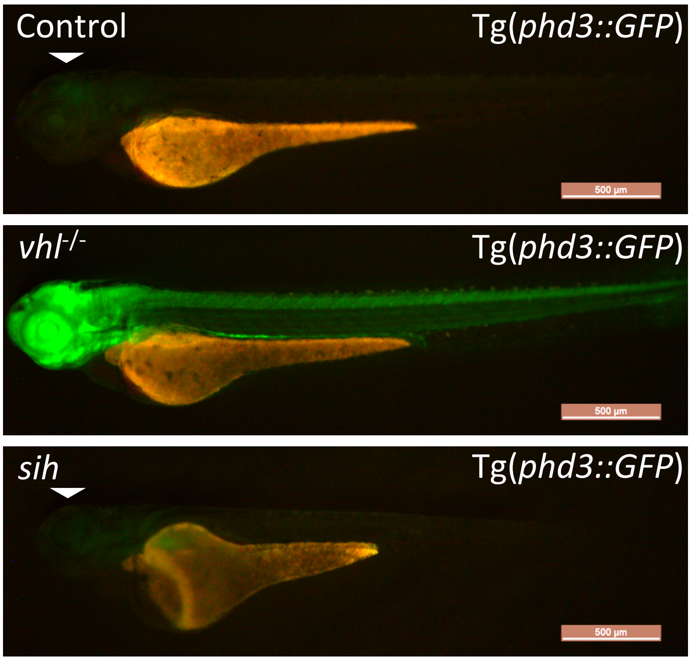

Supplemental Fig. I

***Silent heart* embryos do not exhibit hypoxia.** The hypoxic response was monitored in transgenic embryos containing a hypoxia reporter Tg(*phd3::GFP*). Embryos deficient in von Hippel-Lindau (*vhl*) tumour suppressor gene expression were used as a positive control. *Vhl*$^{-/-}$ embryos exhibit a systemic hypoxic response, reflected by upregulation of GFP expression. No upregulation of GFP expression was observed in *sih* embryos lacking blood flow compared to controls. White arrowheads indicate mild GFP fluorescence in the brain region. Lateral view of 3 day old embryos.

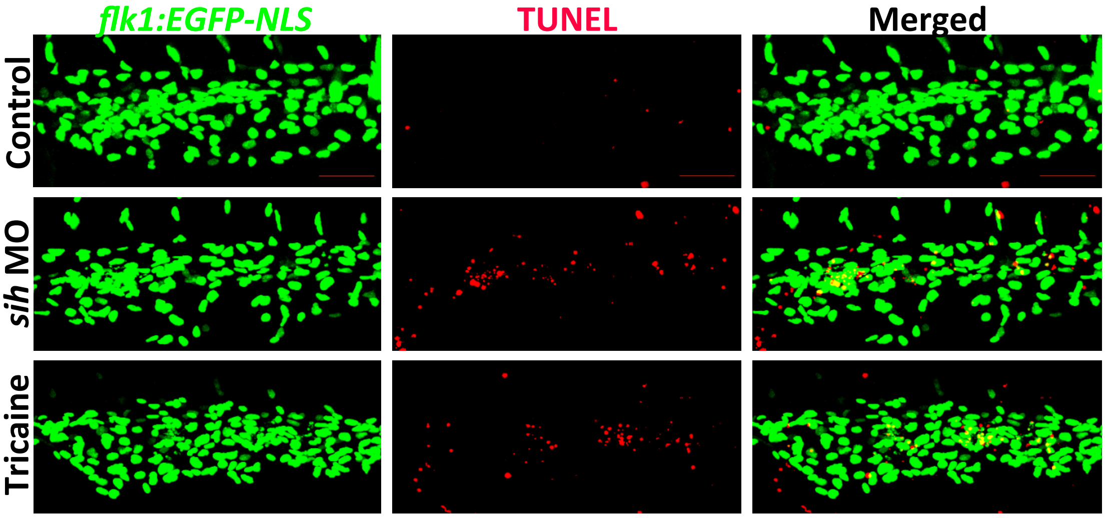

Supplemental Fig. II

Flow cessation induces EC DNA fragmentation in zebrafish embryos at 30 hpf. Whole-mount TUNEL staining (red) of 30 hpf *flk1:EGFP-NLS* embryos (green EC nuclei) in the presence (control) or absence of flow (*sih* MO, tricaine). Lateral view, anterior to the left, dorsal up. Scale bar: 50 µm.

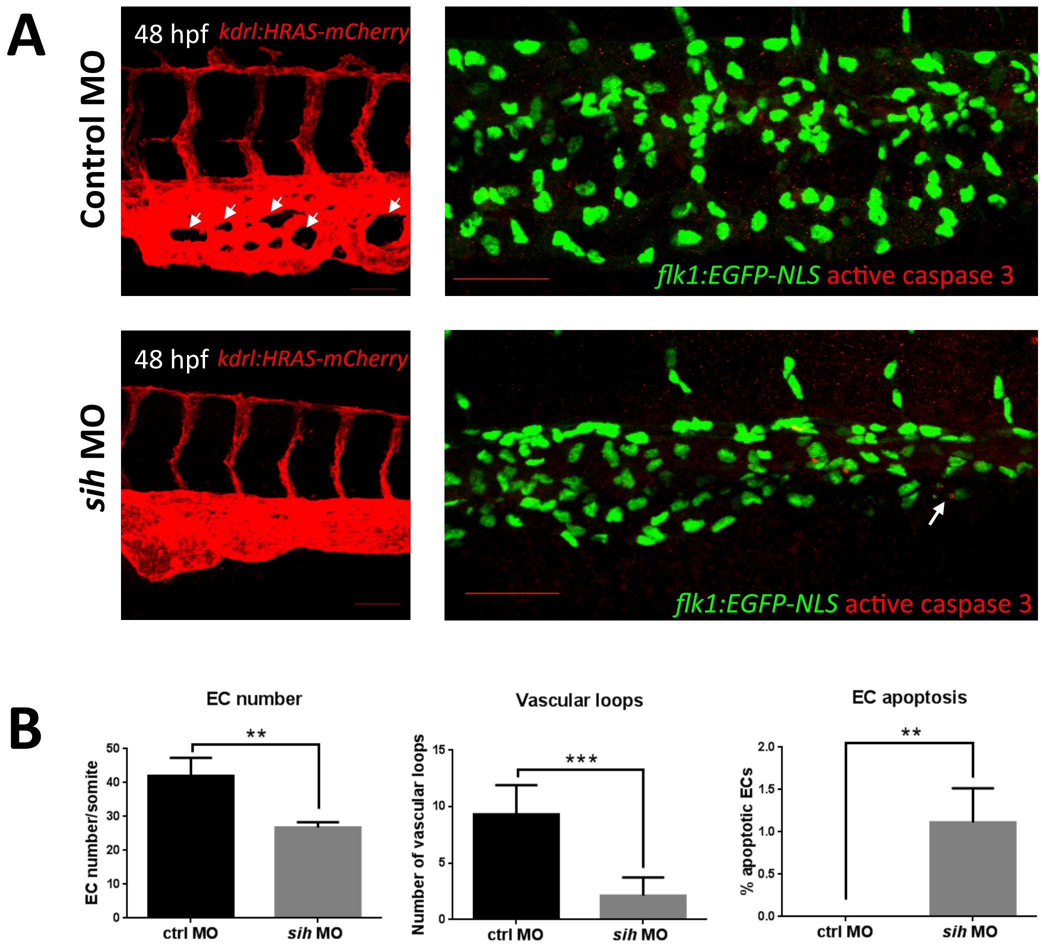

Supplemental Fig III

Flow cessation reduces the complexity of the caudal plexus in zebrafish embryos at 48 hpf. Whole-mount active caspase-3 staining (red) of 48 hpf *flk1:EGFP-NLS* embryos (green EC nuclei) and assessment of vascular morphology of 48 hpf *kdrl:HRAS-mCherry* (red EC) embryos in the presence (control) or absence of flow (*sih* MO). (A) Representative images are shown. Caudal vein plexus morphology was less complex and with fewer loops (indicated with white arrows) in the absence of flow (*sih* MO) compared to controls (left panels). A modest increase in apoptotic EC was observed in *sih* MO compared to controls (right panels). (B) EC numbers, number of vascular loops and percentage of apoptotic ECs were quantified in *sih* embryos and controls at 48 hpf and are presented as mean values with standard deviation. n≥15 from three independent experiments; **$p<0.01$, ***$p<0.001$ using an unpaired two-tailed t-test.

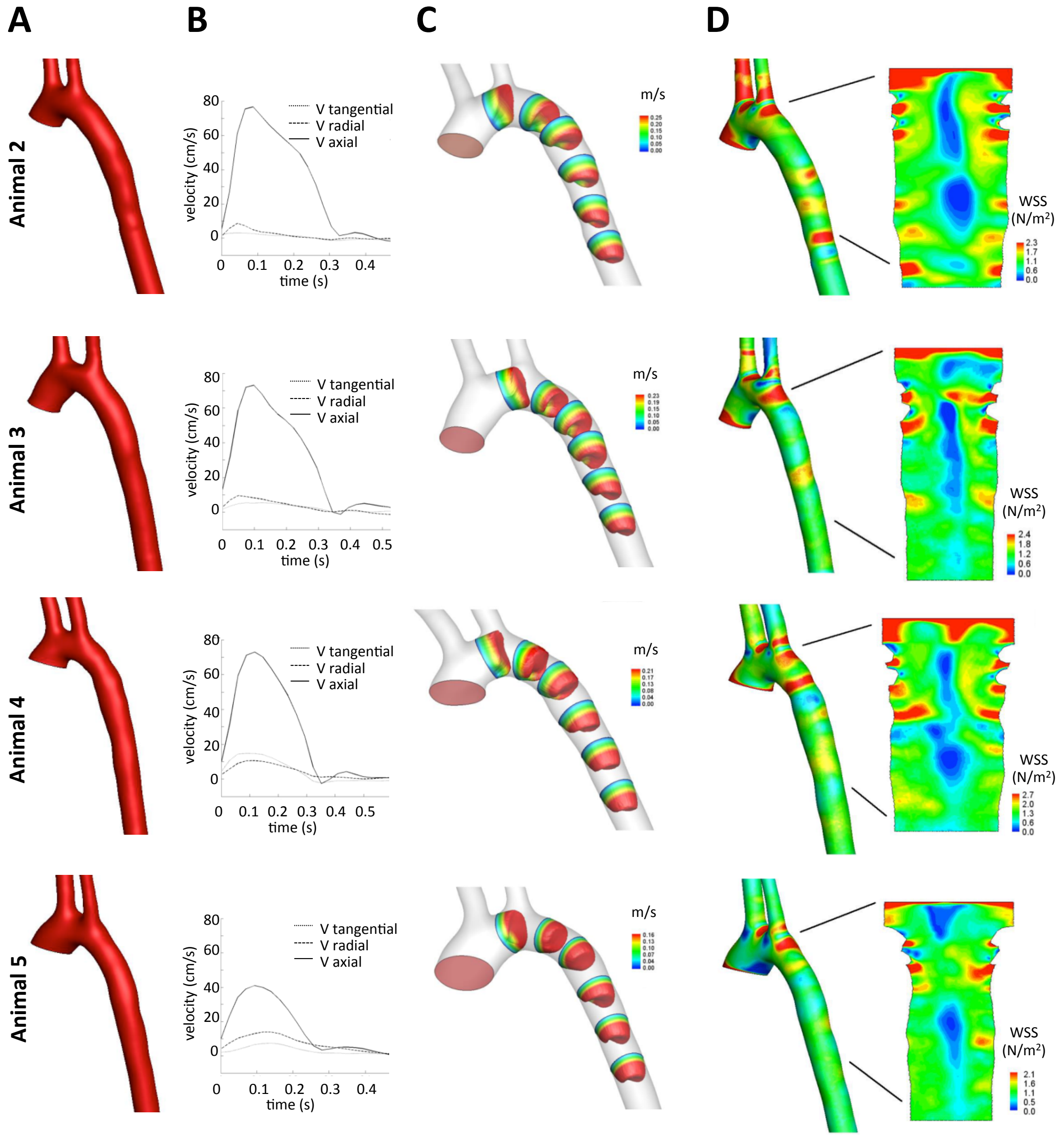

Supplemental Fig IV

Steady state fluid dynamics in the porcine aorta: variation between animals. Steady state fluid dynamics were studied in the aortae of pigs using MR imaging and CFD. (A) The surfaces of the aortic arch were reconstructed from MR images, smoothed with a low-pass filter and extended with cylindrical flow extensions at the outlets. (B) Velocity waveforms were acquired at the inlet section of the ascending aorta by phase contrast-MRI and post-processed in Matlab. The three components of the velocity in the local coordinate system are shown. (C) Three-dimensional velocity contours are shown over six representative planes. (D) A time-averaged WSS map was calculated and mapped onto the aortic geometry (left panel). The time-averaged WSS map was unwrapped via a computational incision over the outer aortic wall and the 2-dimensional WSS map was visualized with the endothelial layer facing upwards (right panel). High WSS is represented in red and low WSS in blue.

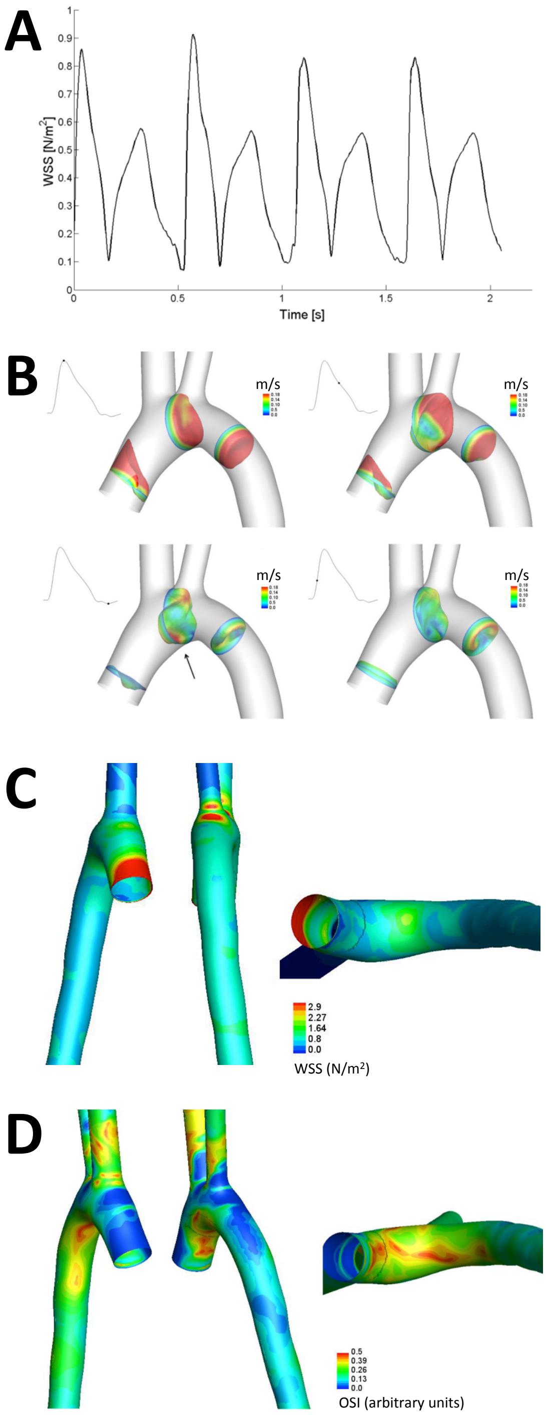

Supplemental Fig V. Unsteady fluid dynamics in the porcine aorta. Fluid dynamics in the porcine aorta were studied over the cardiac cycle using MR imaging and CFD. (A) Instantaneous WSS magnitudes were calculated for a single point on the inner curvature of the vessel during the first four cardiac cycles of simulated pulsatile flow. (B) Velocity profiles are shown for four representative points of the cardiac cycle for a porcine aortic arch. The arrow indicates peak retrograde flow in diastole at the inner curvature of the arch. (C) Time-averaged WSS (TAWSS) distribution at the porcine aortic arch, depicted at three different views. High TAWSS is represented in red and low TAWSS in blue. (D) Oscillatory Shear Index (OSI) distribution at the porcine aortic arch, depicted at three different views. Maximum OSI is highlighted in red and represents a 180° deflection of the WSS direction.

A

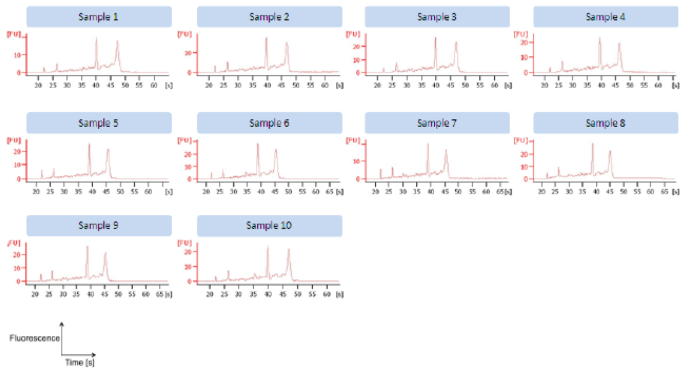

B

Subject	Condition	$A_{260/280}$	Concentration [ng/µl]	18s/28s	RIN
Pig 1	High shear stress	1.98	20.7	1.5	7.4
Pig 1	Low shear stress	1.9	21.7	1.4	7.2
Pig 2	High shear stress	2.03	29.4	1.4	7.3
Pig 2	Low shear stress	1.88	22.3	1.2	7.5
Pig 3	High shear stress	2.01	24.4	1.4	7.4
Pig 3	Low shear stress	2.17	39.1	1.4	7.4
Pig 4	High shear stress	1.92	24.9	1.4	7.4
Pig 4	Low shear stress	1.94	40.6	1.4	7
Pig 5	High shear stress	1.96	19.6	1.5	7.3
Pig 5	Low shear stress	1.96	19.6	1.5	7.3

C

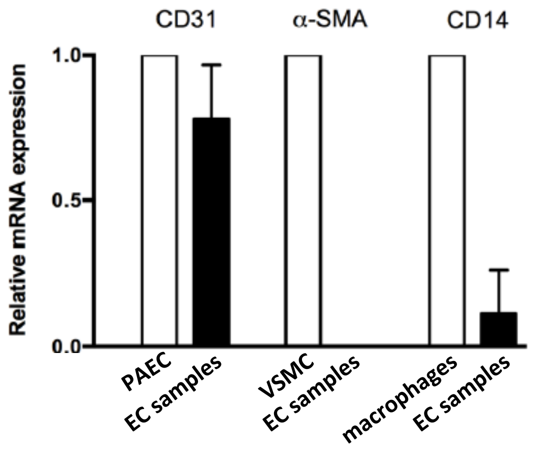

Supplemental Fig VI. Quality control measures for RNA sampled from porcine aortic endothelium. RNA was extracted from ECs at high and low WSS regions in 5 pigs. The purity and integrity of total RNA samples was assessed using a Bioanalyser (Agilent). (A) RNA electropherograms are presented for each sample. (B) Summary of the absorbance ratio 260 nm/ 280 nm (A260/280; a measure of RNA purity), concentration, ratio of 18s/28s ribosomal RNA levels and RNA integrity number (RIN) for each sample. (C) The purity of EC samples was assessed by qPCR analysis of an EC marker (CD31), a vascular smooth muscle cell (VSMC) marker (a-SMA) and a macrophage marker (CD14). Cultured PAECs, VSMCs and macrophages were tested as positive controls for CD31, a-SMA and CD14 expression respectively.

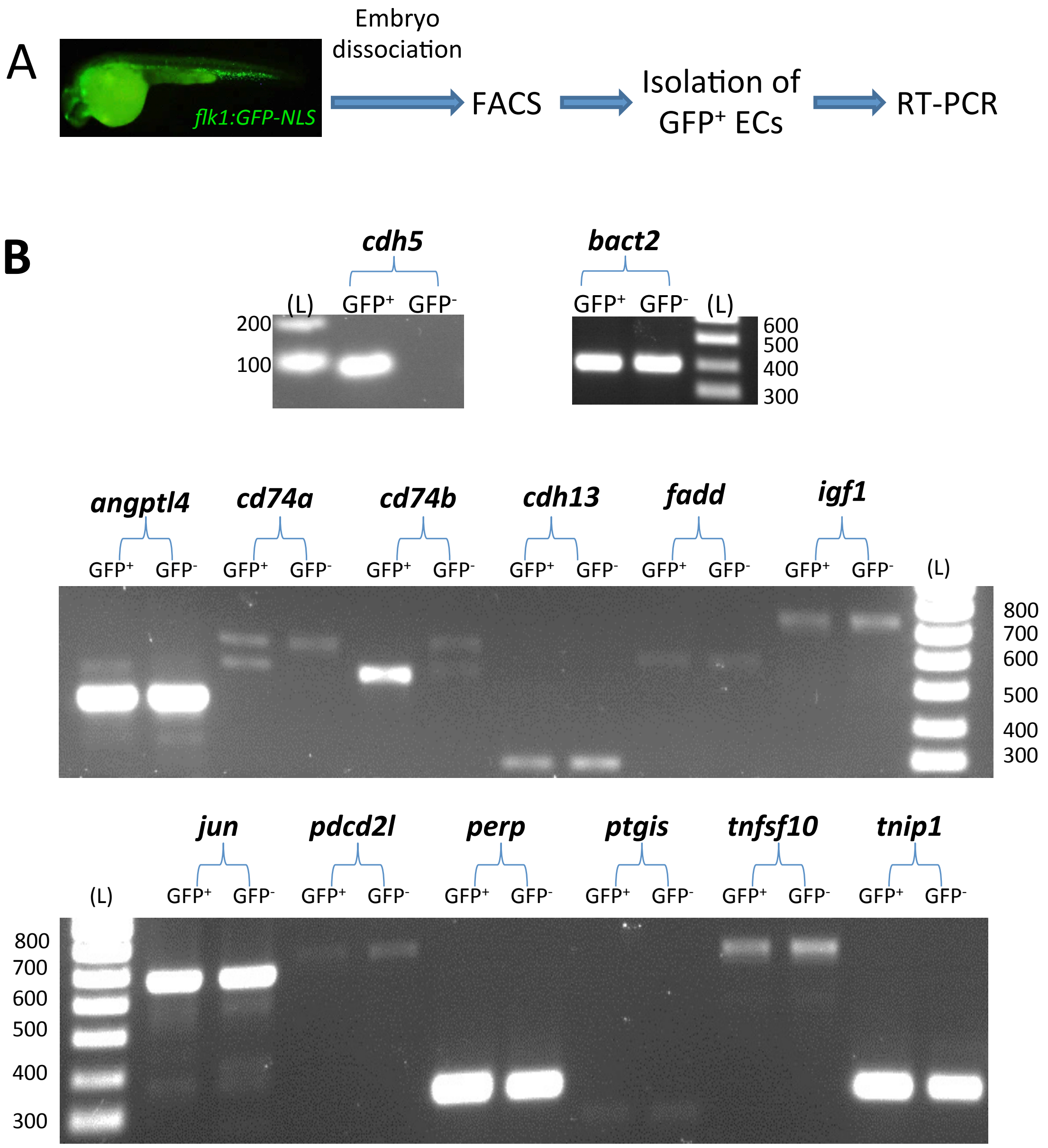

Supplemental Fig VII. Isolation of ECs from zebrafish embryos and measurement of candidate gene expression by RT-PCR. (A) ECs were isolated from 26 hpf *flk1:EGFP-NLS* zebrafish embryos using fluorescence-activated cell sorting (FACS). (B) Expression of EC marker *cdh5*, house-keeping gene beta-actin (*bact2*) and candidate genes of interest was assessed in ECs (GFP$^+$) by RT-PCR followed by analysis of product by gel electrophoresis, while GFP$^-$ cells were used as a control. The DNA fragment sizes are indicated in base pairs next to the DNA ladder (L).

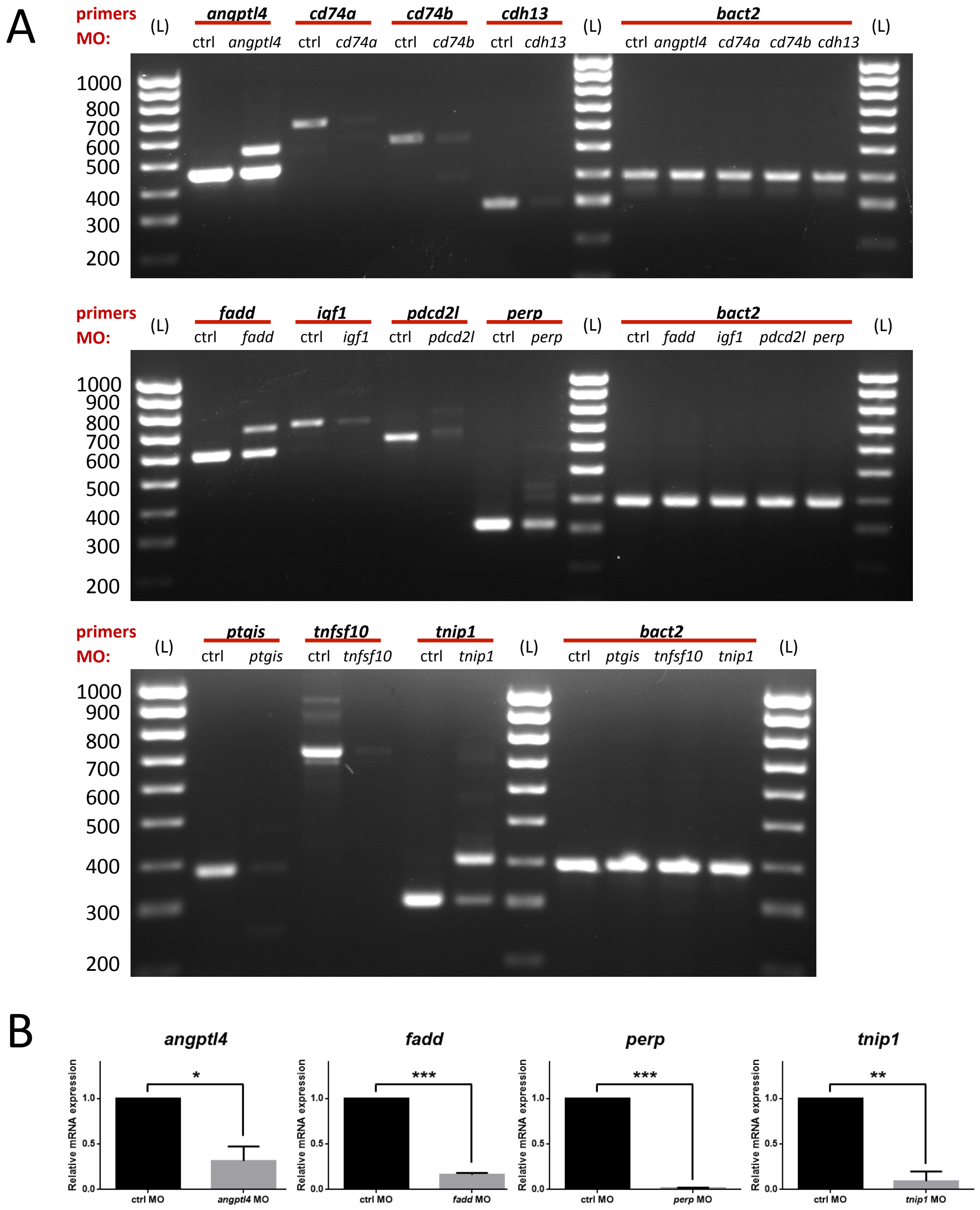

Supplemental Fig VIII

Validation of MO efficiency. (A) The ability of gene-specific splice-blocking MOs to modify splicing of targeted transcripts was assessed by analysis of RT-PCR products after agarose gel electrophoresis. Splicing modifications were observed as a band shift or as a reduction of the wildtype band compared to the control sample (ctrl MO). RT-PCR analysis of beta-actin (*bact2*) was used as an internal control. The DNA fragment sizes are indicated in base pairs next to the DNA ladder (L). (B) Embryos with residual expression of the wildtype transcript (i.e. *angptl4*, *fadd*, *perp* and *tnip1* in (A)) were additionally tested by qPCR to validate MO efficiency. Based on sequencing results of the additional bands produced by MO injection (data not shown), the qPCR primers were designed to detect the wildtype transcript only. Mean values are shown with standard error of the mean. n=3 from three independent experiments; *p<0.05, **p<0.01, ***p<0.001 using an unpaired two-tailed t-test.

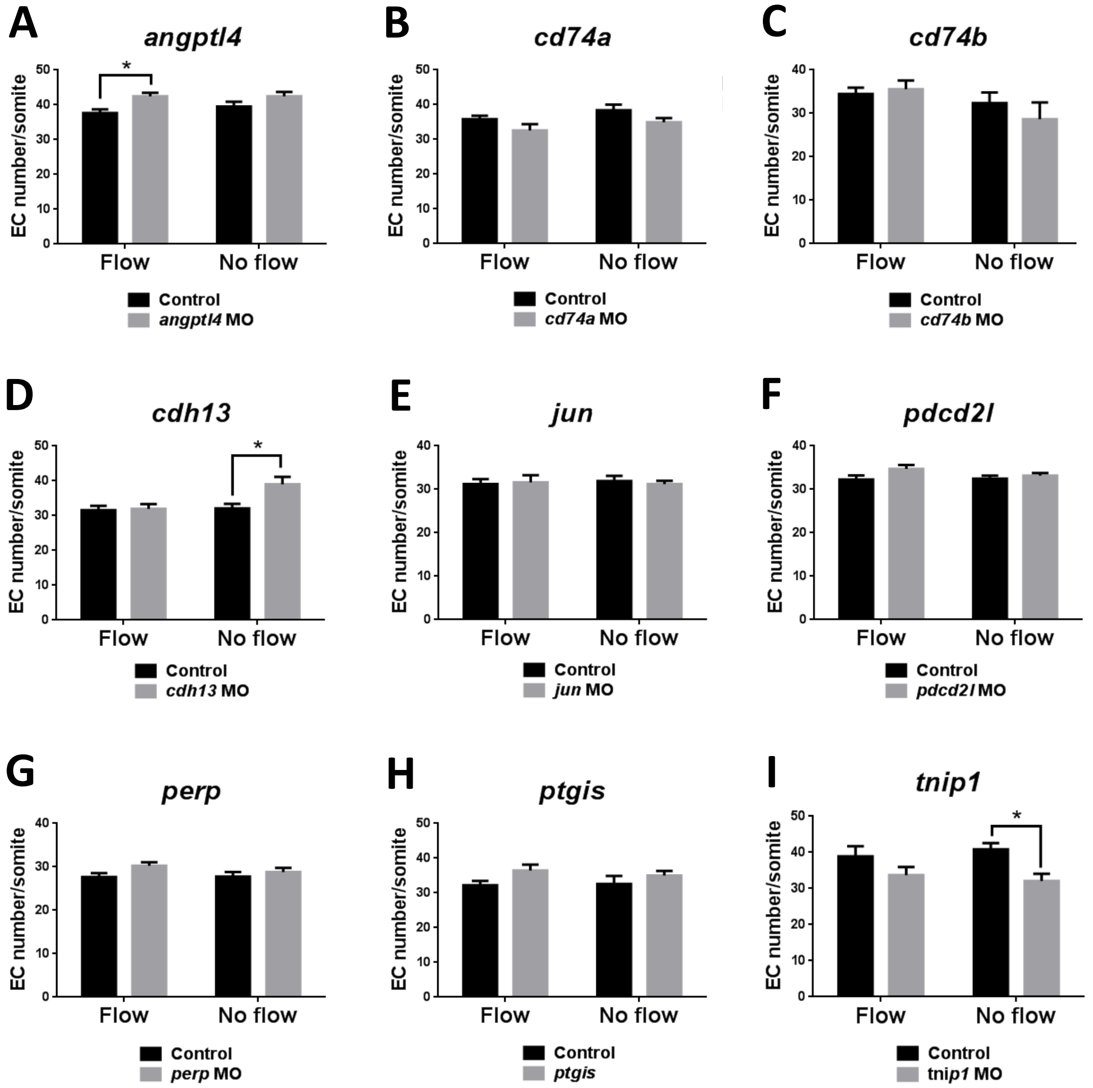

Supplemental Fig IX.

Quantification of EC numbers in knockdown embryos. (A-I) Zebrafish *flk1:EGFP-NLS* embryos were injected with MOs targeting candidate genes or a non-targeting control MO in the presence or in the absence of flow (*sih* MO). Graphs show EC numbers (number of GFP^+ EC nuclei) normalised to *sih* MO-injected embryos. Mean values are shown with standard error of the mean. n≥15 from three independent experiments. $^*p<0.05$, using one-way ANOVA.

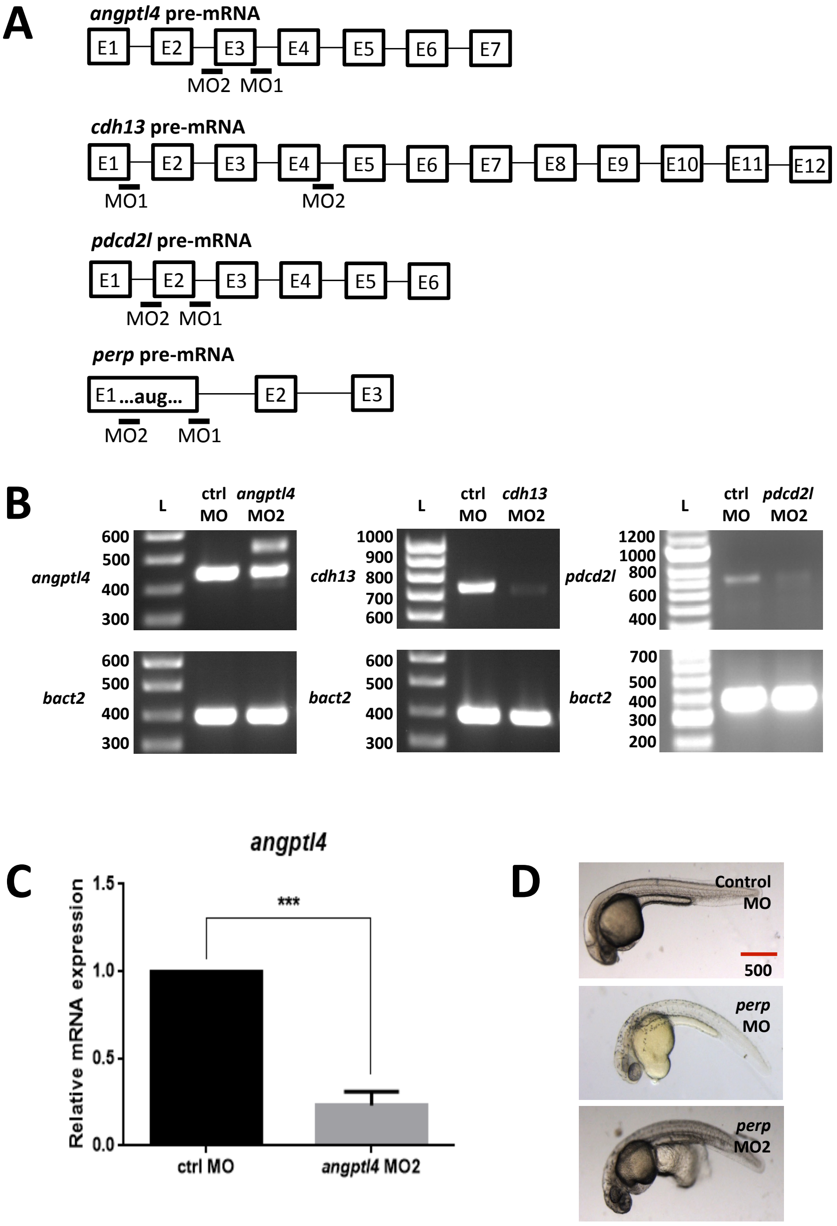

Supplemental Fig X. Design and validation of second non-overlapping MOs to target *angptl4*, *cdh13*, *pdcd2l* and *perp*. (A) Diagramatic representation of *angptl4*, *cdh13*, *pdcd2l* and *perp* pre-mRNAs with exons (numbered boxes) and introns (solid lines). Exon and intron sizes are arbitrary. The regions in pre-mRNA where MOs bind are indicated with black bars labelled MO1 or MO2. (B) Zebrafish embryos were injected with second non-overlapping MO (MO2) for each gene of interest. The ability of *angptl4* MO2, *cdh13* MO2 and *pdcd2l* MO2 to modify splicing of the targeted transcript was assessed by analysis of RT-PCR products after agarose gel electrophoresis. Splicing modifications were observed as a band shift or as a reduction of the wildtype band compared to the control sample (ctrl MO). RT-PCR analysis of beta-actin (*bact2*) was used as an internal control. The DNA fragment sizes are indicated in base pairs next to the DNA ladder (L). (C) Embryos with residual expression of the wildtype transcript (*angptl4* MO2 in (B)) were additionally tested by qPCR to validate MO efficiency. Mean values are shown with standard error of the mean from three independent experiments. ***$p<0.001$, using an unpaired two-tailed t-test. (D) Embryos injected with either *perp* MO or a non-overlapping MO (MO2) exhibited bursting of yolk cell during dechorionation using forceps, which was not observed in control embryos. Embryos are shown at 30 hpf, lateral view, anterior to the left, dorsal up. Scale bar: 500 μm.

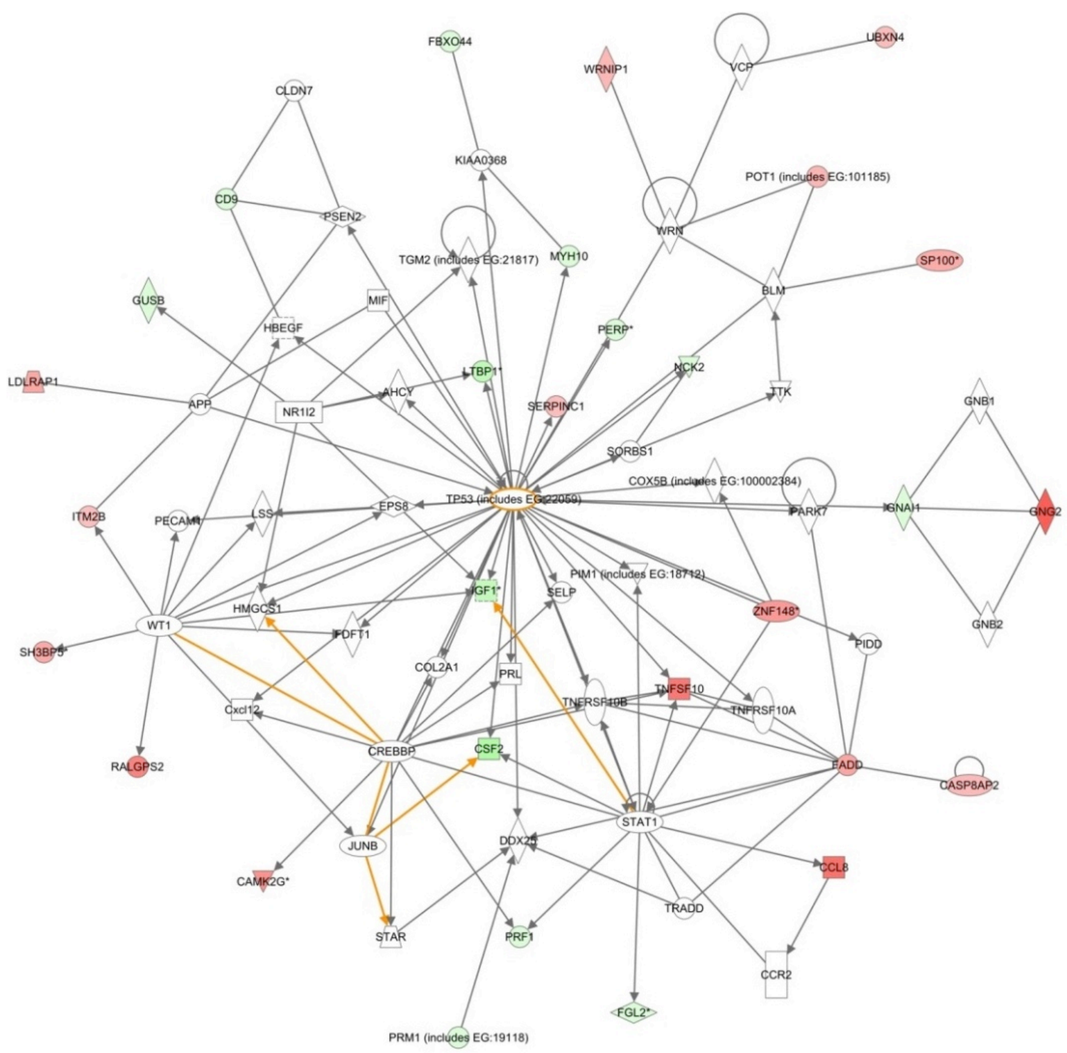

Supplemental Fig XI.

Assessment of differentially expressed genes using Ingenuity Pathway Analysis. The EC transcriptome was studied at high and low WSS regions in 5 pigs using microarrays and differentially expressed genes were annotated using DAVID. Genes with a putative role in apoptosis were assembled into a functional interaction network using Ingenuity Pathway Analysis software. Red represents genes that were enriched at the high WSS site, whereas green marks those enriched at the low WSS region. The shape of the objects indicates their function; inverted triangle, kinase; vertical diamond, enzyme; horizontal oval, transcription factor; circle, other; square, cytokine/growth factor.

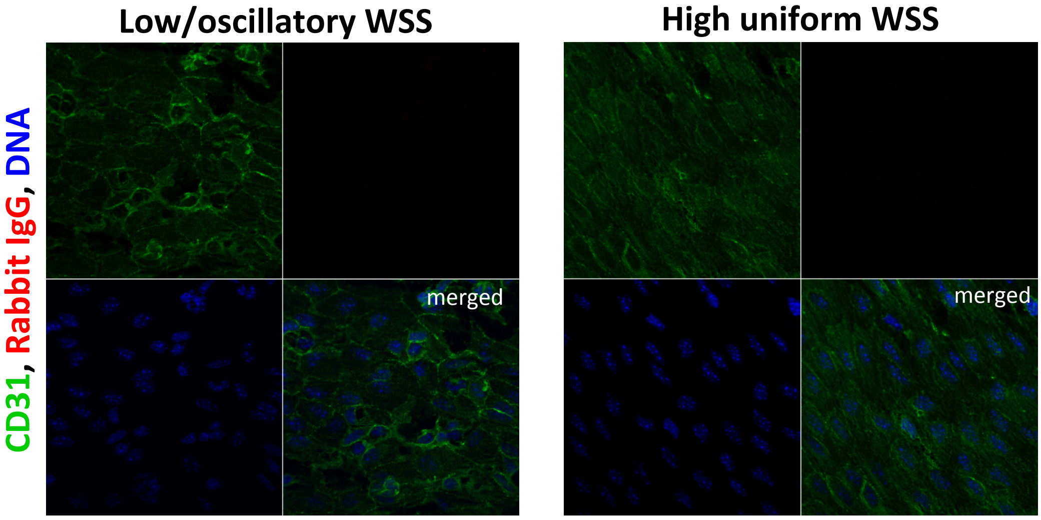

Supplemental Fig XII. Negative control for *en face* staining of murine endothelium. Isotype-matched rabbit IgG control antibody was used to control for specific staining of low/oscillatory WSS (inner curvature) or high WSS (outer curvature) regions of the mouse aortic arch. ECs were identified by co-staining with anti-CD31 antibody conjugated to Alexa Fluor 488 (green). Cell nuclei were identified using To-Pro-3 (blue).

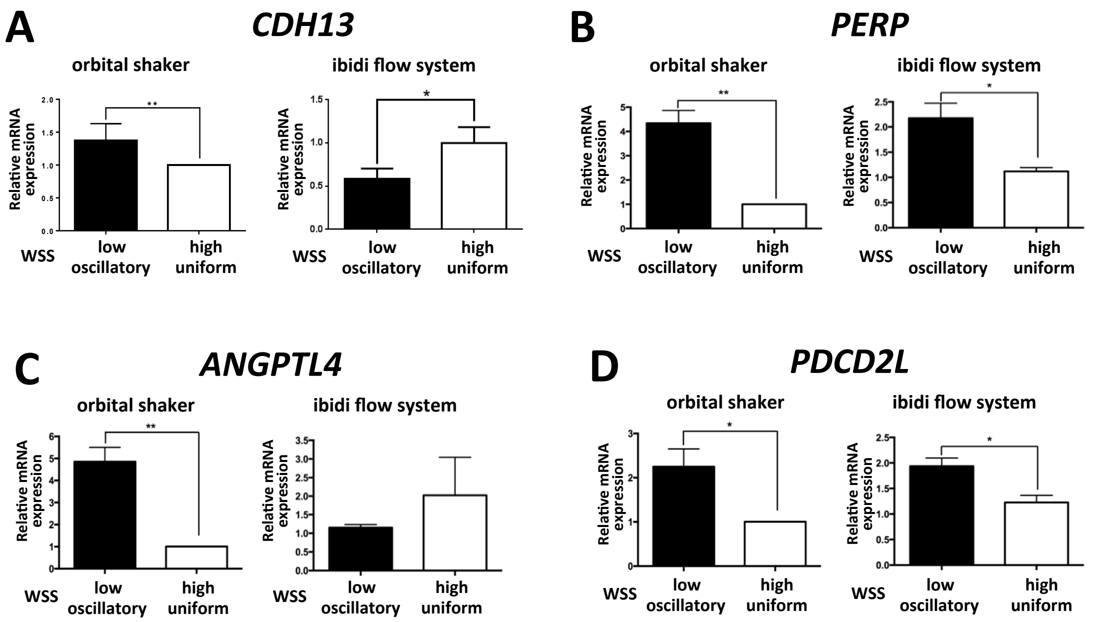

Supplemental Fig XIII. Induction of apoptotic regulators by flow in cultured endothelial cells. PAECs were cultured in 6-well plates and exposed to orbital shaking (left panels) to generate low oscillatory WSS (4.8 dyne/cm^2) at the centre of the well and high uniform WSS (13 dyne/cm^2) at the periphery. Alternatively, they were grown on ibidi microslides (right panels) and exposed to low oscillatory WSS (±4 dyne/cm^2, 1 Hz) or high uniform WSS (13 dyne/cm^2). After 72 h, transcript levels of CDH13 (A), PERP (B), ANGPTL4 (C) and PDCD2L (D) were quantified by qRT-PCR using gene-specific primers. Mean values are shown with standard error of the mean; n=5, *p<0.05, **p<0.01 using an unpaired two-tailed t-test.

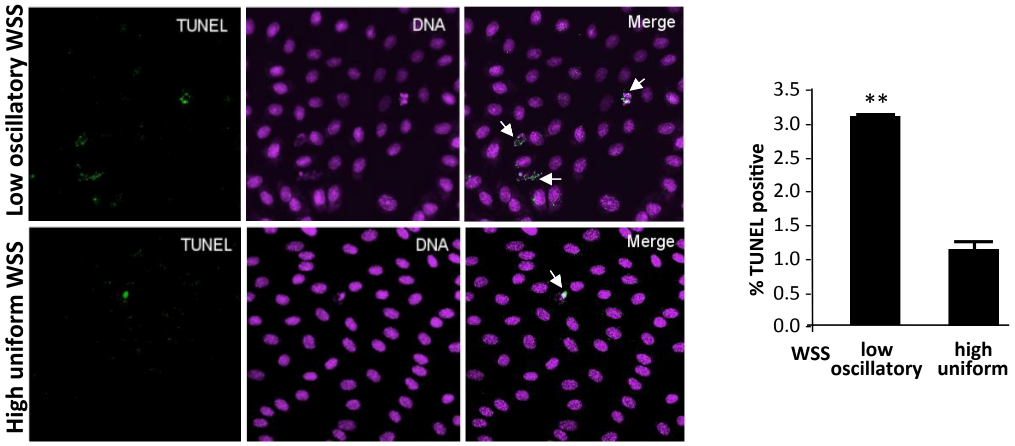

Supplemental Fig XIV. Low oscillatory WSS induced apoptosis in cultured EC. HUVECs cultured in 6-well plates were exposed to orbital shaking (210 rpm) for 72 h. Apoptotic cells were measured by TUNEL (green) with To-Pro-3 counterstaining of nuclei (purple; DNA). Apoptotic ECs are indicated with white arrows. Data from at least three independent experiments were pooled. Mean % TUNEL-positive cells are shown with error of the mean. ** $p<0.01$ using an unpaired two-tailed t-test.

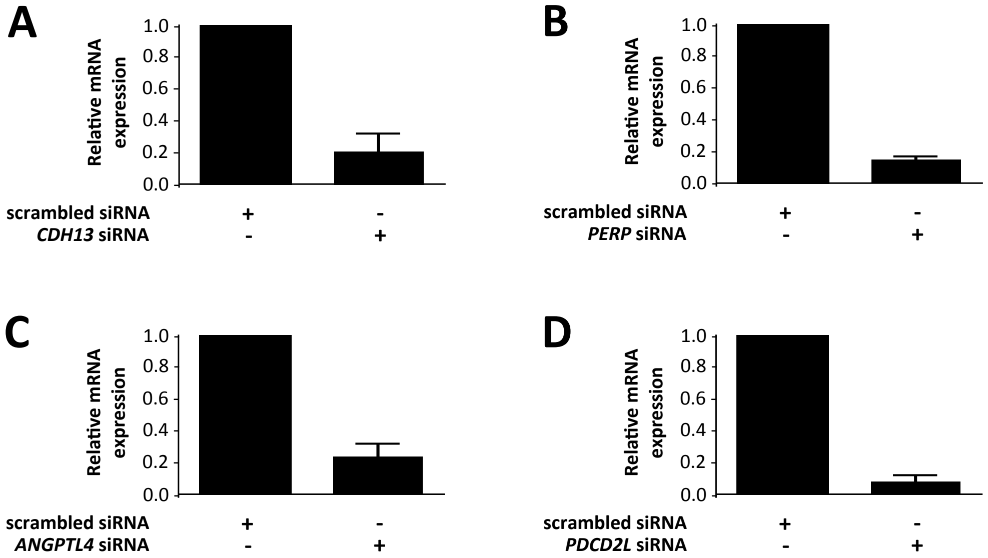

Supplemental Fig XV. Validation of siRNA-mediated gene knockdown using qRT-PCR. HUVECs were transfected with scrambled sequences and *CDH13* (A), *PERP* (B), *ANGPTL4* (C) or *PDCD2L* (D) siRNA and incubated for 24 h. Relative mRNA levels were measured by qRT-PCR and data from three independent experiments are presented as mean with standard error of the mean.

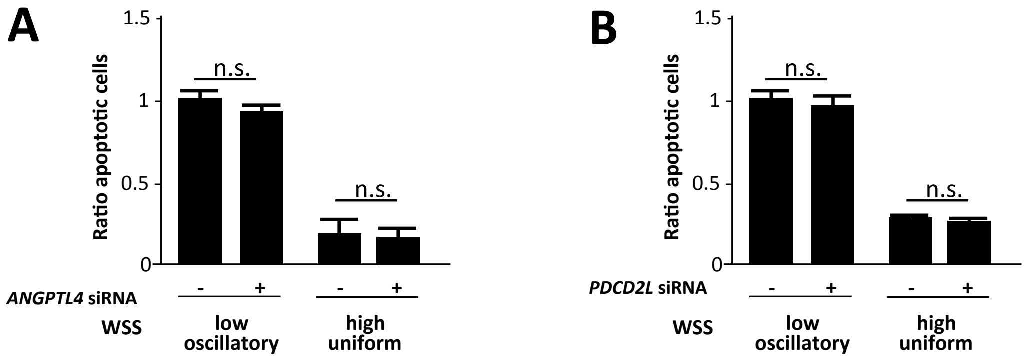

Supplemental Fig XVI. *In vitro* functional study of *ANGPTL4* and *PDCD2L*. HUVECs were transfected with scrambled sequences and ANGPTL4 (A) or *PDCD2L* (B) siRNA and incubated for 24 h. Cells were exposed for 72 h to low oscillatory (centre) or high uniform (periphery) WSS using the orbital system. Apoptotic cells were measured by immunofluorescent staining using antibodies that detect cleaved caspase-3. Data from at least three independent experiments were pooled and the proportion of apoptotic cells are shown with standard error of the mean. ns, no significance by two-way ANOVA.

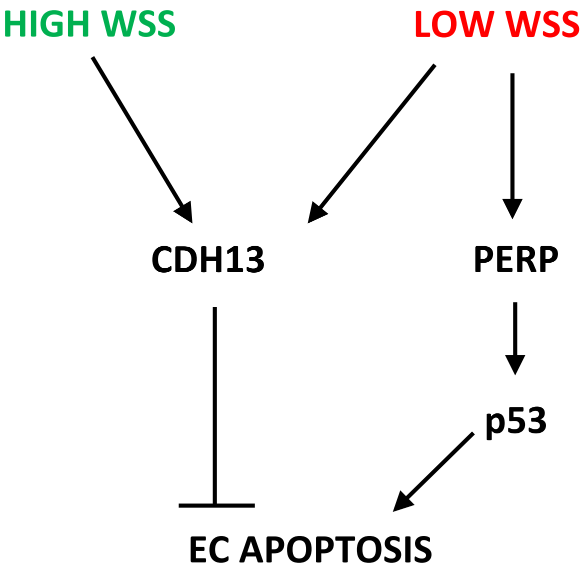

Supplemental Fig XVII. Proposed model of flow-mediated regulation of EC apoptosis by CDH13 and PERP.

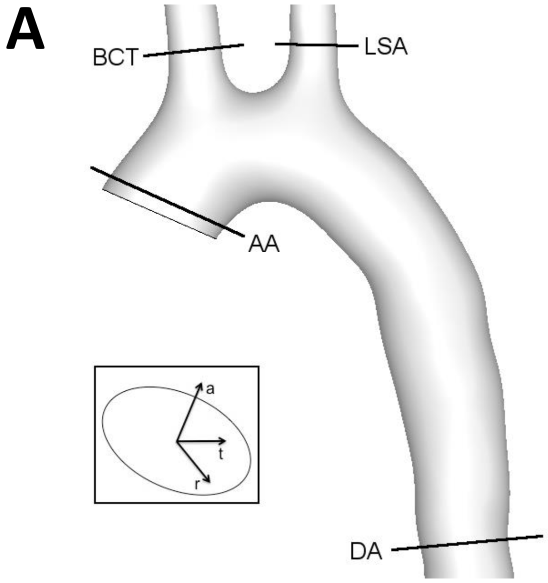

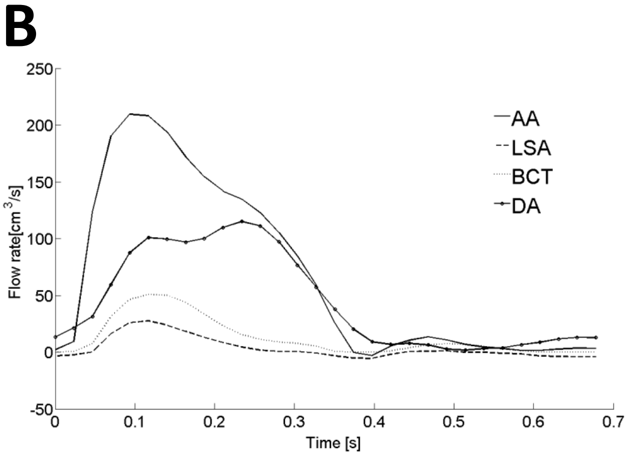

Supplemental Fig. XVIII

Boundary conditions in the porcine aortic arch. (A) Image-based model of the aorta showing the acquisition planes of the phase contrast-MRI scans and velocity components diagram. The flow data were acquired at cross-sections of the ascending aorta (AA), descending aorta (DA), brachiocephalic trunk (BCT) and left subclavian artery (LSA). All three components of the velocity were measured at different time points of the cardiac cycle and used as boundary conditions at the inlet section. The components were expressed in the local cylindrical coordinates system: a- axial, t-tangential, r-radial. (B) Flow rates measured by phase contrast-MRI at different location of the porcine aortic arch. The flow rate was determined by multiplying the through-plane velocity with the area of the cross-section.

Supplemental Table I

	Gene symbol	Regulation in low vs high WSS	Fold change	Probe set ID	Entrez Gene ID
1	HOXA9	↘	19.946184	Ssc.26748.1.A1_at	100519066
2	HOXB7	↘	10.235369	Ssc.22336.1.S1_at	100522513
3	HOXB7	↘	8.086807	Ssc.22336.2.A1_at	100522513
4	HOXB6	↘	5.838337	Ssc.13232.2.A1_at	100627849
5	HOXB6	↘	4.302616	Ssc.13232.2.A1_s_at	100627849
6	CDH13	↘	3.9784467	Ssc.12939.1.S1_at	100126163
7	GNG2	↘	3.936485	Ssc.7256.1.A1_at	100524008
8	FZD8	↘	3.8537142	Ssc.24891.1.S1_at	N/A
9	ARHGAP25	↘	3.5988276	Ssc.19344.1.A1_at	100514401
10	GPR183	↘	3.5231488	Ssc.26709.1.S1_at	100522635
11	C16orf45	↘	3.4913924	Ssc.3796.1.S1_at	100622402
12	HOXD8	↘	3.4763258	Ssc.3450.1.S1_at	100515172
13	PRR15L	↘	3.3300211	Ssc.11487.1.A1_at	100513831
14	CORO2B	↘	3.1937869	Ssc.17073.2.S1_at	100520530
15	GNG2	↘	3.0923002	Ssc.26312.1.S1_at	100524008
16	HOXB4	↘	3.0268426	Ssc.12491.1.A1_at	100522341
17	CD79B	↘	2.9822779	Ssc.12584.1.A1_at	100511898
18	ORC2	↘	2.856432	Ssc.24449.1.S1_at	106510112
19	ARL4A	↘	2.8441467	Ssc.30613.1.A1_at	595121
20	PTPLAD2	↘	2.8129902	Ssc.8808.1.A1_at	100523711
21	ATP10A	↘	2.7874618	Ssc.8206.1.A1_at	100158162
22	EML4	↘	2.7337449	Ssc.926.1.A1_at	100515021
23	EML4	↘	2.7235885	Ssc.3500.1.A1_at	100515021
24	CCL8	↘	2.7186818	Ssc.9957.1.A1_at	100302703
25	EML4	↘	2.7038	Ssc.9509.1.A1_at	100515021
26	STK38L	↘	2.6430771	Ssc.3975.1.A1_at	100516734
27	BCAT1	↘	2.637186	Ssc.12561.1.A1_at	100152656
28	SLA-DOA	↘	2.6332066	Ssc.16038.1.S1_at	100157996
29	HOXB6	↘	2.609709	Ssc.20706.1.S1_at	100627849
30	TNFSF10	↘	2.5715754	Ssc.12829.1.A1_at	406191
31	PUS10	↘	2.491379	Ssc.19632.1.S1_at	100523369
32	THSD7A	↘	2.4507859	Ssc.7865.1.A1_at	100512249
33	PHACTR1	↘	2.4434404	Ssc.9834.1.A1_at	100153737
34	RALGPS2	↘	2.380985	Ssc.21236.1.S1_at	100522752
35	LOC100514323	↘	2.374514	Ssc.29437.1.S1_at	100514323
36	ADORA3	↘	2.3440392	Ssc.4756.1.A1_at	606753
37	FAM126A	↘	2.2778978	Ssc.19532.1.S1_at	100620255
38	TOX	↘	2.2336402	Ssc.2336.1.A1_at	100155888
39	HOXA10	↘	2.2318113	Ssc.12652.1.A1_at	100620451
40	SMARCAD1	↘	2.2300062	Ssc.9911.1.A1_at	100515330
41	RNF144A	↘	2.221699	Ssc.29623.1.A1_at	N/A
42	CHML	↘	2.203723	Ssc.28933.1.S1_at	100521677
43	FGGY	↘	2.1963584	Ssc.5993.1.A1_at	102166471
44	BAR superfamily, new	↘	2.161302	Ssc.14173.1.S1_at	N/A
45	SHPRH	↘	2.0846393	Ssc.22076.1.S1_at	100154314
46	CAMK2G	↘	2.0840695	Ssc.2491.1.S1_at	397394
47	PRR24-like	↘	2.0673795	Ssc.4025.1.S1_at	N/A
48	CYP2U1	↘	2.0584033	Ssc.28083.1.A1_at	100518620
49	GJA5	↘	2.0545404	Ssc.3015.1.S1_at	100157795
50	CDKN2AIP	↘	2.0342124	Ssc.20386.1.S1_at	100513762
51	UGP2	↘	2.0246205	Ssc.30801.1.A1_at	100620996
52	RPS6KA5	↘	2.0002036	Ssc.2132.1.S1_a_at	100152046
53	BAG5	↘	1.9761075	Ssc.4063.1.S1_at	100626167
54	SLA-DRA	↘	1.973723	Ssc.222.1.S1_at	100135040
55	ZNF518A	↘	1.972793	Ssc.7334.1.A1_at	100153530
56	SLA-DMB	↘	1.9677062	Ssc.11025.1.S1_at	100135050
57	CD74	↘	1.9585812	Ssc.6222.1.S1_a_at	396660
58	ZNF148	↘	1.9488881	Ssc.30750.1.A1_at	100515279
59	WDR48	↘	1.9487313	Ssc.3296.1.S1_at	100518629
60	TP53INP1	↘	1.9481462	Ssc.29750.1.A1_at	100157451
61	CALML4	↘	1.9262187	Ssc.4434.1.S1_at	100519528
62	ANKMY2	↘	1.9242777	Ssc.12856.1.A1_at	100514124
63	IKZF5	↘	1.912228	Ssc.24196.2.S1_a_at	100153217
64	JUN	↘	1.9092457	Ssc.22550.1.A1_at	396913
65	TMEM199(C17orf32)	↘	1.9003505	Ssc.4755.1.A1_at	100623454

	Gene symbol	Regulation in low vs high WSS	Fold change	Probe set ID	Entrez Gene ID
66	LRP8	↘	1.8974873	Ssc.31012.1.A1_at	100187577
67	HOXA7	↘	1.8931614	Ssc.13853.1.A1_at	100519456
68	ERCC5	↘	1.8794332	Ssc.6879.1.A1_at	100622935
69	RNF214	↘	1.8783178	Ssc.5610.1.S1_a_at	100510972
70	ANK3	↘	1.8657414	Ssc.13587.1.A1_at	100154687
71	JUN	↘	1.8520523	Ssc.9075.1.A1_at	396913
72	RNF144A	↘	1.8444637	Ssc.18231.1.S1_at	N/A
73	ZNF184	↘	1.8444128	Ssc.30158.1.A1_at	100620153
74	CARD9	↘	1.832908	Ssc.18896.1.A1_at	N/A
75	ARID4A	↘	1.8304753	Ssc.24407.1.S1_at	100153197
76	USP47	↘	1.8230982	Ssc.27154.1.S1_at	100519588
77	Pirh2	↘	1.8075595	Ssc.9304.1.A1_at	100038006
78	TRPM7	↘	1.8037517	Ssc.13606.1.A1_at	100157945
79	NLRC5	↘	1.7982252	Ssc.8942.1.S1_at	100135667
80	SAMD12	↘	1.78391	Ssc.5979.1.A1_at	100738445
81	GGA3	↘	1.7812867	Ssc.6502.1.A1_at	100525777
82	TRAPPC4	↘	1.7777481	Ssc.16983.3.S1_a_at	100516212
83	THSD7A	↘	1.7729049	Ssc.2552.1.A1_at	100512249
84	GPR157	↘	1.7722937	Ssc.2179.1.S1_at	N/A
85	MTAP	↘	1.7709556	Ssc.27049.1.A1_at	100516774
86	FNIP1	↘	1.7639365	Ssc.13512.1.A1_at	100521611
87	IMPA1	↘	1.755362	Ssc.24718.1.S1_at	397425
88	BCAT1	↘	1.7538699	Ssc.18004.1.A1_at	100518784
89	SFXN2	↘	1.7394273	Ssc.18078.1.A1_at	100152984
90	BEND7	↘	1.7391009	Ssc.5800.1.A1_at	N/A
91	CDYL	↘	1.7381872	Ssc.8312.1.A1_at	100155291
92	PDHX	↘	1.7376887	Ssc.7185.1.A1_at	100525559
93	ARID4A	↘	1.737268	Ssc.24155.1.S1_at	100153197
94	FNBP1	↘	1.7287087	Ssc.1801.2.S1_at	100518593
95	ZNF33B	↘	1.720862	Ssc.7510.1.A1_at	100738050
96	TCHP	↘	1.7203513	Ssc.24883.1.A1_at	100518693
97	BCAT1	↘	1.7195811	Ssc.12565.1.A1_at	100152656
98	FADD	↘	1.7169782	Ssc.1947.1.S1_at	595129
99	USP47	↘	1.7168034	Ssc.29734.1.A1_at	100519588
100	FNBP1	↘	1.7161055	Ssc.1801.1.A1_at	100518593
101	MID1IP1	↘	1.7051815	Ssc.25129.1.A1_at	100526197
102	SLA-DQA1	↘	1.7035724	Ssc.11102.1.S1_at	100153387
103	BCAT1	↘	1.702749	Ssc.27431.1.A1_at	100152656
104	C19orf12	↘	1.7026932	Ssc.27761.1.S1_at	100627784
105	SLA-DQB1	↘	1.7002095	Ssc.11063.1.S1_at	100037921
106	XRCC6BP1	↘	1.6985786	Ssc.12964.1.S1_at	100737094
107	TMCC1	↘	1.6962711	Ssc.9518.1.A1_at	100512192
108	P2RX7	↘	1.6942111	Ssc.11608.1.A1_at	497623
109	IKZF5	↘	1.6921072	Ssc.24196.1.S1_at	100153217
110	LPCAT1	↘	1.6901907	Ssc.11242.1.S1_at	100217397
111	EML4	↘	1.6768227	Ssc.23111.1.A1_at	100515021
112	PTGIS	↘	1.6767997	Ssc.2502.1.S1_at	100126284
113	chur	↘	1.6761332	Ssc.14297.1.S1_at	100514910
114	POC5	↘	1.6743059	Ssc.17188.1.A1_at	100519012
115	MUM1	↘	1.6651121	Ssc.3809.1.A1_at	100511862
116	ARID4A	↘	1.6639371	Ssc.8703.1.A1_at	100153197
117	CXorf38	↘	1.6611629	Ssc.12020.1.A1_at	100515115
118	TLK1	↘	1.6509433	Ssc.13037.1.A1_at	100157412
119	CDR2	↘	1.6500056	Ssc.3394.1.A1_at	100626193
120	SYNRG	↘	1.6495525	Ssc.18498.2.A1_at	100624012
121	MKRN2	↘	1.6485447	Ssc.818.2.A1_at	100626891
122	METTL11A	↘	1.6477703	Ssc.19574.1.A1_at	100516660
123	LDLRAP1	↘	1.6468419	Ssc.26245.1.S1_at	100125967
124	CBFA2T2	↘	1.645752	Ssc.11884.1.A1_at	100157067
125	DDI2	↘	1.6439716	Ssc.2004.1.A1_at	100519198
126	LNPEP	↘	1.6434716	Ssc.7777.1.A1_at	100125826
127	ADO	↘	1.6421105	Ssc.11901.1.S1_at	100153304
128	ARID1A	↘	1.641297	Ssc.4617.2.S1_at	100513359
129	SLA-DRB1	↘	1.6394564	Ssc.210.1.S1_a_at	100153386
130	CDR2	↘	1.6380332	Ssc.3394.3.A1_at	100626193

	Gene symbol	Regulation in low vs high WSS	Fold change	Probe set ID	Entrez Gene ID
131	CACUL1	↘	1.6378405	Ssc.16799.1.A1_at	100737227
132	OSGEPL1	↘	1.6310734	Ssc.2595.1.S1_at	100512818
133	FNBP1	↘	1.6246257	Ssc.1801.2.S1_a_at	100518593
134	GOSR1	↘	1.6238722	Ssc.5643.1.A1_at	100517676
135	MAP2K5	↘	1.6234636	Ssc.9944.1.A1_at	N/A
136	CAMK2G	↘	1.6230392	Ssc.5879.4.S1_a_at	397394
137	ZNF148	↘	1.619686	Ssc.21426.1.S1_at	100515279
138	MTG2	↘	1.6172359	Ssc.1334.1.A1_a_at	N/A
139	MAN1A1	↘	1.6165802	Ssc.29024.1.S1_at	396919
140	CUL5(VACM-1)	↘	1.6158081	Ssc.4549.1.A1_at	100525708
141	SH3BP5	↘	1.6143107	Ssc.28336.1.A1_at	100517856
142	ZNF330	↘	1.6103315	Ssc.26825.1.S1_at	100511765
143	BIVM	↘	1.6091915	Ssc.6752.1.S1_at	100512068
144	GPATCH1	↘	1.6069031	Ssc.7644.1.A1_at	100525924
145	SOS2	↘	1.601014	Ssc.18317.1.A1_at	100156602
146	ATAD2B	↘	1.5972787	Ssc.10724.1.A1_at	100518905
147	SH3BP5	↘	1.5942206	Ssc.25282.1.S1_at	100517856
148	SRRM1	↘	1.5931035	Ssc.30202.1.A1_at	100521629
149	SP100	↘	1.5871345	Ssc.7207.3.A1_at	100516940
150	SLA-DRB1	↘	1.5854198	Ssc.210.2.S1_a_at	100153386
151	ELMO2	↘	1.5843375	Ssc.2298.1.S1_at	100153181
152	DNAJC5	↘	1.5836406	Ssc.27168.1.S1_at	100621635
153	C9orf40	↘	1.5814447	Ssc.6388.1.A1_at	100517993
154	SAMD12	↘	1.580022	Ssc.1708.1.S1_at	100738445
155	ATG4B	↘	1.5786996	Ssc.6320.1.A1_at	100462747
156	NKIRAS2	↘	1.5721251	Ssc.23472.1.S1_at	100524706
157	AKAP7	↘	1.5719472	Ssc.29749.1.A1_at	100523651
158	PLA1A	↘	1.5708334	Ssc.15424.1.S1_at	100154368
159	GPATCH2	↘	1.5700215	Ssc.22368.1.A1_at	100513810
160	PRORSD1	↘	1.564619	Ssc.21808.1.S1_at	100514282
161	PHF17	↘	1.5620664	Ssc.13638.2.A1_a_at	100516328
162	HTB2	↘	1.5594175	Ssc.2474.1.S1_at	100523961
163	ANGEL2	↘	1.5574449	Ssc.5494.1.A1_at	100523200
164	TMEM53	↘	1.5562224	Ssc.11335.1.A1_at	100517102
165	RAD17	↘	1.5549328	Ssc.21361.1.A1_at	100515763
166	Luc7-like 1	↘	1.5507433	Ssc.31171.1.S1_at	100737615
167	SUV420H1	↘	1.5492636	Ssc.4394.1.S1_at	100521358
168	MXD1	↘	1.544877	Ssc.10022.1.A1_at	100625349
169	ARV1	↘	1.5389528	Ssc.29739.1.A1_at	100151849
170	APRIL	↘	1.5356606	Ssc.26879.2.S1_at	100127162
171	STX16	↘	1.5334104	Ssc.16389.1.S1_at	100144526
172	APG5	↘	1.5271122	Ssc.2160.1.S1_at	N/A
173	GSKIP	↘	1.5264741	Ssc.1084.1.S1_at	100154584
174	HSDL1	↘	1.5252693	Ssc.21858.1.A1_at	100516372
175	RBM25	↘	1.5239364	Ssc.8506.1.A1_at	100511390
176	NICE-3	↘	1.5239034	Ssc.8693.1.S1_at	100518371
177	CEP57-like	↘	1.5174923	Ssc.8270.1.S1_at	100518322
178	CHD9	↘	1.5151073	Ssc.13364.1.A1_at	N/A
179	ANTXR1	↘	1.5144627	Ssc.20172.1.A1_at	100513853
180	DGUOK	↘	1.5120171	Ssc.10592.1.A1_at	100515889
181	ZNF839	↘	1.5094002	Ssc.26700.1.S1_at	100152925
182	C9orf40	↘	1.5085456	Ssc.22490.1.S1_at	100517993
183	KDM5B	↘	1.5074414	Ssc.17712.2.S1_at	100511772
184	AREL1	↘	1.5067781	Ssc.4181.1.S1_at	100622552
185	PRODH	↘	1.5051893	Ssc.1813.1.S1_at	100524089
186	ANKRD27	↘	1.5026225	Ssc.7196.1.A1_at	100524146
187	GSKIP//C7H14orf129	↘	1.5014261	Ssc.13743.1.S1_at	100154584
188	CCDC56	↘	1.5014015	Ssc.3838.1.S1_at	100518575
189	CMYA5///AP5M1	↘	1.5011969	Ssc.27500.1.A1_at	100513193
190	USP47	↘	1.4958131	Ssc.10129.1.S1_at	100519588
191	C6orf136///DUF2358 superfamily	↘	1.4939694	Ssc.4883.1.A1_at	100152775
192	PIAS3	↘	1.4895221	Ssc.6032.1.S1_at	100511878
193	MAP7D1	↘	1.4885817	Ssc.1951.1.S1_at	100511265
194	RPS19BP1	↘	1.4877795	Ssc.3542.1.S1_at	100518907
195	BIVM	↘	1.4875659	Ssc.19133.1.A1_at	100512068

	Gene symbol	Regulation in low vs high WSS	Fold change	Probe set ID	Entrez Gene ID
196	CALML4	↘	1.4861591	Ssc.6845.1.A1_at	100519528
197	KDM5A	↘	1.4838719	Ssc.19273.3.S1_at	100294704
198	GRB2	↘	1.4834785	Ssc.27313.3.S1_at	100192436
199	CCDC51	↘	1.4813484	Ssc.13063.1.S1_at	100521222
200	ATG10	↘	1.481278	Ssc.11852.1.A1_at	100462744
201	ORC3	↘	1.4805428	Ssc.13441.1.A1_at	100519524
202	PHF17	↘	1.4766477	Ssc.13638.1.A1_a_at	100516328
203	WWP2	↘	1.4722648	Ssc.3721.1.S1_at	100626361
204	SUZ12	↘	1.4693431	Ssc.1286.1.A1_at	100294712
205	MFAP1	↘	1.4691318	Ssc.4348.2.S1_at	100157390
206	HIST1H1D	↘	1.4688598	Ssc.19327.1.S1_at	595122
207	FMR1	↘	1.4685771	Ssc.25167.1.A1_at	100155078
208	LRRK1	↘	1.4668978	Ssc.25233.1.S1_at	100521353
209	SP100	↘	1.4643824	Ssc.7207.2.A1_at	100516940
210	PIN4	↘	1.4642389	Ssc.18633.1.S1_at	100524473
211	RFTN2	↘	1.4624918	Ssc.10247.1.S1_at	100520462
212	ZDHHC7	↘	1.4495702	Ssc.3617.2.S1_at	100514104
213	ZCCHC8	↘	1.4493873	Ssc.4055.1.A1_at	100157314
214	TXLNA	↘	1.4446361	Ssc.1423.1.A1_at	100522017
215	VEZF1	↘	1.444204	Ssc.12255.1.A1_at	100511406
216	RCAN3	↘	1.4438319	Ssc.28328.2.A1_at	100522144
217	ATP8A1	↘	1.4417562	Ssc.19482.1.A1_at	100515393
218	STAT5B	↘	1.4411074	Ssc.16047.2.S1_at	397340
219	DNAJB2	↘	1.4407461	Ssc.1180.1.S1_at	100522168
220	FRA10AC1	↘	1.4406893	Ssc.832.1.S1_at	100157308
221	SMAP2	↘	1.4406629	Ssc.3072.1.S1_at	100145888
222	LRP6	↘	1.4392573	Ssc.4693.3.S1_a_at	100515777
223	UBP1	↘	1.4332042	Ssc.10876.1.S1_at	100512254
224	NSMAF	↘	1.426117	Ssc.22275.1.S1_at	100153039
225	CCDC117	↘	1.424431	Ssc.7745.1.A1_at	100737460
226	PRPF18	↘	1.4238194	Ssc.13735.1.A1_at	100511773
227	ZNF238	↘	1.4221326	Ssc.21986.1.S1_at	100623289
228	HMGCL	↘	1.4191952	Ssc.27317.1.S1_at	100621218
229	TP53INP1	↘	1.4178016	Ssc.30871.1.A1_at	100157451
230	PLXDC2	↘	1.4153379	Ssc.12603.1.A1_at	100522568
231	TOR1AIP2	↘	1.4144878	Ssc.1620.1.A1_at	100627970
232	KDM5A	↘	1.4138042	Ssc.19273.1.A1_at	100294704
233	SLA-DMB	↘	1.4122739	Ssc.12882.1.A1_at	100135050
234	TPR	↘	1.411703	Ssc.22099.2.A1_at	100520507
235	LXN	↘	1.4093919	Ssc.2983.1.A1_at	100620551
236	TMEM87A	↘	1.4078664	Ssc.30974.1.S1_at	100154984
237	ZNF605	↘	1.4067224	Ssc.31180.1.A1_at	100155634
238	SLA-3	↘	1.405378	Ssc.13780.9.S1_a_at	100037288
239	LIMD1	↘	1.404453	Ssc.30639.1.S1_at	100738579
240	NAGA	↘	1.4030142	Ssc.8873.1.A1_at	100142667
241	ZFHX3	↘	1.3994361	Ssc.8422.1.A1_at	100519194
242	BPTF	↘	1.3985949	Ssc.1385.1.S1_at	100525424
243	GPATCH2L	↘	1.3966646	Ssc.11508.1.A1_at	100153141
244	AUH	↘	1.3953178	Ssc.12622.1.A1_a_at	100155020
245	UPF0561///C2orf68	↘	1.3938141	Ssc.21620.1.S1_at	100620143
246	FLAG1	↘	1.3910651	Ssc.24281.1.A1_at	N/A
247	ZNF652	↘	1.3900046	Ssc.30952.1.A1_at	100524338
248	POT1	↘	1.3834908	Ssc.21430.1.S1_at	100192443
249	NOXP20	↘	1.38339	Ssc.3021.1.A1_at	100525173
250	LRIG2	↘	1.3807962	Ssc.3003.1.S1_at	100154795
251	KDM2A	↘	1.3797257	Ssc.24207.1.A1_at	100294703
252	MAP4K3	↘	1.3770274	Ssc.16445.1.A1_at	100519831
253	IDE	↘	1.376735	Ssc.9109.1.A1_at	100155309
254	ZNF292	↘	1.3766184	Ssc.19121.1.A1_at	100517252
255	TOMM5	↘	1.3762743	Ssc.872.1.S1_a_at	100520533
256	GCP60	↘	1.3742001	Ssc.26214.1.A1_at	100621148
257	DDX6	↘	1.3736061	Ssc.7089.1.A1_at	100515499
258	SUGP2	↘	1.3712014	Ssc.1300.1.A1_at	100511863
259	TTC17	↘	1.3693042	Ssc.18899.1.A1_at	100521838
260	BRD8	↘	1.3691893	Ssc.7279.1.A1_at	100518182

	Gene symbol	Regulation in low vs high WSS	Fold change	Probe set ID	Entrez Gene ID
261	MAX	↘	1.367563	Ssc.19313.1.A1_at	100517170
262	CHEK1	↘	1.3660244	Ssc.29650.1.A1_at	100511709
263	RCBTB1	↘	1.364094	Ssc.28150.1.S1_at	100628042
264	FMR1	↘	1.3638347	Ssc.27393.1.S1_at	100155078
265	UPF3B	↘	1.3625139	Ssc.22179.1.A1_at	414433
266	ACTR6	↘	1.3621515	Ssc.24251.1.S1_a_at	100620672
267	RAB11FIP2	↘	1.3594759	Ssc.13291.1.S1_at	100153733
268	LOC100525937	↘	1.3582821	Ssc.3494.1.A1_at	100525937
269	ISCU	↘	1.3582519	Ssc.7879.1.S1_at	100621691
270	MITD1	↘	1.357193	Ssc.10408.1.S1_at	100523366
271	TTC33	↘	1.3568549	Ssc.18637.1.A1_at	100625161
272	ZEB1	↘	1.3561156	Ssc.9299.1.S1_at	100520325
273	FAM104A	↘	1.3535918	Ssc.4995.3.A1_at	100518034
274	GBA2	↘	1.3533192	Ssc.15216.1.S1_at	100155655
275	HDAC5	↘	1.3532587	Ssc.4125.1.A1_at	100515165
276	ANKS6	↘	1.3529372	Ssc.21901.1.S1_at	100152629
277	FAM134A/C2orf17	↘	1.3463604	Ssc.1366.1.S1_at	100156393
278	WRNIP1	↘	1.3448923	Ssc.2136.1.S1_at	100155921
279	SUN1	↘	1.3429621	Ssc.10938.1.S1_at	100522311
280	MGP	↘	1.3408589	Ssc.373.1.S1_at	397206
281	TNPO3	↘	1.3394185	Ssc.4478.1.A1_at	100515455
282	LIMCH1	↘	1.3393929	Ssc.10466.1.A1_at	100515040
283	SORT1	↘	1.3387518	Ssc.12492.2.S1_at	100157862
284	NTAN1	↘	1.3386791	Ssc.149.1.S1_at	397107
285	ANKS1A	↘	1.3371844	Ssc.29819.1.A1_at	100625697
286	GSK3B	↘	1.3361373	Ssc.22547.1.S1_at	100126852
287	ZKSCAN5	↘	1.3333199	Ssc.2755.1.S1_at	100522312
288	FUBP3	↘	1.3288034	Ssc.23802.1.S1_at	100524188
289	ANKRD54	↘	1.3260444	Ssc.24098.2.S1_at	100525518
290	VPS35	↘	1.325336	Ssc.26019.1.S1_at	100623578
291	RAB2B	↘	1.3239303	Ssc.8370.1.A1_at	100513669
292	RORA	↘	1.3221625	Ssc.26039.1.S1_at	100156637
293	DCTD	↘	1.3218298	Ssc.2007.1.S1_at	100154836
294	UPF3B	↘	1.3214906	Ssc.2192.1.A1_at	414433
295	TRUB2	↘	1.3195932	Ssc.7494.1.A1_at	100157964
296	CHMP4A	↘	1.3166322	Ssc.1636.1.S1_at	100520257
297	TSPAN14	↘	1.3161929	Ssc.2569.1.S1_at	100154486
298	BAZ1B	↘	1.3067816	Ssc.20056.1.S1_at	100625872
299	ZFAND2B	↘	1.3051437	Ssc.21640.2.S1_a_at	100157982
300	PAP7	↘	1.3037138	Ssc.19058.1.S1_at	494461
301	PARP12	↘	1.3035105	Ssc.13068.1.A1_at	100515805
302	ERCC8	↘	1.3022738	Ssc.25036.1.S1_at	100524405
303	PIAS1	↘	1.2991236	Ssc.5358.1.S1_at	100519005
304	CASP8AP2	↘	1.2983356	Ssc.24845.1.S1_at	100523759
305	MSTN	↘	1.297279	Ssc.24083.1.A1_at	399534
306	UBE2R2	↘	1.2966303	Ssc.4948.1.S1_at	100512675
307	LZTS2	↘	1.2957842	Ssc.2100.1.S1_at	100625806
308	BBS4	↘	1.291044	Ssc.15249.1.S1_at	100513212
309	DYNC1LI2	↘	1.2897233	Ssc.7287.1.A1_at	100626901
310	FAM207A	↘	1.2878661	Ssc.17751.1.S1_at	100623984
311	DYNC1I2	↘	1.2864742	Ssc.1201.1.S1_at	100153352
312	ZDHHC17	↘	1.2854146	Ssc.24119.1.A1_at	100514712
313	TAX1BP1	↘	1.2815381	Ssc.6722.1.A1_at	100516165
314	KDM5A	↘	1.2805303	Ssc.13085.1.S1_at	100294704
315	CNTF	↘	1.2793785	Ssc.3599.1.S1_at	100518821
316	TPCN2	↘	1.2793741	Ssc.16652.1.S1_at	100523421
317	TPP2	↘	1.2760087	Ssc.6979.1.A1_at	100511152
318	TJP2	↘	1.2743537	Ssc.16907.1.A1_at	100513065
319	TAB2	↘	1.2734888	Ssc.4161.1.A1_at	100156182
320	MYH9	↘	1.2697904	Ssc.17295.1.S1_at	396904
321	PLXDC2	↘	1.2587172	Ssc.25615.1.A1_at	100522568
322	FBXO38	↘	1.2569319	Ssc.9650.1.S1_a_at	100513273
323	TBL1X	↘	1.2552079	Ssc.19636.1.S1_at	100624648
324	ZNF131	↘	1.2532858	Ssc.6837.1.A1_at	100525619
325	TPP2	↘	1.2508465	Ssc.14205.1.S1_at	100511152

	Gene symbol	Regulation in low vs high WSS	Fold change	Probe set ID	Entrez Gene ID
326	SEC31A	↘	1.2483761	Ssc.11165.1.S1_at	100511210
327	RtcB	↘	1.2483679	Ssc.5319.1.S1_at	733658
328	OTUD3	↘	1.2482762	Ssc.2328.1.S1_at	N/A
329	UPF3B	↘	1.2478641	Ssc.26341.1.S1_at	414433
330	CLCN3	↘	1.2473717	Ssc.29052.1.S1_at	100156049
331	FAM188A	↘	1.2466104	Ssc.15034.1.S1_at	100525772
332	TBC1D23	↘	1.2441875	Ssc.1010.1.A1_at	100524904
333	C22orf25	↘	1.2415887	Ssc.2850.3.S1_at	100154308
334	COMMD9	↘	1.2364765	Ssc.2256.1.S1_at	100524189
335	FAM21A	↘	1.2362039	Ssc.27237.2.S1_a_at	100522401
336	UBXN4	↘	1.2351348	Ssc.28413.1.A1_at	100525432
337	SERPINC1	↘	1.2318238	Ssc.27560.1.S1_at	100125972
338	FAM188A	↘	1.2272383	Ssc.19336.1.A1_s_at	100525772
339	ARIH1	↘	1.2271239	Ssc.19028.1.A1_at	100511268
340	B2M	↘	1.2261658	Ssc.12348.2.S1_at	397033
341	HBP1	↘	1.2246569	Ssc.6645.1.S1_at	100519099
342	RAB35	↘	1.2177399	Ssc.17391.1.S1_at	100151805
343	CHAF1A	↘	1.2136545	Ssc.7857.1.A1_at	100524302
344	SPTBN1	↘	1.2130053	Ssc.16046.1.S1_s_at	100516258
345	C19orf42	↘	1.2107177	Ssc.3048.1.S1_at	100512765
346	VPS25	↘	1.2105951	Ssc.3188.1.S1_at	100622124
347	ZNF652	↘	1.2101111	Ssc.5954.3.S1_at	100524338
348	CSNK2A1	↘	1.208754	Ssc.28057.1.A1_at	100517435
349	ZNF10	↘	1.2079518	Ssc.10898.1.A1_at	100627773
350	AIFM2	↘	1.2025254	Ssc.2487.1.S1_at	100153541
351	URM1	↘	1.2002077	Ssc.2196.1.S1_at	100153888
352	ARGRS(RARS)	↘	1.1973017	Ssc.24387.1.S1_at	414410
353	MORC2	↘	1.1969198	Ssc.1645.1.S1_at	100153895
354	CCDC30	↘	1.1952308	Ssc.15180.1.A1_a_at	100522685
355	PPT1	↘	1.194955	Ssc.11457.1.A1_at	100517533
356	SERINC3	↘	1.1948549	Ssc.887.1.A1_at	100152356
357	TGFBRAP1	↘	1.1939412	Ssc.1387.2.S1_at	100625522
358	NAA40	↘	1.1928564	Ssc.3711.1.S1_at	100523044
359	ARPC1B	↘	1.1889032	Ssc.15456.1.S1_at	100174964
360	ITM2B	↘	1.1888913	Ssc.1037.1.S1_at	595120
361	NEU1	↘	1.1865914	Ssc.23310.1.S1_at	100124381
362	TMSB4X	↘	1.180933	Ssc.4122.1.A1_at	100216317
363	NEK9	↘	1.1795983	Ssc.13939.1.A1_at	100152772
364	ZNF84	↘	1.1780202	Ssc.28314.1.S1_at	100620909
365	PSMF1	↘	1.1777529	Ssc.6353.1.S1_at	100152620
366	GTF3C5	↘	1.1738005	Ssc.17447.1.A1_at	100514889
367	PSIP1	↘	1.1710576	Ssc.1539.1.S1_at	100157597
368	AMBRA1	↘	1.1700011	Ssc.6243.2.S1_at	100519700
369	VPS28	↘	1.164719	Ssc.10875.1.A1_at	100154859
370	RCOR3	↘	1.1619703	Ssc.24585.1.S1_at	100510922
371	POC1A	↘	1.1592215	Ssc.26425.1.S1_at	100521113
372	TDP54/TPD52L2	↘	1.157296	Ssc.2916.1.A1_at	N/A
373	NR2C1	↘	1.1504481	Ssc.10207.1.A1_at	100516014
374	NCOR1	↘	1.1484798	Ssc.26588.1.S1_at	100037950
375	CCDC72	↘	1.1472111	Ssc.5928.1.A1_at	100521518
376	SEC16A	↘	1.1368035	Ssc.1322.1.S1_at	102163628
377	VRK1	↘	1.134457	Ssc.24353.2.S1_at	100157930
378	TGFBRAP1	↘	1.1259986	Ssc.1387.1.S1_at	100625522
379	TMSB4X	↘	1.1208929	Ssc.4122.1.A1_s_at	100216317
380	GBP2	↘	1.1172758	Ssc.17889.1.A1_at	100523668
381	GUCY1A2	↘	1.1166422	Ssc.17485.1.S1_at	100522091
382	ND3	↘	1.1161274	SscAffx.29.1.S1_at	808508
383	NPEPL1	↘	1.1109015	Ssc.11230.1.A1_at	100144530
384	TNRC6B	↘	1.0964177	Ssc.4265.1.A1_a_at	100625754
385	LAPTM4A	↘	1.0928482	Ssc.5153.1.S1_at	100516188
386	ND2	↘	1.0871962	SscAffx.33.1.S1_at	100515155
387	VPS39	↘	1.0653918	Ssc.17266.1.S1_at	100513254
388	SLA-2	↘	1.0586841	Ssc.13780.12.S1_a_at	100135031
389	SLC7A7	↗	1.0645334	Ssc.3715.1.S1_at	100037972
390	TYRP1	↗	1.0833911	Ssc.13311.1.A1_at	100516717

	Gene symbol	Regulation in low vs high WSS	Fold change	Probe set ID	Entrez Gene ID
391	PAK7	↗	1.0846866	Ssc.26858.1.A1_at	100152316
392	PNPLA6	↗	1.091802	Ssc.11227.1.S1_at	100524423
393	POLDIP3	↗	1.0919214	Ssc.11339.2.A1_at	100525164
394	HAUS7	↗	1.0920017	Ssc.26999.1.S1_at	100518818
395	ELF5	↗	1.09305	Ssc.9472.1.A1_at	100510890
396	USP16	↗	1.0939736	Ssc.11900.1.A1_at	100623735
397	PTGR1	↗	1.0973716	Ssc.26521.1.A1_at	397678
398	CDC42EP5	↗	1.0975119	Ssc.17268.1.A1_at	100525753
399	PTC7	↗	1.0980562	Ssc.24027.2.A1_at	100513685
400	ACADL	↗	1.1014236	Ssc.12672.1.A1_at	396931
401	LRRC37A2	↗	1.1025498	Ssc.1406.1.S1_at	100514982
402	CC2D1B	↗	1.1046464	Ssc.9403.1.A1_at	100622838
403	Psmc3ip	↗	1.1049509	Ssc.12881.1.A1_at	100521397
404	Kazal-type 14	↗	1.1053799	Ssc.26171.1.S1_at	100739287
405	PERP	↗	1.1080321	Ssc.11670.1.A1_at	100513507
406	TCEB1	↗	1.1082404	Ssc.3883.2.S1_a_at	100153381
407	ID4	↗	1.1102786	Ssc.9645.1.S1_at	100511514
408	MCRS1	↗	1.1110886	Ssc.5149.1.S1_at	100516550
409	SUMO1	↗	1.112161	Ssc.8741.1.A1_at	100127139
410	ASIC2	↗	1.1129644	Ssc.21266.2.A1_at	100518193
411	BDH1 /// LOC100737308	↗	1.1139408	Ssc.18245.1.A1_at	100155725 /// 100737308
412	ERI3	↗	1.1149908	Ssc.27329.2.S1_at	100521097
413	DEFB108B	↗	1.1189868	Ssc.25487.1.S1_at	692190
414	ACTN2	↗	1.1194898	Ssc.763.1.S1_at	100157406
415	RBMS1	↗	1.1196808	Ssc.6805.2.A1_at	100525434
416	ITFG3	↗	1.1199899	Ssc.2475.1.S1_at	100523363
417	TMEM255A	↗	1.1225525	Ssc.27980.1.A1_at	100152537
418	C12orf23	↗	1.1228079	Ssc.9952.1.S1_at	100620181
419	KANSL3	↗	1.1254379	Ssc.30201.1.A1_at	N/A
420	PKHD1L1	↗	1.1264888	Ssc.12455.1.S1_at	100525460
421	DMRT-1	↗	1.126523	Ssc.321.1.S1_at	397199
422	FGA-like	↗	1.1296525	Ssc.4824.1.A1_at	100626178
423	RNF180	↗	1.1313637	Ssc.26611.1.A1_at	100526254
424	DNAL1	↗	1.1328927	Ssc.29457.1.A1_at	100156521
425	EIF5A2	↗	1.133744	Ssc.21681.1.A1_at	N/A
426	GUSB	↗	1.1339793	Ssc.18585.2.S1_at	100144519
427	KIAA1549	↗	1.1356556	Ssc.26991.1.A1_at	100519232
428	PUS10	↗	1.1361166	Ssc.31105.1.A1_at	100523369
429	TCRB	↗	1.1372119	Ssc.11075.11.A1_at	100621664
430	PQLC2	↗	1.1373869	Ssc.16528.2.S1_at	100511513
431	WBP2	↗	1.1380539	Ssc.17837.1.A1_at	100521149
432	NDOR1	↗	1.139997	Ssc.15685.1.A1_at	100515645
433	DYRK2	↗	1.1403315	Ssc.9629.2.S1_at	100516081
434	CASP14	↗	1.1405201	Ssc.22118.1.A1_at	100518472
435	FGFR1IIIC	↗	1.1412214	Ssc.17773.1.A1_at	396569
436	MLYCD	↗	1.1412755	Ssc.18522.2.S1_at	497060
437	PMEL	↗	1.1417292	Ssc.29472.1.S1_at	100739533 /// 594851
438	PUSL1	↗	1.1437527	Ssc.2881.1.S1_at	100524091
439	SLC30A9	↗	1.1442661	Ssc.18392.1.A1_at	100192440
440	Igh	↗	1.1454268	Ssc.16359.8.S1_at	406179
441	AKR1CL1	↗	1.14671	Ssc.11273.1.A1_at	654406
442	HTR1D	↗	1.1467996	Ssc.15993.1.S1_at	397309
443	BRCA1	↗	1.1468185	Ssc.29471.1.A1_at	100049662
444	ARSE	↗	1.1468554	Ssc.11950.1.A1_at	100627778
445	BPI (by EST blast and blastp)	↗	1.1496148	Ssc.8960.1.A1_at	100286800
446	PTPN13	↗	1.1512711	Ssc.26643.1.A1_at	100521910
447	APLN	↗	1.1515021	Ssc.3436.1.A1_at	100625006
448	BD123	↗	1.1515585	Ssc.26164.1.S1_at	100188899
449	MUC5AC	↗	1.1522598	Ssc.16035.1.S1_at	100170143
450	TRAP1	↗	1.152323	Ssc.23776.2.A1_at	100515536
451	SPINT2	↗	1.1524436	Ssc.9221.1.A1_at	100515207
452	LENG8	↗	1.1531029	Ssc.10310.1.A1_at	100524682
453	NR2F1-AS1	↗	1.1533303	Ssc.29939.1.S1_at	100739512
454	ZNF268	↗	1.1536694	Ssc.24014.1.S1_at	100154415
455	SNX30	↗	1.1537154	Ssc.26027.1.A1_at	100516535

	Gene symbol	Regulation in low vs high WSS	Fold change	Probe set ID	Entrez Gene ID
456	GIMAP4	↗	1.154642	Ssc.24714.1.A1_at	100518347
457	PARP10	↗	1.1549338	Ssc.29337.1.S1_at	100516730
458	FAM113C	↗	1.1560291	Ssc.7159.1.A1_at	100514992
459	ATP6AP1	↗	1.1574922	Ssc.24844.1.A1_at	100514338
460	ERGIC1	↗	1.1580459	Ssc.23508.2.S1_at	100523575
461	TCRB	↗	1.1585102	Ssc.11075.15.A1_a_at	100621664
462	FGFR2IIIC	↗	1.1587648	Ssc.15839.1.A1_at	396695
463	PACRG	↗	1.1589947	Ssc.8104.2.A1_at	100623618
464	BRI3BP	↗	1.159981	Ssc.27497.1.A1_at	100155348
465	HARBI1	↗	1.1601418	Ssc.5597.2.A1_at	100516314
466	ANO5	↗	1.1612085	Ssc.19189.1.S1_at	100520542
467	KATNA1	↗	1.1621672	Ssc.29141.2.A1_at	100518101
468	ACTR3	↗	1.1631625	Ssc.8934.1.A1_at	396738
469	PRM1	↗	1.1648579	Ssc.16223.1.S1_at	397487
470	NPY2R	↗	1.1649932	Ssc.15963.1.S1_at	397291
471	ACOX3	↗	1.1655188	Ssc.5024.1.A1_at	100523005
472	SH3GL2	↗	1.1655504	Ssc.21026.1.S1_at	100155580
473	TCRA	↗	1.1670271	Ssc.17808.1.A1_at	497055
474	TEDDM1	↗	1.1692576	Ssc.4748.2.S1_at	100512916
475	TFF1	↗	1.1693053	Ssc.22946.1.S1_at	780401
476	ZNF786	↗	1.1699095	Ssc.11355.1.A1_at	100522564
477	TBP10	↗	1.1714108	Ssc.7397.1.A1_at	397043
478	c1orf21	↗	1.1717743	Ssc.12188.1.A1_at	100516567
479	TMEM243	↗	1.1721556	Ssc.24660.1.A1_at	100521508
480	AGO2	↗	1.1723416	Ssc.18916.1.A1_at	100155245
481	EPAS1	↗	1.1724627	Ssc.3921.1.S1_at	100037272
482	ADAP1	↗	1.1727148	Ssc.16178.1.S1_at	397453
483	UPK3BL	↗	1.1727328	Ssc.6024.2.S1_at	100511499
484	COL6A1	↗	1.1729673	Ssc.5895.1.A1_at	100101553
485	MFGE8	↗	1.1750984	Ssc.16345.1.S1_at	397545
486	SLC2A6	↗	1.1752713	Ssc.11583.1.A1_at	100627201
487	HS3ST3B1	↗	1.1753402	Ssc.28368.1.A1_at	100522213 /// 100737777
488	C9orf50	↗	1.1756134	Ssc.21887.1.S1_at	100520534
489	TMX1	↗	1.175931	Ssc.6827.1.A1_at	100627887
490	GNAO1	↗	1.1760039	Ssc.21259.1.S1_at	100516976
491	NCK2	↗	1.1761718	Ssc.11693.1.A1_at	100192439
492	EHMT2	↗	1.1762805	Ssc.12499.1.A1_at	100124382
493	MAPK11	↗	1.1765591	Ssc.29722.1.S1_at	N/A
494	CYP2C42	↗	1.1776668	Ssc.16124.1.S1_a_at	403111
495	HNF1B	↗	1.1780761	Ssc.16447.1.S1_at	397002
496	CAR	↗	1.1823049	Ssc.13115.1.A1_at	397333
497	LY6G5C	↗	1.1826866	Ssc.21798.2.S1_a_at	100521803
498	LOC100152387	↗	1.18276	Ssc.18776.1.A1_at	100152387
499	PPFIBP1	↗	1.1837773	Ssc.11536.1.S1_at	100516194
500	ZFP36L2//BRF2	↗	1.183882	Ssc.2423.1.A1_at	100524530
501	KRTAP26-1	↗	1.184228	Ssc.20452.1.S1_at	100624997
502	KNG1	↗	1.1862148	Ssc.14075.1.A1_at	396568
503	GNL3L	↗	1.186483	Ssc.3822.2.S1_at	100521120
504	LOC100627367	↗	1.1866461	Ssc.19833.1.S1_at	100627367
505	COX8C	↗	1.1871048	Ssc.25517.1.S1_at	100157935
506	SYNDIG1L	↗	1.1874386	Ssc.19269.1.S1_at	100737341
507	NUP50	↗	1.1887549	Ssc.1790.3.A1_at	100519837
508	SPEG	↗	1.1910249	Ssc.27402.1.A1_at	100737722
509	HOXD9	↗	1.1912377	Ssc.23551.1.S1_at	100517431
510	HYDIN	↗	1.1913779	Ssc.29504.1.A1_at	100513208
511	LTN1	↗	1.1930491	Ssc.13630.3.A1_at	106505847
512	TCRA	↗	1.1946489	Ssc.15850.1.S1_a_at	497055
513	RBM47	↗	1.1949266	Ssc.9438.1.A1_at	100513606
514	EFEMP2	↗	1.1963735	Ssc.4267.3.S1_at	100526031
515	NHE5///SLC9A5	↗	1.1965882	Ssc.15982.1.A1_at	396753
516	NETO1//BCTL1//BTCL1	↗	1.1967146	Ssc.19095.1.A1_at	100626670
517	DR-1 beta chain	↗	1.1967152	Ssc.16212.2.A1_x_at	100153139
518	CEP170	↗	1.1967354	Ssc.14149.1.A1_at	100623007
519	ADAT3	↗	1.1980525	Ssc.24941.1.S1_at	100525631
520	P2RX2	↗	1.1986394	Ssc.19283.1.A1_at	100736581

	Gene symbol	Regulation in low vs high WSS	Fold change	Probe set ID	Entrez Gene ID
521	SIGLEC-1	↗	1.199109	Ssc.17338.1.S1_at	397623
522	CEBPD	↗	1.1997335	Ssc.10025.3.S1_at	100153946
523	PRF1	↗	1.2001395	Ssc.19693.1.A1_at	396595
524	KRTAP11-1	↗	1.2011951	Ssc.25697.1.S1_at	100626583
525	C12orf52	↗	1.2014875	Ssc.18225.3.S1_at	100523867
526	UBXN10	↗	1.201903	Ssc.12920.1.A1_at	100514294
527	EPT1	↗	1.2024547	Ssc.27288.1.S1_at	100512162
528	LOC100622406	↗	1.2036941	Ssc.30191.1.A1_at	N/A
529	WIPF2	↗	1.203806	Ssc.24454.1.A1_at	100525252
530	GIF	↗	1.2045074	Ssc.15504.1.S1_at	100514273
531	FLYWCH1	↗	1.205208	Ssc.6143.2.S1_at	100736802
532	TCRA	↗	1.2057694	Ssc.17793.1.A1_at	497055
533	G6PD	↗	1.2058585	Ssc.23537.1.S1_at	407058
534	TBX3	↗	1.2061691	Ssc.28678.1.S1_at	100152741
535	HNRPDL	↗	1.2063463	Ssc.7731.1.A1_at	100511704
536	IGSF9B	↗	1.206591	Ssc.27100.1.A1_at	100626845
537	B4GALT3	↗	1.2074867	Ssc.16825.1.S1_at	100154856
538	CD9	↗	1.2078965	Ssc.54.1.A1_at	397067
539	PER1	↗	1.2085786	Ssc.16979.2.A1_at	100620648
540	ELSPBP1	↗	1.2088342	Ssc.17775.1.S1_a_at	399529
541	WDR91	↗	1.2101955	Ssc.25330.2.S1_at	100524054
542	WDR27	↗	1.211905	Ssc.19372.2.A1_at	100511666
543	PPAP2C	↗	1.2128613	Ssc.4862.1.A1_at	100233200
544	WDTC1	↗	1.2138182	Ssc.11356.1.A1_at	100190994
545	CRYBB1	↗	1.2143898	Ssc.22340.1.S1_at	780429
546	OATV1	↗	1.2151083	Ssc.17335.1.S1_at	397621
547	FXYD3	↗	1.2165403	Ssc.569.1.S1_at	397413
548	PTOV1	↗	1.216926	Ssc.21379.1.S1_at	100522621
549	CXXC5	↗	1.2170271	Ssc.9048.2.A1_at	100524854
550	PDZK1IP1	↗	1.2173697	Ssc.19735.1.S1_at	414756
551	FGF7	↗	1.2184622	Ssc.15923.1.S1_at	397281
552	ROGDI	↗	1.2184838	Ssc.2167.3.A1_at	100525282
553	AMIGO3	↗	1.2199185	Ssc.20781.2.S1_at	100625501
554	PRDX5	↗	1.220114	Ssc.460.1.S1_at	397273
555	RRP4	↗	1.2211516	Ssc.31177.1.S1_at	100523112
556	EPB41L1	↗	1.2226721	Ssc.2070.1.S1_at	100156952
557	SERPINB1	↗	1.2228085	Ssc.26522.1.A1_at	100155145
558	EMSP1	↗	1.2231773	Ssc.11.1.S1_at	396720
559	BRI3BP	↗	1.2248338	Ssc.26416.1.A1_at	100155348
560	DEDD	↗	1.2253324	Ssc.1294.2.S1_at	100156474
561	FBXO44	↗	1.2254227	Ssc.5427.1.A1_at	100524444
562	GMEB2	↗	1.2257701	Ssc.16417.1.A1_at	N/A
563	OXT	↗	1.2259587	Ssc.15668.1.A1_at	100152272
564	CDX2	↗	1.2272046	Ssc.24504.1.S1_at	100127132
565	GPC4	↗	1.2272341	Ssc.29487.1.A1_at	100156029
566	OIP5//protein Mis18-beta	↗	1.2276815	Ssc.24992.1.S1_at	100516409 /// 100621171
567	Rab-3D	↗	1.2292842	Ssc.11705.2.A1_at	100516180
568	NFAT	↗	1.2292883	Ssc.16122.1.S1_at	100134969 /// 396824
569	GARNL3	↗	1.2294141	Ssc.19598.2.A1_at	100156351
570	ADCY1	↗	1.2295872	Ssc.26388.1.S1_at	100624934
571	EDNRA	↗	1.2300621	Ssc.16189.1.S1_at	397457
572	PHYHD1	↗	1.2306986	Ssc.22015.2.S1_at	100157556
573	C19orf54	↗	1.2318456	Ssc.21879.1.S1_at	100524024
574	FHOD3	↗	1.2323215	Ssc.17896.2.S1_at	100622006
575	CRYGF	↗	1.2325692	Ssc.6779.1.S1_at	100522220
576	COUP-TF1///NR2F1	↗	1.2343899	Ssc.3282.2.A1_at	100621562
577	GNAI1	↗	1.2352043	Ssc.16259.1.A1_at	397505
578	PANK3	↗	1.2365003	Ssc.24636.1.A1_at	100512746
579	TMEM82	↗	1.2371538	Ssc.26115.1.S1_at	100522322
580	BLG	↗	1.2388949	Ssc.11136.1.S1_at	396596
581	GAD2	↗	1.2393881	Ssc.5021.1.S1_at	396929
582	RBP1	↗	1.2396388	Ssc.5563.2.S1_a_at	100156666
583	CYP2C42	↗	1.2400304	Ssc.16124.1.A1_a_at	403111
584	ZNF22	↗	1.2415313	Ssc.7980.1.A1_at	100739086
585	FAM20C	↗	1.2439616	Ssc.4698.1.A1_at	100628219

	Gene symbol	Regulation in low vs high WSS	Fold change	Probe set ID	Entrez Gene ID
586	ARSJ	↗	1.2458606	Ssc.3579.1.A1_at	100512548
587	GEMIN5	↗	1.2461165	Ssc.27501.1.S1_at	100524914
588	BPGM	↗	1.2473524	Ssc.22500.1.S1_at	100525495
589	LHCGR	↗	1.2473683	Ssc.16311.1.A1_a_at	407247
590	CCDC51	↗	1.2479284	Ssc.18683.1.S1_at	100521222
591	SNAP91/ or AP180	↗	1.2484759	Ssc.10571.1.S1_at	100521763
592	PLB1	↗	1.2498362	Ssc.30851.1.S1_at	100519306
593	RHOF	↗	1.2503896	Ssc.2019.3.S1_at	100144500
594	CHN1	↗	1.2514935	Ssc.23258.1.S1_at	100627143
595	CELF4	↗	1.2517583	Ssc.22048.3.A1_at	100521138
596	RCD1	↗	1.2522762	Ssc.21425.1.S1_at	100153939
597	LCLAT1	↗	1.2532785	Ssc.24706.1.A1_at	100217396
598	Obsl1	↗	1.2548671	Ssc.27615.2.S1_a_at	100624019
599	RBM12B	↗	1.2553035	Ssc.13106.1.S1_at	100514101
600	SLAMF9///CD84-H1///CD2F-10	↗	1.2559308	Ssc.11060.2.S1_at	100525572
601	SLC24A4	↗	1.2565199	Ssc.12727.1.A1_at	100156914
602	PLOD1	↗	1.2576838	Ssc.23519.1.S1_at	100525583
603	FGFBP3	↗	1.258201	Ssc.23917.1.A1_at	100153311
604	VCP	↗	1.2586975	Ssc.27050.2.A1_at	100624357
605	SMG6	↗	1.2589691	Ssc.28383.1.S1_at	100511407
606	TEF1	↗	1.2592355	Ssc.15152.2.S1_at	100216478
607	OCA2	↗	1.2593017	Ssc.15775.1.S1_at	397171
608	PNLIPRP2	↗	1.2596288	Ssc.18827.1.A1_at	100462755
609	ELK3	↗	1.2597055	Ssc.15671.1.S1_at	100622445
610	CXCL2	↗	1.2624699	Ssc.24282.2.S1_at	396594
611	FBLN7	↗	1.2629337	Ssc.6510.1.A1_at	100517582
612	KCNH2	↗	1.263125	Ssc.30718.1.A1_at	100523293
613	UPK2	↗	1.2638063	Ssc.70.1.S1_at	397075
614	CITED4	↗	1.2643626	Ssc.9605.1.A1_at	100525468
615	OPRL1	↗	1.2649717	Ssc.16086.1.S1_at	397364
616	PROM2	↗	1.2656176	Ssc.27157.2.S1_at	100521959
617	UTS2	↗	1.2664226	Ssc.437.1.S1_a_at	397268
618	CACNB2	↗	1.2673753	Ssc.10614.1.A1_at	100524101
619	POLM	↗	1.2677318	Ssc.19244.2.A1_at	100515113
620	PVRL1	↗	1.2685801	Ssc.15891.1.S1_at	397247
621	KIAA1737	↗	1.2690537	Ssc.12687.1.A1_at	100157174
622	UBQLN1	↗	1.2702641	Ssc.23541.1.S1_at	100622592
623	B4GALNT1	↗	1.2702726	Ssc.14898.3.S1_at	100516440
624	CDC42EP2	↗	1.2709657	Ssc.5357.2.S1_at	100513778
625	PVRL1	↗	1.2735168	Ssc.6982.2.S1_at	397247
626	RANBP2	↗	1.2738378	Ssc.21677.1.S1_at	100511376
627	ELK3	↗	1.2739894	Ssc.4212.2.S1_at	100622445
628	OX-2///MRC///CD200	↗	1.2767805	Ssc.13223.1.A1_at	100517427///100626182
629	PUS1	↗	1.2792598	Ssc.22201.2.S1_a_at	100157224
630	LRCH2	↗	1.2798992	Ssc.13457.1.A1_at	100155794
631	DHX36	↗	1.2807215	Ssc.22088.2.A1_at	100626674
632	FOXA1	↗	1.2819997	Ssc.26021.1.S1_at	100156502
633	SLC22A17	↗	1.2849393	Ssc.2181.2.A1_a_at	100152841
634	SLC38A1	↗	1.2854768	Ssc.28490.2.A1_at	100154364
635	ELK3	↗	1.2874928	Ssc.4212.1.A1_at	100622445
636	KIFC1	↗	1.2880796	Ssc.25117.1.A1_at	100154627 /// 100737015
637	POLRMT	↗	1.2889692	Ssc.4451.2.S1_at	N/A
638	MUC16	↗	1.2907802	Ssc.30342.1.A1_at	100624460
639	DCLK2	↗	1.292085	Ssc.12389.1.A1_s_at	100520394
640	ATP10B	↗	1.2939444	Ssc.5545.1.S1_at	100626997
641	URM1	↗	1.2939484	Ssc.8888.1.A1_at	100153888
642	PAQR3	↗	1.294048	Ssc.24474.1.A1_at	100514591
643	MYO5B	↗	1.2966125	Ssc.10382.1.A1_at	100521829
644	HOX7/MSX1	↗	1.3002042	Ssc.9355.2.A1_at	100302364
645	KIAA0146/SPIDR	↗	1.3021438	Ssc.27496.2.S1_a_at	100153946
646	CSRNP2	↗	1.3033892	Ssc.8048.1.S1_at	N/A
647	SSU72	↗	1.3046336	Ssc.27067.1.A1_at	100525522
648	ZNF697	↗	1.3056176	Ssc.9659.1.A1_at	100154558
649	RCD1	↗	1.3061107	Ssc.21425.2.S1_at	100153939
650	LOC100518881	↗	1.3071729	Ssc.25612.1.S1_at	100518881

	Gene symbol	Regulation in low vs high WSS	Fold change	Probe set ID	Entrez Gene ID
651	FAM183A	↗	1.3072073	Ssc.30511.1.A1_at	100520438
652	FAM129C	↗	1.3077534	Ssc.6341.3.A1_at	100517808
653	GLYR1	↗	1.3082824	Ssc.18242.2.A1_at	100525456
654	Obsl1	↗	1.3089406	Ssc.27615.1.S1_at	100624019
655	MYLPF	↗	1.3096435	Ssc.20141.1.S1_at	474162
656	TMEM9	↗	1.3104694	Ssc.9010.1.A1_at	100621878
657	TMSB4X	↗	1.3108249	Ssc.27304.3.S1_s_at	733606///100216317
658	HYAL3	↗	1.3109261	Ssc.26118.1.S1_at	404696
659	TCEAL3	↗	1.3131984	Ssc.21736.1.S1_at	100524295
660	PDE4A	↗	1.3147727	Ssc.16123.2.S1_at	396825
661	NPPC	↗	1.3153703	Ssc.23867.1.A1_at	493772
662	CACNA1C	↗	1.3199488	Ssc.4599.1.A1_at	100518733
663	TMEM9	↗	1.3199672	Ssc.19052.1.S1_s_at	100621878
664	LARP1B	↗	1.3212991	Ssc.7941.1.A1_at	100621145///100516502
665	PGM3	↗	1.3231328	Ssc.4307.1.A1_at	100156015
666	HIF1A	↗	1.3235685	Ssc.5542.1.A1_at	396696
667	RCE1	↗	1.3236566	Ssc.5482.1.S1_at	100514389
668	GCLC	↗	1.3260359	Ssc.29035.1.S1_at	100522018
669	MLPH	↗	1.3264482	Ssc.13047.1.A1_at	100049680
670	GCM1	↗	1.328211	Ssc.27605.1.A1_at	414910
671	GRAMD1B	↗	1.330685	Ssc.30948.1.S1_at	100512979
672	WDR26	↗	1.3406295	Ssc.25783.1.S1_at	100523327
673	EDNRB	↗	1.3435119	Ssc.4436.1.S1_at	414911
674	SLC2A3	↗	1.3472246	Ssc.1674.1.A1_at	100626468
675	SYT2	↗	1.3483729	Ssc.12033.1.A1_at	100511582
676	CPXM1	↗	1.348984	Ssc.30175.1.A1_at	100512144
677	MAP4K4	↗	1.3508885	Ssc.3574.1.A1_at	100516547
678	FBLN7	↗	1.3561664	Ssc.13129.1.S1_at	102162371///100517582
679	TRABD	↗	1.3605528	Ssc.2473.1.S1_at	100519151
680	APLNR	↗	1.363571	Ssc.28818.2.S1_at	N/A
681	HOOK1	↗	1.3717499	Ssc.27144.1.A1_at	100525467
682	LOC100515911	↗	1.3733765	Ssc.489.1.A1_at	100515911
683	SPRYD4	↗	1.3752017	Ssc.26129.2.S1_at	100522549
684	EGFL8	↗	1.3766663	Ssc.3386.1.A1_at	100144521
685	SL44-1///TP23	↗	1.3772082	SscAffx.2.1.S1_a_at	100049648///100113399
686	GPC4	↗	1.3776945	Ssc.29046.1.S1_at	100156029
687	SYP	↗	1.378416	Ssc.2798.1.S1_at	100512029
688	DUSP14	↗	1.3805935	Ssc.4140.1.A1_at	100623524
689	POC1B	↗	1.391997	Ssc.7790.1.S1_at	102161501
690	SLC6A8	↗	1.4038124	Ssc.2555.1.S1_at	100302506
691	PPAG3	↗	1.4113176	Ssc.15906.1.S1_at	396731
692	HSPA8	↗	1.4163022	Ssc.19203.3.S1_at	100511890
693	ISYNA1	↗	1.4261355	Ssc.15723.1.S1_at	102164826
694	BTBD9	↗	1.4316114	Ssc.18243.2.S1_at	100156900
695	SOCS1	↗	1.4342244	Ssc.26216.1.S1_at	100307052
696	THRB	↗	1.4385114	Ssc.18017.1.A1_at	396776
697	CEACAM16	↗	1.4467767	Ssc.6653.1.S1_at	100524994
698	APOA1	↗	1.4504682	Ssc.807.1.S1_at	397691
699	GNL2	↗	1.4668972	Ssc.25247.1.S1_at	100623150
700	UGCG	↗	1.4696635	Ssc.17674.1.A1_at	100152737
701	DDX56	↗	1.4734495	Ssc.1418.2.S1_at	100513388
702	LMNA	↗	1.477065	Ssc.944.1.A1_at	100126859
703	CHST3	↗	1.4915618	Ssc.19021.1.A1_at	100151968
704	QSOX1	↗	1.4988298	Ssc.22049.2.S1_at	100620285///100524948
705	ATP6V1C2	↗	1.5039257	Ssc.14999.1.A1_at	100512277
706	MSN	↗	1.5053484	Ssc.14470.1.S1_at	494458
707	MYH10	↗	1.5057305	Ssc.16320.1.S1_at	396903
708	LPPR3	↗	1.5059197	Ssc.30674.1.S1_at	100511492
709	PEG10	↗	1.5084172	Ssc.13476.1.A1_at	654416
710	F11R	↗	1.5191276	Ssc.29060.1.S1_at	100127138
711	SPCS3	↗	1.5228368	Ssc.25099.2.A1_at	100526140
712	HOXA1	↗	1.5344396	Ssc.24582.1.S1_at	100520162
713	WNK1	↗	1.5369571	Ssc.21658.1.S1_at	406235
714	GPR4	↗	1.5376071	Ssc.5191.1.S1_at	100144489
715	CACHD1	↗	1.5380137	Ssc.16511.1.A1_at	100522413

	Gene symbol	Regulation in low vs high WSS	Fold change	Probe set ID	Entrez Gene ID
716	MPP6	↗	1.5437312	Ssc.25376.1.S1_at	100523707
717	DOCK7	↗	1.5455594	Ssc.12264.1.A1_at	100524027
718	FGL2	↗	1.5498251	SscAffx.9.1.S1_at	448808
719	PLEK2	↗	1.5514617	Ssc.5696.1.S1_at	100154251
720	PLSCR4	↗	1.5515579	Ssc.8585.1.A1_at	100518400
721	COL5A1-like	↗	1.5694759	Ssc.4993.1.A1_at	100517021
722	MED24	↗	1.572418	Ssc.2777.1.S1_at	100511100
723	MPP6	↗	1.572611	Ssc.25098.1.S1_at	100523707
724	C14orf37	↗	1.5760218	Ssc.17533.1.A1_at	100514619
725	APLNR	↗	1.5916635	Ssc.28818.2.S1_a_at	N/A
726	RANBP6	↗	1.5917401	Ssc.12070.1.A1_at	100518819
727	SLIT2	↗	1.5942512	Ssc.18000.1.A1_at	100515328
728	PDXK	↗	1.5953624	Ssc.11170.1.S1_at	396983
729	VEGFA	↗	1.6028883	Ssc.15740.1.S2_at	100737169
730	QPRT	↗	1.6068634	Ssc.21948.1.S1_at	100623339
731	TGFBR3	↗	1.6085724	Ssc.1176.1.A1_at	397512
732	CKAP4	↗	1.6154914	Ssc.2147.1.A1_at	100523493
733	TFF2	↗	1.6292584	Ssc.650.1.S1_at	397420
734	VEGFA	↗	1.629887	Ssc.15740.2.S1_a_at	397157
735	PIM3	↗	1.6491135	Ssc.26274.1.S1_at	100521016
736	GBI1/GNAI1	↗	1.6505636	Ssc.4159.1.A1_at	100144419
737	SOCS1	↗	1.6602714	Ssc.9016.1.A1_at	100307052
738	TCTN3	↗	1.6613333	Ssc.5488.1.S1_at	100157208
739	HIC2	↗	1.6657511	Ssc.2117.1.S1_at	100151900
740	LMNA	↗	1.6698565	Ssc.5112.1.S1_at	100126859
741	YRDC	↗	1.6826388	Ssc.18351.1.A1_at	N/A
742	ACOT4	↗	1.6855203	Ssc.18475.3.A1_at	100158115
743	CYP7B1	↗	1.702667	Ssc.24330.1.S1_at	100286867
744	TAF9	↗	1.704668	Ssc.10395.1.A1_at	100516765
745	GPX3	↗	1.7121379	Ssc.19694.1.S1_at	396598
746	MTHFD1L	↗	1.7407426	Ssc.9594.1.A1_at	100154722
747	SH3GL2	↗	1.742462	Ssc.11014.1.S1_at	100523947
748	ATP6V1C2	↗	1.7488481	Ssc.31160.1.A1_s_at	100512277
749	HIF1A	↗	1.7536794	Ssc.390.1.A1_at	396696
750	B4GALNT1	↗	1.7606215	Ssc.14898.2.S1_at	100516440
751	C1QTNF1///ASGR2	↗	1.7689973	Ssc.16889.1.S1_at	100217377///100739379
752	AUTS2	↗	1.7699014	Ssc.4018.1.S1_at	100515476///100515305
753	COL5A1	↗	1.7749091	Ssc.9002.1.A1_at	100517021
754	HIF1A	↗	1.7991	Ssc.390.2.S1_at	396696
755	HEXIM1	↗	1.8042284	Ssc.5567.1.S1_at	100520763
756	FAM176A	↗	1.818726	Ssc.4914.1.A1_at	100510899
757	MMP11	↗	1.823085	Ssc.12514.1.A1_at	100153503
758	SULT1A1	↗	1.8380601	Ssc.20006.1.S1_at	396640
759	MFF	↗	1.8573767	Ssc.13356.1.A1_at	100513442
760	ENO2	↗	1.8645983	Ssc.3743.1.S1_at	100157750
761	FGL2	↗	1.8646586	Ssc.22050.1.S1_s_at	448808
762	SPRED3	↗	1.8792586	Ssc.11998.1.A1_at	100515836
763	NR2F2	↗	1.8818611	Ssc.1205.1.S1_at	100153983
764	CCT2	↗	1.897664	Ssc.27956.1.S1_at	100157776
765	SBNO2	↗	1.916344	Ssc.15443.1.S1_at	100623993
766	SERPINI1	↗	1.9305116	Ssc.6948.1.A1_at	100154352
767	LOC100519434	↗	1.9326838	Ssc.23518.1.S1_at	100519434
768	FAM162B	↗	1.9403174	Ssc.6162.1.A1_at	100152527
769	SDC4	↗	1.9517932	Ssc.17671.1.S1_at	100513446
770	BEX4	↗	1.9586672	Ssc.15648.1.S1_at	100627578
771	GLRB	↗	1.9608474	Ssc.6613.2.S1_at	414284
772	PLSCR4	↗	1.9621781	Ssc.23099.1.S1_at	100518400
773	PTGES	↗	1.9849402	Ssc.1310.1.S1_at	654407
774	DAAM1	↗	1.9917033	Ssc.6363.1.A1_at	100517445
775	CNKSR3	↗	2.0014892	Ssc.19060.1.A1_at	100513248
776	FBXL21	↗	2.0188441	Ssc.28616.1.S1_at	100517085
777	C14orf37	↗	2.0270092	Ssc.6265.1.S1_at	100514619
778	SEMA6A	↗	2.080907	Ssc.8359.2.A1_at	100522008
779	SDC4	↗	2.1011586	Ssc.16324.1.S1_at	397528
780	FBXL21	↗	2.1066568	Ssc.22965.1.S1_at	100738540

	Gene symbol	Regulation in low vs high WSS	Fold change	Probe set ID	Entrez Gene ID
781	FZD4	↗	2.1126251	Ssc.1093.1.A1_at	100520319
782	RGMB	↗	2.1185	Ssc.24542.1.S1_at	100525212
783	TNIP1	↗	2.1350734	Ssc.1758.2.S1_at	100271903
784	CRISPLD1	↗	2.1435142	Ssc.5995.1.A1_at	100152961
785	CCDC146	↗	2.1511903	Ssc.30397.1.A1_at	100515400
786	KIF1A	↗	2.154493	Ssc.21055.1.S1_at	100517246
787	ZDHHC9//CREB1	↗	2.1780782	Ssc.5382.1.A1_at	397449
788	CFD	↗	2.2085614	Ssc.11074.1.S1_at	396877
789	CCDC3	↗	2.2272286	Ssc.6478.2.S1_at	100510975
790	LGALS1	↗	2.2496119	Ssc.1320.1.A1_at	414915
791	NR2F2	↗	2.2973657	Ssc.8676.1.S1_at	100153983
792	MT1A	↗	2.308658	Ssc.16125.1.S1_x_at	397417
793	COL3A1	↗	2.3442357	Ssc.11302.1.S2_at	100152001
794	ODZ1	↗	2.3673534	Ssc.27124.1.A1_at	100512817
795	C13orf33	↗	2.3995767	Ssc.29441.1.A1_at	100153765
796	EBI3	↗	2.406718	Ssc.22203.1.A1_at	100522599
797	ZDHHC9//CREB1	↗	2.429038	Ssc.5382.1.A1_a_at	397449
798	MT2B	↗	2.4343574	Ssc.15640.1.S1_at	396827
799	C5orf49	↗	2.437258	Ssc.949.1.S1_at	100516406
800	DCBLD2///ST3GAL6	↗	2.4527943	Ssc.974.1.S1_at	106504106///100739492
801	KIRREL3	↗	2.4736516	Ssc.8469.1.A1_at	100515566
802	OAF	↗	2.4834223	Ssc.3626.1.S1_at	100522940
803	PLXNB2	↗	2.5031343	Ssc.4084.1.S1_at	N/A
804	C11ORF96	↗	2.5595481	Ssc.17312.1.A1_at	N/A
805	LNX1	↗	2.566624	Ssc.6738.1.S1_at	100523319
806	PERP	↗	2.578564	Ssc.11670.2.S1_at	100737354
807	MASP1	↗	2.5849395	Ssc.18836.1.A1_at	100152125
808	FAM162B	↗	2.5934143	Ssc.6162.2.S1_at	100152527
809	INHBA	↗	2.5974898	Ssc.8555.1.A1_at	100517068
810	PDCD2L	↗	2.606395	Ssc.3994.2.S1_at	100512169
811	INHBA	↗	2.642614	Ssc.17133.1.A1_at	100517068
812	HEBP2	↗	2.7178597	Ssc.12634.1.S1_at	100155860
813	PDGFRA	↗	2.7331629	Ssc.13288.1.A1_at	100524633
814	COL3A1	↗	2.7345514	Ssc.11302.1.S1_at	100152001
815	GYG1	↗	2.7369404	Ssc.4204.1.S1_at	100520641
816	TMEM30B	↗	2.771129	Ssc.26634.1.A1_at	100738730
817	IGF1	↗	2.787531	Ssc.16231.3.S1_a_at	397491
818	SLIT2	↗	2.7902956	Ssc.24342.2.A1_at	100516326
819	PFN2	↗	2.83466	Ssc.8274.1.A1_at	100621523
820	FZD5	↗	2.9598398	Ssc.29483.1.A1_at	100519058
821	PSAT1	↗	2.9640298	Ssc.19839.1.S1_at	100154160
822	CAPNS1 /// GPX3	↗	2.9742532	Ssc.7158.1.A1_a_at	396598
823	GLRB	↗	2.9794476	Ssc.6613.1.A1_at	414284
824	SLIT2	↗	3.0029802	Ssc.7743.1.A1_at	100620577
825	IL4R	↗	3.0874224	Ssc.6256.1.A1_at	397614
826	FZD5	↗	3.1512866	Ssc.13439.1.A1_at	100519058
827	MT1A	↗	3.236157	Ssc.600.1.S1_s_at	397417
828	GATA4	↗	3.4314933	Ssc.3566.2.A1_at	397555
829	IGF1	↗	3.4831822	Ssc.16231.1.S1_a_at	397491
830	LOC100738697	↗	3.5855603	Ssc.26466.1.A1_at	100738697
831	PTPN13	↗	3.6742935	Ssc.9101.1.A1_at	100521910
832	vof16	↗	3.692876	Ssc.23827.1.A1_at	N/A
833	BMP2	↗	3.7096426	Ssc.29004.1.S1_at	100152318 /// 100157103
834	SIX1	↗	3.7122993	Ssc.15233.1.S1_at	100156847
835	CCL2	↗	3.7170718	Ssc.657.1.A1_at	397422
836	DSP	↗	3.7226565	Ssc.1623.1.S1_at	100156744
837	IGF1	↗	3.8359864	Ssc.12578.1.A1_at	397491
838	SCG5	↗	3.8957596	Ssc.155.1.S1_at	397110
839	CCDC3	↗	3.9688344	Ssc.6478.1.A1_at	100510975
840	IGF1	↗	3.9869041	Ssc.31130.1.A1_at	397491
841	LRFN5	↗	4.080709	Ssc.26533.1.A1_at	100156782
842	HAND2	↗	4.092096	Ssc.31068.1.A1_at	100153751
843	FBN2	↗	4.125346	Ssc.27703.1.S1_at	100048956
844	ANGPTL4	↗	4.150375	Ssc.17345.1.S1_at	397628
845	IGF1	↗	4.3058763	Ssc.25004.1.S1_at	397491

	Gene symbol	Regulation in low vs high WSS	Fold change	Probe set ID	Entrez Gene ID
846	LTBP1	↗	4.3194466	Ssc.8072.1.A1_at	100512100
847	CHRDL1	↗	4.463381	Ssc.11310.1.A1_at	100521058
848	TACSTD2	↗	4.6750283	Ssc.2627.2.S1_at	100510966
849	LTBP1	↗	4.693407	Ssc.8072.2.A1_at	100512100
850	THBS4	↗	4.9116945	Ssc.1411.1.S1_at	100513005
851	ANGPTL4	↗	5.260369	Ssc.8980.1.A1_at	397628
852	MGST2	↗	5.350628	Ssc.21635.1.A1_at	100513365
853	MKX	↗	5.7160025	Ssc.23899.1.A1_at	100620286
854	CSF2	↗	5.7550044	Ssc.382.1.S1_at	397208
855	CSF3	↗	5.769807	Ssc.16151.1.S1_at	100737548 /// 396839
856	AUTS2	↗	7.6464424	Ssc.31175.1.S1_at	100515476///100515305
857	GPM6A	↗	8.029271	Ssc.14007.1.A1_at	100511106
858	TWIST1	↗	8.346752	Ssc.17121.1.S1_at	100516456
859	BCHE	↗	8.569331	Ssc.18164.1.A1_at	100624138
860	CHRDL1	↗	10.030324	Ssc.13276.1.A1_at	100521058
861	PCOLCE	↗	10.041028	Ssc.1122.1.S1_at	100627524
862	TMEFF2	↗	10.662961	Ssc.19235.1.A1_at	654831
863	NRXN3	↗	11.1694355	Ssc.24855.1.S1_at	100620189
864	BCHE	↗	11.50771	Ssc.31095.1.A1_at	100624138
865	TMEM158	↗	16.815687	Ssc.17364.1.S1_at	100620777
866	GABRA4	↗	17.019104	Ssc.8348.1.A1_at	100622712
867	CHRDL1	↗	46.29664	Ssc.11310.2.A1_at	100521058

Supplemental Table I

List of genes that were differentially expressed at high and low WSS regions of the porcine aorta. The EC transcriptome was studied at low and high WSS regions of the porcine aorta in 5 pigs using GeneChip® Porcine Genome Arrays (Affymetrix). Data were analysed using GeneSpring software GX 11 (Agilent). Genes that displayed significant fold enrichment between low and high WSS regions are listed. Green indicates decreased, whereas red indicates increased expression in low WSS compared to high WSS site.

Gene Ontology term	Number of Genes	Enrichment Score
regulation of apoptosis	46	2.38
heparin binding	12	1.9
ARID/BRIGHT DNA-binding domain	4	1.77
regulation of actin cytoskeleton organisation	8	1.7
melanocyte differentiation	4	1.6
negative regulation of apoptosis	22	1.59
collagen metabolic process	5	1.54
WD40 repeat	17	1.52
mesenchymal cell differentiation	6	1.39
cell migration	18	1.31
embryonic limb morphogenesis	8	1.25
Homeobox transcription factor	15	1.25
regulation of nucleotide metabolic process	9	1.25

Supplemental Table II
Functional annotation of differentially expressed genes.

	Gene symbol	Gene name	Low vs High WSS	Zebrafish orthologue(s)	% aa id
**1**	***ANGPTL4***	Angiopoietin-like 4	↗	*angptl4*	35
**2**	***CCL2***	Chemokine (C-C motif) ligand 2	↗	NONE	N/A
**3**	***CD74***	CD74 molecule, major histocompatibility complex, class II invariant chain	↘	*cd74a*<br>*cd74b*	29<br>24
**4**	***CDH13***	Cadherin 13	↘	*cdh13*	61
**5**	***CSF2***	Colony stimulating factor 2 (granulocyte-macrophage)	↗	NONE	N/A
**6**	***FADD***	Fas (TNFRSF6)-associated via death domain	↘	*fadd*	34
**7**	***IGF1***	Insulin-like growth factor 1	↗	*igf1*	54
**8**	***JUN***	Jun proto-oncogene	↘	*jun*	76
**9**	***LGALS1***	Lectin, galactoside-binding, soluble, 1	↗	*lgals1l1*<br>*lgals1l2*<br>*lgals1l3*	36<br>39<br>43
**10**	***PDCD2L***	Programmed cell death 2-like	↗	*pdcd2l*	40
**11**	***PERP***	p53 apoptosis effector related to PMP-22	↗	*perp*	48
**12**	***PTGIS***	Prostaglandin I2 (prostacyclin) synthase	↘	*ptgis*	48
**13**	***TNFSF10***	Tumor necrosis factor (ligand) superfamily, member 10	↘	*tnfsf10*	38
**14**	***TNIP1***	TNFAIP3 interacting protein 1	↗	*tnip1*	42

Supplemental Table III

Zebrafish orthologues of the mammalian apoptotic regulators studied. Red indicates upregulation and green indicates downregulation of gene expression in low versus high WSS region of the pig aortic arch. Zebrafish orthologue(s) are listed for each gene with percentage identities on amino acid (% aa id) between mammalian and fish orthologues.

	GENE	Serbanovic-Canic et al	Passerini et al
1	*ADORA3*	Down	Up
2	*AKAP7*	Down	Up
3	*APOA1*	Up	Up
4	*BBS4*	Down	Up
5	*C1QTNF1*	Up	Up
6	*CAMK2G*	Down	Down
7	*CCL2*	Up	Up
8	*CDH13*	Down	Down
9	*COL5A1*	Up	Up
10	*COL6A1*	Up	Down
11	*DCTD*	Down	Down
12	*FGF7*	Up	Down
13	*FXYD3*	Up	Down
14	*FZD8*	Down	Down
15	*GATA4*	Up	Up
16	*GPM6A*	Up	Up
17	*GPX3*	Up	Up
18	*HAND2*	Up	Up
19	*HDAC5*	Down	Down
20	*HOXA7*	Down	Down
21	*HSPA8*	Up	Up
22	*ISYNA1*	Up	Up
23	*JUN*	Down	Down
24	*KCNH2*	Up	Up
25	*LGALS1*	Up	Up
26	*LTBP1*	Up	Up
27	*MAX*	Down	Down
28	*MFAP1*	Down	Down
29	*MGST2*	Up	Up
30	*MKRN2*	Down	Down
31	*MT1A*	Up	Up
32	*PDGFRA*	Up	Up
33	*PEG10*	Up	Up
34	*PFN2*	Up	Up
35	*PPFIBP1*	Up	Down
36	*SLIT2*	Up	Up
37	*STK38L*	Down	Down
38	*TACSTD2*	Up	Up
39	*THRB*	Up	Up
40	*UGP2*	Down	Down
41	*ZNF238*	Down	Down

	GENE	Serbanovic-Canic et al	Civelek et al
1	*ANGPTL4*	Up	Up
3	*CCL2*	Up	Up
4	*COL3A1*	Up	Up
5	*FNBP1*	Down	Down
6	*GRB2*	Down	Up
7	*HNRPDL*	Up	Up
8	*LTBP1*	Up	Up
9	*MASP1*	Up	Up
10	*PROM2*	Up	Up
11	*PDCD2L*	Up	Up
12	*PROM2*	Up	Up
13	*TMEM9*	Up	Up
14	*TRAPPC4*	Down	Up
15	*UBQLN1*	Up	Up
16	*UGCG*	Up	Up

	GENE	Serbanovic-Canic et al	Ni et al
1	*ARHGAP25*	Down	Up
2	*ARL4A*	Down	Down
3	*CACHD1*	Up	Down
4	*CD74*	Down	Up
5	*CDC42EP5*	Up	Down
6	*CEP170*	Up	Up
7	*DYNC1I2*	Down	Down
8	*EPAS1*	Up	Down
9	*FZD4*	Up	Down
10	*GJA5*	Down	Down
11	*GPM6A*	Up	Up
12	*HOXA7*	Down	Down
13	*ITM2B*	Down	Down
14	*KIRREL3*	Up	Down
15	*PHACTR1*	Down	Up
16	*PKHD1L1*	Up	Down
17	*PLEK2*	Up	Down
18	*PNPLA6*	Up	Down
19	*SMAP2*	Down	Up
20	*SORT1*	Down	Down
21	*SP100*	Down	Down
22	*SULT1A1*	Up	Down
23	*SYP*	Up	Down
24	*TTC17*	Down	Down
25	*WWP2*	Down	Down

Supplemental Table IV

Comparison of microarray data with other *in vivo* microarray studies. Genes found to be differentially expressed both in the current study (Serbanovic-Canic et al) and in published studies from Passerini 2004,[13] Civelek 2009[14] and Ni 2010[15] are listed. Red indicates upregulation at low WSS or athero-prone sites; green indicates downregulation at low WSS or athero-prone sites.

	GENE	Serbanovic-Canic et al	Maimari et al
1	*ANGPTL4*	Up	Down
2	*APLN*	Up	Up
3	*APLNR*	Up	Down
4	*ARSJ*	Up	Up
5	*ATP8A1*	Down	Down
6	*BCHE*	Up	Up
7	*BDH1*	Up	Up
8	*C13orf33*	Up	Down
9	*C1orf21*	Up	Down
10	*CALML4*	Down	Down
11	*CCL2*	Up	Up
12	*CD79B*	Down	Down
13	*CHAF1A*	Down	Up
14	*CHEK1*	Down	Up
15	*CHN1*	Up	Down
16	*CHST3*	Up	Down
17	*COL5A1*	Up	Down
18	*COL6A1*	Up	Down
19	*CORO2B*	Down	Up
20	*CSF3*	Up	Up
21	*CXCL2*	Up	Up
22	*CXXC5*	Up	Down
23	*CYP2U1*	Down	Down
24	*ELMO2*	Down	Down
25	*ENO2*	Up	Down
26	*EPAS1*	Up	Down
27	*FLYWCH1*	Up	Down
28	*GIMAP4*	Up	Up
29	*GJA5*	Down	Down
30	*HDAC5*	Down	Down
31	*HIST1H1D*	Down	Up
32	*HYAL3*	Up	Down
33	*ITFG3*	Up	Down
34	*KIFC1*	Up	Up
35	*LIMCH1*	Down	Up
36	*LXN*	Down	Up
37	*MLYCD*	Up	Down
38	*MSN*	Up	Down

	GENE	Serbanovic-Canic et al	Maimari et al
39	*MTAP*	Down	Up
40	*MYH10*	Up	Up
41	*NAGA*	Down	Down
42	*NPPC*	Up	Down
43	*OAF*	Up	Down
44	*OIP5*	Up	Up
45	*PARP12*	Down	Up
46	*PDE4A*	Up	Down
47	*PLA1A*	Down	Down
48	*PLEK2*	Up	Down
49	*POC1A*	Down	Up
50	*PSAT1*	Up	Up
51	*RHOF*	Up	Down
52	*RNF144A*	Down	Up
53	*RNF214*	Down	Down
54	*ROGDI*	Up	Down
55	*SBNO2*	Up	Down
56	*SERPINB1*	Up	Down
57	*SFXN2*	Down	Up
58	*SLC2A3*	Up	Down
59	*SLC7A7*	Up	Up
60	*SORT1*	Down	Up
61	*SOS2*	Down	Down
62	*STK38L*	Down	Down
63	*SULT1A1*	Up	Down
64	*TACSTD2*	Up	Up
65	*TFF1*	Up	Down
66	*TMCC1*	Down	Down
67	*TNFSF10*	Down	Up
68	*TRAP1*	Up	Up
69	*TTC17*	Down	Down
70	*VEGFA*	Up	Down
71	*VRK1*	Down	Up
72	*WBP2*	Up	Down
73	*ZDHHC17*	Down	Down
74	*ZFAND2B*	Down	Down
75	*ZFHX3*	Down	Down
76	*ZNF22*	Up	Down

Supplemental Table V

Comparison of microarray data with *in vitro* microarray studies. Genes found to be differentially expressed both in the current study (Serbanovic-Canic et al) and in the meta-analysis of published *in vitro* microarray studies (Maimari 2016)[16] are listed. Red indicates upregulation at low WSS or athero-prone sites; green indicates downregulation at low WSS or athero-prone sites.

MO name	Position	MO sequence (5'-3')	MO dose used for functional study	Reference
**Control**	N/A	CCTCTTACCTCAGTTACAATTTATA	3 ng or 6 ng	Gene Tools standard control
angptl4	I2E3	TCGTAATCTAAAGGACATCACATGA	6 ng	This study
***angptl4* MO2**	E3I3	TCAGCAATGATAAACTGACTTACCA	6 ng	This study
cd74a	E1I1	AATCTACTCAATCACCTTGCATCAC	3 ng	This study
cd74b	E2I2	GTGCTATGGTTTTCTGACCAGATTT	6 ng	This study
cdh13	E1I1	GCACTGTAAACATCTTACCCTGAGT	3 ng	This study
***cdh13* MO2**	E4I4	TGTTAGGTCACAAAAGAGTACCTGT	3 ng	This study
fadd	I1E2	TTGATTCTCTCTAAAAAGAGACACA	N/A	This study
igf1	I1E2	GTACACTGCATCCCAGAGAGAGGCA	N/A	This study
jun	ATG	TTCCATCTTGGTAGACATAGAAGGC	3 ng	This study
p53	ATG	GCGCCATTGCTTTGCAAGAATTG	3 ng	(Langheinrich, Hennen et al. 2002)
pdcd2l	E2I2	TGAAATAACAGAATGCTACCTTTCT	3 ng	This study
***pdcd2l* MO2**	I1E2	AGGTCCTGTGAAGACAGAATTCAGC	6 ng	This study
perp	E1I1	CTGTACTTTTGACTTACAGAACTCC	6 ng	This study
***perp* MO2**	ATG	TCGATCCCACACCGGAACATTATGC	6 ng	This study
ptgis	E2I2	ATAACATGACCTTAGAACTCACTG	3 ng	This study
***sih* MO**	ATG	CATGTTTGCTCTGATCTGACACGCA	3 ng	(Sehnert, Huq et al. 2002)
tnfsf10	E1I1	TTAACTCCTCGGCCCTTACCGTCGA	N/A	This study
tnip1	I3E4	ATGACCTGAAGAGAGCCGCAACCAT	3 ng	This study

Supplemental Table VI

Morpholino sequences. Morpholinos (MOs) were designed to bind to the ATG region (translation-blocking MOs) or junctions between introns (I) and exons (E) of targeted genes. MO targeting *p53*[17] and *sih* MO[18] have been previously published.

Organism	Gene	Forward primer (5'-3')	Reverse primer (5'-3')	Purpose
Danio rerio	*angptl4*	ATGCAAGGAGAGGGTTTGGA	GCCATTCTGATATGCCTGCC	RT-PCR
Danio rerio	*angptl4*	AACAGAACATCCGCATCAGG	TCAGAGACAGTCGCTCGTTT	qRT-PCR
Danio rerio	*bact2*	TGAAATTGCCGCACTGGTTGTTG	ATGGCGGGGGTGTTGAAGGTC	RT-PCR
Danio rerio	*bact2*	AGCCATCCTTCTTGGGTATG	TGATCTCCTTCTGCATCCTG	qRT-PCR
Danio rerio	*cd74a*	CCAGACCACCAGAACGAGTC	CTGGTCAGAGCGTTGATGTG	RT-PCR
Danio rerio	*cd74b*	TCTAGCGAGGGAAACGAGAC	CGGAATTAAGGGCCCAACAC	RT-PCR
Danio rerio	*cdh13*	GATGACTGTGCTGGGAATGA	AATGGAGCTCGCTGGTTCT	RT-PCR
Danio rerio	*fadd*	AGACCGAGAGTTGAAAGCGA	CAGCGGTGAGGTTCTGTTTG	RT-PCR
Danio rerio	*fadd*	ACGATTATGACAGGCAAACGT	GCTGTGTTGATTCTCTCTCGC	qRT-PCR
Danio rerio	*igf1*	GACATTGCCCGCATCTCATC	AGATGGGGCTTAAACGTCCA	RT-PCR
Danio rerio	*pdcd2l*	ATGGCGACATCAATACACGA	TCACTCTCTCCCTTTCCTCGT	RT-PCR
Danio rerio	*perp*	GGTGTGGGATCGACTACACG	ATGAAGGGCAGCAGAGAGAC	RT-PCR
Danio rerio	*perp*	TCATGGAGTTCTCTTGGGCT	TGATGAAGGCGAAGATGAGGA	qRT-PCR
Danio rerio	*ptgis*	TCTGCTGGTGGGACTCTCTA	ATAAGCAGGCGGAGGTTGTT	RT-PCR
Danio rerio	*tnfsf10*	ACTTCAGCAACGTCTTGTCG	CACTCGATCTCCACTTCCGA	RT-PCR
Danio rerio	*tnip1*	TGCCTCCAGTCTGTGTATGG	TGGTTGGGATTGGACTCTTCA	RT-PCR
Danio rerio	*tnip1*	CACACGGTCATGGAAAACGT	TCCCAGTCAAACAATCCCGA	qRT-PCR
Homo sapiens	*ANGPTL4*	GACCCTGAGGTCCTTCACAG	CTTGTGGAAGAGTTGCTGGA	qRT-PCR
Homo sapiens	*CCL2*	TCAAACTGAAGCTCGCACTC	ATTGATTGCATCTGGCTGAG	qRT-PCR
Homo sapiens	*CD74*	TGCAGAATGCCACCAAGTAT	TCAGCATTCTGGAGCAGGT	qRT-PCR
Homo sapiens	*CDH13*	TTCTGTTGTCAAACCATTGGA	GGGTACGAGTGGGTCTTCAT	qRT-PCR
Homo sapiens	*CSF2*	CCCTGGGAGCATGTGAAT	TTTCATTCATCTCAGCAGCA	qRT-PCR
Homo sapiens	*FADD*	GGAAGAAGACCTGTGTGCAG	TTGGTGTCTGAGACTTTGAGC	qRT-PCR
Homo sapiens	*HPRT*	TTGGTCAGGCAGTATAATCC	GGGCATATCCTACAACAAAC	qRT-PCR
Homo sapiens	*IGF1*	TATTTCAACAAGCCCACAGG	TCCAGCCTCCTTAGATCACA	qRT-PCR
Homo sapiens	*JUN*	TTCCTGCGTCTTAGGCTTCT	ACGTCCTTCTTCTCTTGCGT	qRT-PCR
Homo sapiens	*LGALS1*	TCAATCATGGCTTGTGGTCT	CACGAAGCTCTTAGCGTCAG	qRT-PCR
Homo sapiens	*PDCD2L*	GACTGGTGTGAAGGTGCTGA	CGAGCAGTCCAGTCTACGTC	qRT-PCR
Homo sapiens	*PERP*	GCCAACCCTGCTGTCACTTA	GAAGGCACAGCCAATCAGGA	qRT-PCR
Homo sapiens	*PTGIS*	GGCCAGGATGAAACTGACTC	CACTGCATGGAGGTTGGTAT	qRT-PCR
Homo sapiens	*TNFSF10*	TGCTGATCGTGATCTTCACA	AAGAAACAAGCAATGCCACTT	qRT-PCR
Homo sapiens	*TNIP1*	GAAGTGAACAAGCAGTGGGA	GCAGCTCAGTGATCTTCTGC	qRT-PCR
Sus scrofa	*ANGPTL4*	ACTGCCAAGAGCTGTTTGAAGA	CCATCTGAGGTCATCTTGCAGTT	qRT-PCR
Sus scrofa	*ARHGAP25*	AGCAGAGGTCCATCGTGAAA	ACATGTAACCCTGTGGCTTCA	qRT-PCR
Sus scrofa	*B2M*	TTCACTCCTAACGCTGTGGA	GTGGTCTCGATCCCACTTAAC	qRT-PCR
Sus scrofa	*CCL2*	TCACCTGCTGCTATACACTTAC	ATCACTGCTTCTTTAGGACACTTG	qRT-PCR
Sus scrofa	*CD74*	TTTGAGCAAGATGCGGGTTT	GGTCATGTTGCCGTACTTGG	qRT-PCR
Sus scrofa	*CDH13*	AGACATTGTCACCGTCGTGT	TCCAGTCCAGCCATATCTTG	qRT-PCR
Sus scrofa	*CDKN2AIP*	TCCGAGGGGCGTTCTCTGGA	GCTGCACCGAAAAGGTGGCG	qRT-PCR
Sus scrofa	*CSF2*	CAGCGGCTGTGATGAATGAA	TTGTACAGGTTCAGGCGAGT	qRT-PCR
Sus scrofa	*ERCC5*	AGGCAATATGAAGATGAAGGG	GCCGCTTTCTTAGCTTGAAT	qRT-PCR
Sus scrofa	*FADD*	GTCGGCCAGCCTGTCAAGCA	AGCGCAGTGTGCTCGGGACT	qRT-PCR
Sus scrofa	*HIF1A*	CGAAGGGTTTCCCGCCTCGC	CTAAGCGCTGGCTCCCTCCG	qRT-PCR
Sus scrofa	*IGF1*	TGCACCCCTCAAGCCTGCCA	TGAGCCTTGGGCATGTCCGT	qRT-PCR
Sus scrofa	*JUN*	GAAAAGGAAGCTGGAGAGGAT	CTGCTGCGTTAAGCATGAGTT	qRT-PCR
Sus scrofa	*LGALS1*	AGACCTCACCATCAAGCTGC	GCTTCACTCAAAGGCCACAC	qRT-PCR
Sus scrofa	*PDCD2L*	AGCAAGCTAGGCGGCGTTCC	AGCGAACACGTGCAGGAGCC	qRT-PCR
Sus scrofa	*PERP*	TCCCCGCGGCTTCTTCCCTT	CAGCGCAGCATCTCGACGGT	qRT-PCR
Sus scrofa	*PTGIS*	CCGCTAGCTTCCTCACTAGG	AGGAGGACGGTGACATACCT	qRT-PCR
Sus scrofa	*TAF9*	CCACACTTGGCACACCAACCCA	GCTGGGGACTGTGAAGTGGGC	qRT-PCR
Sus scrofa	*TNFSF10*	CAGTCAGACCCTGCCTGGACC	ACACAGGGCTTGCAGGAGCA	qRT-PCR
Sus scrofa	*TNIP1*	AGCCGCTCCCATCACCGTCT	GCTCTGGCTTGCCACTGGGG	qRT-PCR

Supplemental Table VII

Antibody	Type	Dilution	Application	Source; catalogue number
Active caspase 3	Rabbit polyclonal IgG	1:200	Zebrafish whole-mount immunofluorescence	R&D Systems; AF835
Active caspase 3	Rabbit polyclonal IgG	1:100	HUVECs immunofluorescence	Cell Signaling; 9661
CD31	Rat polyclonal IgG	1:100	*En face* staining of murine aorta	BioLegend; 102514
CDH13	Rabbit polyclonal IgG	1:50	*En face* staining of murine aorta	Santa Cruz Biotechnology; sc-7940
PERP	Rabbit polyclonal IgG	1:50	*En face* staining of murine aorta	Santa Cruz Biotechnology; sc-67184

Supplemental Table VIII
List of used antibodies.
