# Supplementary material for: Zebrafish Model for Functional Screening of Flow-Responsive Genes
Source: Arterioscler Thromb Vasc Biol. 2016 Dec 21;37(1):130–43. doi: 10.1161/ATVBAHA.116.308502 (PMC5172514; doi:10.1161/ATVBAHA.116.308502)
Supplement: Supplementary file 2 [file atv-37-130-s002.pdf]

# MATERIALS AND METHODS

## Zebrafish studies

All zebrafish studies conformed to the institution's ethical requirements and UK Home Office regulations. Maintenance, manipulation and staging of wildtype and transgenic lines (*phd3:GFP* (kindly provided by Dr Freek van Eeden, University of Sheffield),[1] *flk1:EGFP-NLS*[2] and *kdrl:HRAS-mCherry*[3]) were carried out as described previously.[4, 5] ECs were isolated by fluorescence-activated cell sorting (FACS) from dissociated *flk1:EGFP-NLS* embryos as described previously.[6] Morpholino antisense oligonucleotides (MOs) (GeneTools, LLC) were diluted in sterile water and ~1 nl was injected into the yolk of a 1-4 cell stage embryo. The list of MOs used in this study is presented in Table S6. The efficiency of splice-blocking MOs was validated by reverse transcriptase-PCR (RT-PCR) and quantitative RT-PCR (qRT-PCR). Total RNA was extracted from zebrafish embryos using RNeasy Mini Kit (QIAGEN) according to manufacturer's protocol and 500 ng of total RNA was subjected to cDNA synthesis using iScript reverse transcriptase (Bio-Rad). Resulting cDNA was used as a template for PCR using gene-specific primers (listed in Table S7) and BioMix Red kit (Bioline) as per manufacturer's instructions. The resulting RT-PCR products were analysed by agarose gel electrophoresis. MO-injected samples in which a considerable wildtype band was visible on the gel were subjected to qRT-PCR analysis of gene expression to further validate the efficiency of the knockdown (primers listed in Table S7). Amplification of housekeeping gene *beta-actin* (*bact2*) was used as an internal control. To arrest heartbeat pharmacologically, embryos were treated with 0.65 mg/ml tricaine (Sigma-Aldrich) in E3 embryo medium from 22 hours post fertilisation (hpf) until 30 hpf. Apoptotic cells in whole-mount zebrafish embryos were detected by active caspase 3 staining (Table S8)[7] and terminal deoxynucleotidyl transferase-mediated dUTP nick end labelling (TUNEL) assay (In Situ Cell Death Detection Kit – TMR Red, Roche). To control for specific binding, isotype-matched IgG control was used for active caspase 3 antibody, whereas for TUNEL assay the embryos were incubated with label solution only (without terminal transferase) (data not shown). Imaging was performed using an Olympus FV1000 laser scanning confocal microscope with a 40x (numerical aperture (NA) 1.0) oil immersion objective. Endothelial cell (EC) apoptosis was quantified by counting the total number of ECs ($GFP^+$ cells) and the number of apoptotic ECs ($GFP^+$ and active caspase $3^+$/$TUNEL^+$ cells; yellow cells). The proportion of apoptotic cells was obtained by dividing the number of apoptotic cells by the total number of cells.

## Large animal husbandry and magnetic resonance imaging

Animal care and experimental procedures were carried out under licenses issued by the UK Home Office and local ethical committee approval was obtained. Five female landrace pigs (30-40 kg) were studied using MR at Hammersmith Campus, Imperial College London. Pigs (female, Landrace strain, aged 6 months) were sedated using Ketamine (5 mg/kg; intramuscular injection) with Xylazine (1 mg/kg; intramuscular injection), intubated and anaesthesia was maintained by inhalation (Isofluorane/O2) during the scan (approximately 6 hours). Aortic geometries were acquired by ECG-gated Black Blood Turbo Spin Echo vessel-wall imaging MR using a 3T whole body MR scanner (Siemens) with ECG-gating.

Approximately 12 cm of the aorta was imaged with in-plane spatial resolution of 0.976x0.976 $mm^2$ and slice thicknesses of 3 mm. 3D Phase Contrast (PC) MR imaging was used to measure blood flow in the region of interest. The encoding gradient for through-plane velocity was 120 cm/s, while the in-plane velocity encoding gradient was 60 cm/s. The three dimensional velocity was acquired at 30 time points equally spaced over the cardiac cycle on planes positioned immediately distal to the aortic valve, in the descending aorta and in the branches of the aortic arch. The velocity profile was then derived from the pixel intensity of the phase contrast MR images. Time-of-Flight MR Imaging was also performed to acquire reference magnitude images for the velocity-encoded measurements. Animals were euthanized using a lethal intravenous injection of pentobarbital (>150mg/kg).

**Computational fluid dynamics**

Geometries were reconstructed using Black Blood MR images imported into the commercial software Amira (Visage Imaging Inc.) and segmented using an intensity threshold-based method (or manually when vessel contours were not adequately defined by the automatic procedure). An intensity isosurface was determined using the open-source software VMTK (www.vmtk.org) as follows: (1) the surface was smoothed with a low-pass filter (frequency=0.1, iterations=30) to remove irregularities introduced during the scan and segmentation process, (2) flow extensions were added from each outflow vessel (length=20÷30 diameters), (3) an additional low-pass filter (frequency=0.1, iterations 20) was applied to smooth the final surface with the flow extensions. An interior mesh of $8x10^5$ to $3x10^6$ tetrahedral elements was generated in Gambit 2.4.6 (ANSYS, Inc.) then the volume mesh was generated with Tgrid (ANSYS, Inc). The flow at the inlet of the geometry was defined from velocity profile measurements obtained from MR imaging. In steady state simulations, the velocity was averaged over the cardiac cycle and the mean value was used. The flow split ratio between the descending thoracic aorta and the branches was based on MR flow measurements (Supplemental Fig. XVIII). Flow was assumed to be fully developed at the outlet of the aortic arch and carotid arteries. Vessel walls were assumed to be non-permeable and rigid. A no-slip condition was applied. The Navier-Stokes equations were solved using Fluent 6.2 (ANSYS Inc, Pennsylvania, US). The simulations were performed under steady and unsteady conditions. Time-dependent solutions were obtained using an implicit scheme. The PISO formulation was used for pressure-velocity coupling and the second-order upwind method was applied to solve the equations at each time step. Blood was assumed to be an incompressible, Newtonian fluid with constant density 1040 $kg/m^3$ and dynamic viscosity 0.0035 Pa/s. Blood flow in the porcine aorta was modelled for five animals (Fig. 2 and Supplemental Fig. IV). Velocity profiles at the inlet of the ascending aorta were assumed to be skewed to account for radial and axial components of the flow[8, 9] as revealed by phase contrast MR imaging. The time-varying waveform was imposed at the inlet for unsteady simulations (Reynolds number = 2635), while the time-averaged velocity (calculated over the cardiac cycle) was imposed for steady simulations (Reynolds numbers were 958, 1282, 1105, 964).

**Endothelial cell isolation, microarrays and bioinformatics**

Aortae were cut longitudinally along the outer curvature to expose the lumen. Regions exposed to high or low WSS were identified by reference to a 2 dimensional version of the shear stress map generated using an in-house code (Matlab R2008b, MathWorks) and visualized using Tecplot 360 (Tecplot Inc, Washington, US; Supplemental Fig. IV). ECs were harvested from high and low WSS regions by incubating portions of the lumen with collagenase (1 mg/ml for 10 minutes at room temperature) prior to gentle scraping. RNA was extracted using an RNeasy Mini Kit (Qiagen) and concentrated using RNAeasy MinElute Cleanup Kit (Qiagen). The purity and integrity of total RNA samples was assessed using a Bioanalyser (Agilent). High-quality samples were used to generate labelled cRNA using a 3' IVT Express Kit (Affymetrix). Labelled cRNA was hybridized to GeneChip® Porcine Genome Arrays (Affymetrix) using standardised procedures and signals were analysed with a GeneChip Scanner 3000. Data were analysed using GeneSpring software GX 11 (Agilent), DAVID (http://david.abcc.ncifcrf.gov) and Ingenuity Pathway Analysis (http://www.ingenuity.com/).

**Studies of cultured ECs and gene silencing**

HUVECs and PAECs were isolated using collagenase digestion as described previously,[10] cultured on 1% gelatin and maintained in M199 growth medium supplemented with foetal bovine serum (20%), L-glutamine (4 mmol/L), endothelial cell growth supplement (30 μg/ml), penicillin (100 U/ml), streptomycin (100 μg/ml) and heparin (10 IU/ml). ECs at passage 3-5 were exposed to flow using two complementary systems. Cells cultured in 6 well-plates were placed on an orbital rotating platform (210 rpm) inside a cell culture incubator (5% CO2, 37°C) for 72 h to generate low oscillatory WSS at the centre of the well, and higher unidirectional WSS at the periphery. Alternatively, ECs were cultured on microslides and exposed to high (13 dynes/cm$^2$) or low oscillatory (±4 dynes/cm$^2$, 1 Hz) for 72 h using a commercial parallel-plate system (ibidi). RNA interference was performed using small interfering (si) RNA sequences (ANGPTL4 (L-007807-00-0005, GE Dharmacon), CDH13 (L-003878-00-0005 (GE Dharmacon), PERP (sc-61326, Santa Cruz Biotechnology), PDCD2L (sc-97629, Santa Cruz Biotechnology). A non-targeting scrambled sequence (Ambion® *Silencer®* Negative Control #1) was used as a control. Transcript levels were determined by qRT-PCR using gene-specific primers listed in Table S7.

**Fluorescent staining of cultured cells and *en face* staining of murine endothelium**

The expression levels of proteins were assessed by immunostaining using specific antibodies (listed in Table S8) followed by confocal microscopy. ECs were fixed with formaldehyde (4%) and permeabilised with Triton X-100 (0.1%). Following blocking with goat serum for 1 h monolayers were incubated with primary antibody against cleaved caspase-3 (Cell Signalling), followed by AlexaFluor 488-conjugated secondary antibody. Alternatively, terminal Transferase dUTP Nick End Labeling (TUNEL) was used to detect DNA degradation in apoptotic cells as described. Nuclei were counter-stained using To-Pro-3 (Invitrogen). Fluorescence was measured using a Zeiss LSM 510 META laser-scanning confocal microscope and mean fluorescence intensities (MFI) were averaged for multiple cells in multiple fields of view using Zeiss LSM 510 META image analysis software. The expression levels of specific proteins were assessed at regions of murine aortic arch exposed to high (outer curvature) or low WSS (inner curvature) by *en face* staining as

described previously.[11, 12] Aortae of C57BL/6 mice were perfused *in situ* with PBS (at a pressure of approximately 100 mm Hg) and then perfusion-fixed using 4% formalin prior to harvesting. Fixed aortic segments were tested by immunostaining using primary anti-CDH13 and anti-PERP antibodies and Alexa Fluor 568-conjugated secondary antibodies (red). ECs were identified by co-staining using anti-CD31 antibody conjugated to Alexa Fluor 488 (green). Nuclei were identified using To-Pro-3 (Invitrogen). Stained vessels were mounted in ProLong Gold Antifade mountant (life technologies) prior to visualisation of endothelial surface *en face* using confocal scanning microscopy (Zeiss LSM 510 NLO inverted microscope). The protein expression was assessed by quantification of fluorescence intensity for multiple cells (at least 50 per site) using Fiji software and calculation of mean fluorescence intensities with standard error of the mean. To control for specific binding, isotype-matched IgG control antibodies (abcam) were used (Supplemental Fig. XII and not shown).

**Statistical analysis**

Data are expressed as the average of individual experiments with standard deviation or standard error of the mean, as indicated. After testing that the data complied with the constraints of parametric analysis (D'Agostino-Pearson omnibus test), differences between two groups were assessed with an unpaired two-tailed Student's *t*-test. One-way analysis of variance (ANOVA) with Tukey's multiple comparisons post-hoc test was used to compare differences between more than two groups; in some cases the unequal variance correction was applied. The effects of multiple factors on the data were evaluated by two-way ANOVA with Tukey's multiple comparison post-hoc test. Differences were considered statistically significant when p-values $<0.05$.

## SUPPLEMENTARY REFERENCES


1. Santhakumar K, Judson EC, Elks PM, McKee S, Elworthy S, van Rooijen E, Walmsley SS, Renshaw SA, Cross SS, van Eeden FJ. A zebrafish model to study and therapeutically manipulate hypoxia signaling in tumorigenesis. *Cancer Res*. 2012;72:4017-4027

2. Blum Y, Belting HG, Ellertsdottir E, Herwig L, Luders F, Affolter M. Complex cell rearrangements during intersegmental vessel sprouting and vessel fusion in the zebrafish embryo. *Dev Biol*. 2008;316:312-322

3. Hogan BM, Bos FL, Bussmann J, Witte M, Chi NC, Duckers HJ, Schulte-Merker S. Ccbe1 is required for embryonic lymphangiogenesis and venous sprouting. *Nat Gen*. 2009;41:396-398

4. Kimmel CB, Ballard WW, Kimmel SR, Ullmann B, Schilling TF. Stages of embryonic development of the zebrafish. *Dev Dynamics.* 1995;203:253-310

5. Westerfield M. The zebrafish book. A guide for the laboratory use of zebrafish (danio rerio). *4th ed., Univ. of Oregon Press, Eugene*. 2000

6. Covassin L, Amigo JD, Suzuki K, Teplyuk V, Straubhaar J, Lawson ND. Global analysis of hematopoietic and vascular endothelial gene expression by tissue specific microarray profiling in zebrafish. *Dev Biol*. 2006;299:551-562

7. Gray C, Bratt D, Lees J et al. Loss of function of parathyroid hormone receptor 1 induces notch-dependent aortic defects during zebrafish vascular development. *Arterioscler Thromb Vasc Biol*. 2013;33:1257-1263

8. Shahcheraghi N, Dwyer HA, Cheer AY, Barakat AI, Rutaganira T. Unsteady and three-dimensional simulation of blood flow in the human aortic arch. *J Biomech Eng*. 2002;124:378-387

9. Kilner PJ, Yang GZ, Mohiaddin RH, Firmin DN, Longmore DB. Helical and retrograde secondary flow patterns in the aortic arch studied by three-directional magnetic resonance velocity mapping. *Circulation*. 1993;88:2235-2247

10. Jaffe EA, Nachman RL, Becker CG, Minick CR. Culture of human endothelial cells derived from umbilical veins. Identification by morphologic and immunologic criteria. *J Clin Invest*. 1973;52:2745-2756

11. Warboys CM, de Luca A, Amini N et al. Disturbed flow promotes endothelial senescence via a p53-dependent pathway. *Arterioscler Thromb Vasc Biol.* 2014;34:985-995

12. Cuhlmann S, Van der Heiden K, Saliba D, Tremoleda JL, Khalil M, Zakkar M, Chaudhury H, Luong le A, Mason JC, Udalova I, Gsell W, Jones H, Haskard DO, Krams R, Evans PC. Disturbed blood flow induces rela expression via c-jun n-terminal kinase 1: A novel mode of nf-kappab regulation that promotes arterial inflammation. *Circ Res*. 2011;108:950-959

13. Passerini AG, Polacek DC, Shi C, Francesco NM, Manduchi E, Grant GR, Pritchard WF, Powell S, Chang GY, Stoeckert CJ, Jr., Davies PF. Coexisting proinflammatory and antioxidative endothelial transcription profiles in a disturbed flow region of the adult porcine aorta. *Proc Natl Acad Sci USA*. 2004;101:2482-2487

14. Civelek M, Manduchi E, Riley RJ, Stoeckert CJ, Jr., Davies PF. Chronic endoplasmic reticulum stress activates unfolded protein response in arterial endothelium in regions of susceptibility to atherosclerosis. *Circ Res*. 2009;105:453-461

15. Ni CW, Qiu H, Rezvan A, Kwon K, Nam D, Son DJ, Visvader JE, Jo H. Discovery of novel mechanosensitive genes in vivo using mouse carotid artery endothelium exposed to disturbed flow. *Blood*. 2010;116:e66-73

16. Maimari N, Pedrigi RM, Russo A, Broda K, Krams R. Integration of flow studies for robust selection of mechanoresponsive genes. *Thromb Haemost*. 2016;115

17. Langheinrich U, Hennen E, Stott G, Vacun G. Zebrafish as a model organism for the identification and characterization of drugs and genes affecting p53 signaling. *Curr Biol : CB*. 2002;12:2023-2028

18. Sehnert AJ, Huq A, Weinstein BM, Walker C, Fishman M, Stainier DY. Cardiac troponin t is essential in sarcomere assembly and cardiac contractility. *Nat Gen*. 2002;31:106-110
